# Germline viral “fossils” guide *in silico* reconstruction of a mid-Cenozoic era marsupial adeno-associated virus

**DOI:** 10.1038/srep28965

**Published:** 2016-07-05

**Authors:** Richard H. Smith, Claus V. Hallwirth, Michael Westerman, Nicola A. Hetherington, Yu-Shan Tseng, Sylvain Cecchini, Tamas Virag, Mona-Larissa Ziegler, Igor B. Rogozin, Eugene V. Koonin, Mavis Agbandje-McKenna, Robert M. Kotin, Ian E. Alexander

**Affiliations:** 1 Laboratory of Molecular Virology and Gene Therapy, National Heart, Lung and Blood Institute, Bethesda, Maryland, United States of America; 2 Gene Therapy Research Unit, Children’s Medical Research Institute and The Children’s Hospital at Westmead, Westmead, New South Wales, Australia; 3 Department of Biochemistry and Genetics, La Trobe University, Bundoora, Victoria, Australia; 4 The McKnight Brain Institute, University of Florida, Gainesville, Florida, United States of America; 5 Evolutionary Genomics Research Group, National Center for Biotechnology Information, National Library of Medicine, National Institutes of Health, Bethesda, Maryland, United States of America; 6 The University of Sydney, Discipline of Paediatrics and Child Health, Westmead, New South Wales, Australia

## Abstract

Germline endogenous viral elements (EVEs) genetically preserve viral nucleotide sequences useful to the study of viral evolution, gene mutation, and the phylogenetic relationships among host organisms. Here, we describe a lineage-specific, adeno-associated virus (AAV)-derived endogenous viral element (mAAV-EVE1) found within the germline of numerous closely related marsupial species. Molecular screening of a marsupial DNA panel indicated that mAAV-EVE1 occurs specifically within the marsupial suborder Macropodiformes (present-day kangaroos, wallabies, and related macropodoids), to the exclusion of other Diprotodontian lineages. Orthologous mAAV-EVE1 locus sequences from sixteen macropodoid species, representing a speciation history spanning an estimated 30 million years, facilitated compilation of an inferred ancestral sequence that recapitulates the genome of an ancient marsupial AAV that circulated among Australian metatherian fauna sometime during the late Eocene to early Oligocene. *In silico* gene reconstruction and molecular modelling indicate remarkable conservation of viral structure over a geologic timescale. Characterisation of AAV-EVE loci among disparate species affords insight into AAV evolution and, in the case of macropodoid species, may offer an additional genetic basis for assignment of phylogenetic relationships among the Macropodoidea. From an applied perspective, the identified AAV “fossils” provide novel capsid sequences for use in translational research and clinical applications.

In the absence of a fossil record and with little obvious interordinal molecular homology, information concerning the macroevolution and paleontological history of viruses has, until recently, been limited to extrapolation from observable genetic diversity among extant viral species. New insight into the long-term evolutionary history of viruses has been provided by the recent identification of a variety of genetically integrated, inheritable viral sequences, referred to as endogenous viral elements (EVEs), in the genomes of numerous plant and animal species^1^,^2^,^3^,^4^,^5^,^6^,^7^. These “fossilised” genetic elements represent ancient viral integration events within host DNA.

The majority of DNA animal virus-derived EVEs have, for unknown reasons, originated from members of the families *Parvoviridae* and *Circoviridae*. Both viral families encode an endonuclease essential for viral genomic DNA replication that might facilitate non-homologous molecular recombination with host genomic sequences. The *Parvoviridae*, in particular, have been the subject of considerable research over the past several decades. The current proposed taxonomy of the *Parvoviridae* includes two subfamilies: i) the *Parvovirinae*, consisting of eight genera infecting vertebrates ranging from birds to humans, and ii) the *Densovirinae,* consisting of five genera infecting arthropods^8^. The genus *Dependoparvovirus* of the subfamily *Parvovirinae* is particularly well represented within the DNA fossil record^1^,^3^,^6^,^9^.

Dependoparvoviruses, represented primarily by adeno-associated viruses (AAVs), comprise an expanding assemblage of recognised viral species, serotypes, and type-specific viral sequences isolated from vertebrate hosts, including humans, non-human primates, goats, cattle, bats, birds, and sea lions^10^,^11^,^12^,^13^,^14^,^15^,^16^. AAVs are among the smallest of the animal DNA viruses, with a ~5-kb genome packaged within a ~26-nm non-enveloped, icosahedral capsid. The typical AAV genome contains two protein-encoding genes termed *rep* and *cap* that, respectively, encode nonstructural proteins essential for viral genome replication and structural proteins that form the viral capsid. Putative accessory factors encoded by minor open reading frames (ORFs), such as the *aap* gene that encodes an assembly-activating protein (AAP), were recently described^17^,^18^. The AAV genomic coding region is flanked by inverted terminal repeat sequences (ITRs) that serve as the viral origins of replication and provide *cis*-acting elements required for packaging of progeny genomes^19^,^20^. Primate AAV serotypes were shown to establish a latent infection by integrating within the host cell genome^21^,^22^,^23^,^24^. In humans, *in silico* analysis of whole-genome sequence data has failed to detect evidence of AAV germline endogenisation; however, AAV-derived EVEs have been identified in non-human primates, as well as at least sixteen additional mammalian species, including representatives of the monotreme, metatherian and eutherian lineages^1^,^3^,^9^.

In this report, we describe an evolutionarily conserved marsupial locus (“mAAV-EVE1”) containing a near viral-genome-length AAV-derived EVE that is phylogenetically restricted to macropodoid marsupials. Based upon the distribution of mAAV-EVE1 sequences within the marsupial order Diprotodontia (to which the macropodoids belong), endogenisation occurred between 45 and 27 million years ago (MYA). To elucidate the genomic structure and coding capacity of a mid-Cenozoic AAV, maximum likelihood estimation of an mAAV-EVE ancestral sequence was performed using orthologous mAAV-EVE1 sequences from 16 macropodoid species. We present phylogenetic analysis and structural modelling of an AAV that likely circulated among susceptible marsupial hosts during the late Eocene to early Oligocene.

## Results

### Identification and phylogenetic mapping of a lineage-specific endogenous parvoviral element

In an attempt to isolate AAV sequences from a representative marsupial for the purpose of identifying novel capsid sequences, a PCR-based approach was used to screen tissue samples obtained from the eastern grey kangaroo, *Macropus giganteus*. Genomic DNA from a cohort of animals was subjected to PCR analysis using primers recognising highly conserved regions of the AAV genome^25^,^26^. Amplicon sequencing revealed highly significant sequence similarity to numerous dependoparvovirus genomes, as well as an endogenised AAV element previously identified within the genome of the tammar wallaby, *Macropus eugenii*^1^. Among the extant AAVs, the primate AAV serotypes, AAVhu.S17 and AAV13, displayed the most significant similarity to the PCR amplicon sequence, with E values of 10^−49^ and 10^−48^, respectively. Linker-mediated PCR (LM-PCR) using primers specific to the amplified marsupial AAV sequences in combination with linker-specific primers was used to isolate adjacent endogenous viral sequences. This process was reiterated in a “genome walking” approach to obtain the entirety of the integrated AAV element. Analysis of the full-length endogenous AAV sequences revealed several stop codons and frameshifts scattered throughout the viral *rep* and *cap* genes, and an absence of the viral ITRs. A GenBank query using the full-length *M. giganteus*-derived AAV sequence indicated that it was 96.4% identical to the longest of eleven AAV-EVEs previously identified with the *M. eugenii* genome^1^. The relevant AAV-EVE-containing *M. eugenii* genome assembly contig (ABQO010585939) initiates within the 5′ boundary of the *M. giganteus* AAV-EVE and is therefore devoid of upstream flanking genomic sequences. However, the downstream flanking genomic sequences of the *M. eugenii* genome assembly contig demonstrated a near-perfect match (only six transitions in 500 bp) with the downstream genomic flanking region in *M. giganteus*, suggesting that mAAV-EVE1 occupies an orthologous locus in these two macropods and that the AAV endogenisation event occurred in a common ancestor of these species.

**Figure 1:**
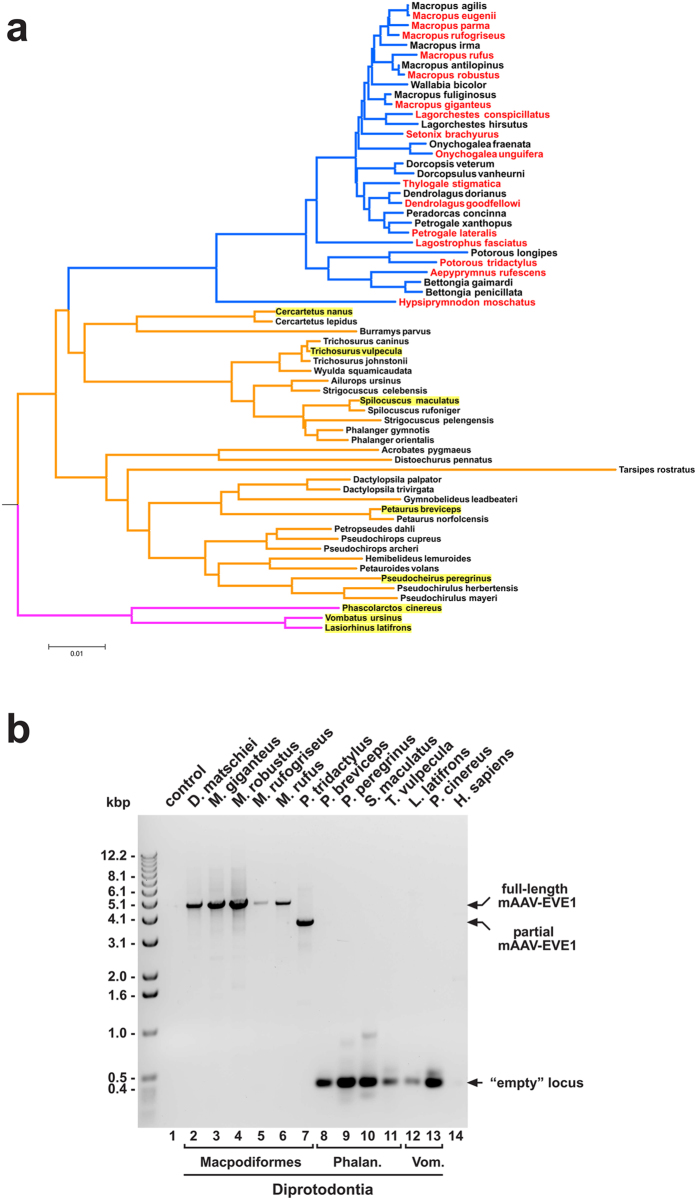
Mapping of mAAV-EVE1 to the diprotodontian phylogenetic tree. (**a**) A maximum likelihood phylogenetic tree was constructed using concatenated nuclear gene segments representing exonic portions of the *ApoB*, *BRCA1*, *IRBP*, *Rag1*, and *vWF* genes of sixty-four members of the order Diprotodontia^28^ and three eutherian outgroup members, *Bradypus tridactylus*, *Lama glama*, and Elephantidae (outgroup not shown). The nuclear gene segments were concatenated, aligned with MUSCLE^80^, and used to construct a maximum likelihood tree employing a GTR + G (General Time Reversible with gamma distributed rates among sites and five discrete gamma categories) substitution model as implemented in MEGA^29^. Number of bootstraps = 500. Select marsupial species were tested for mAAV-EVE1 status by PCR analysis as described in Methods. Red font indicates positive mAAV-EVE1 status, whereas yellow-highlighted font indicates negative mAAV-EVE1 status. Blue branches: suborder Macropodiformes. Orange branches: suborder Phalangeriformes. Magenta branches: suborder Vombatiformes. (**b**) The mAAV-EVE1 locus was PCR-amplified from a variety of marsupial species representative of the three suborders of Diprotodontia: Macropodiformes, Phalangeriformes (Phalan.) and Vombatiformes (Vom.) using primers to conserved genomic flanking sequences as described in Methods. PCR amplicons were resolved on a 1% agarose gel and visualised by staining with ethidium bromide (an ultraviolet fluorescent image is shown).

BLAST^27^ searches using the *M. giganteus* AAV-EVE genomic flanking sequences revealed the presence of homologous sequences in the available marsupial genome assemblies, *viz.* the gray short-tailed opossum (*Monodelphis domestica*) and the Tasmanian devil (*Sarcophilus harrisii*), both of which lack an AAV-EVE at this locus. Using homology with the upstream flanking sequence and the first 342 bp of mAAV-EVE1 from *M. giganteus*, a read with an equivalent sequence region was identified among the trace archives from the *M. eugenii* genome sequencing project (courtesy of Tony Papenfuss, Walter & Eliza Hall Institute of Medical Research, Melbourne, Australia). Alignment of the conserved upstream and downstream flanking sequences from *M. giganteus*, *M. eugenii*, *S. harrisii* and *Monodelphis domestica* facilitated the design of PCR primers to identify and isolate potential orthologs of mAAV-EVE1 within related marsupial species and map the occurrence of mAAV-EVE1 to the marsupial phylogenetic tree. DNA samples representing all three suborders and eight of the eleven families within the marsupial order Diprotodontia were queried (Fig. 1a). PCR results obtained from members of the three suborders, Macropodiformes (kangaroos, wallabies, bettongs/potoroos, and the musky rat-kangaroo), Phalangeriformes (possums and gliders) and Vombatiformes (koala and wombats) revealed that mAAV-EVE1 is lineage-restricted, occurring solely within the suborder Macropodiformes. The mAAV-EVE1 sequences were detected within genomic DNA samples isolated from the basal taxon of the macropodoid lineage*, Hypsiprymnodon moschatus*, and appear ubiquitously inherited throughout the superfamily Macropodoidea. Sequencing of cloned PCR amplicons recovered from sixteen macropodoid species indicated that two distinct classes of AAV-derived EVE occupy the mAAV-EVE1 locus: i) those representing near genome-length AAV-EVEs spanning the entirety of the AAV *rep* and *cap* genes (recovered exclusively from the family Macropodidae), but lacking recognisable viral ITRs; and ii) AAV-EVEs displaying relatively large (>1 kbp) internal deletions of EVEs (recovered from the families Potoroidae and Hypsiprymnodontidae) (Fig. 1b). Therefore, major post-integration deletions within mAAV-EVE1 sequences occurred at least twice during the macropodoid radiation; once after the basal split between the Hypsiprymnodontidae and the remainder of the Macropodoidea, and a second time along the phylogenetic branch leading to modern-day potoroids. All sampled Macropodoidea appeared homozygous for the mAAV-EVE1 insertion. A ~0.3-kbp amplicon representing the uninterrupted pre-integration locus was recovered from all exemplars of the two non-macropodoid Diprotodontian suborders Phalangeriformes and Vombatiformes.

**Figure 2:**
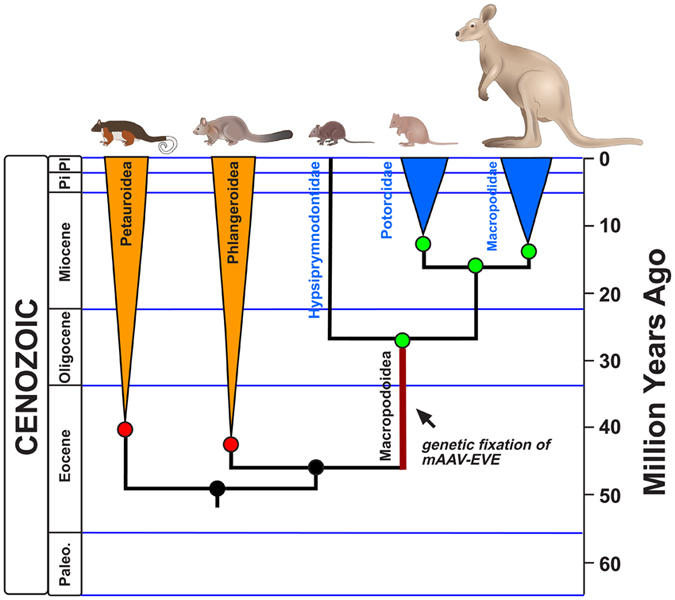
Temporal mapping of mAAV-EVE fixation. A time-scaled cladogram was constructed using previously published estimates of divergence among the Diprotodontia^28^. Green nodes represent inferred presence of endogenous mAAV sequences at the mAAV-EVE1 locus. Red nodes represent inferred absence of endogenous mAAV sequences. Node ages from left to right are 40.5 million years ago (MYA), 48.4 MYA, 42.8 MYA, 45.3 MYA, 26.8 MYA, 13.1 MYA, 15.9 MYA, and 13.8 MYA. Suborder Phalangeriformes, orange highlighted collapsed clades. Suborder Macropodiformes, blue highlight (Potoroidea and Macropodidae clades collapsed). Marsupial illustrations courtesy of Ethan Tyler, NIH Division of Medical Arts.

A time-scaled cladogram of the estimated dates of relevant marsupial speciation events^28^ shows the most recent common ancestor (MRCA) of the superfamilies Petauroidea, Phalangeroidea, and Macropodoidea to date to approximately 48.4 MYA, during the early Eocene (Fig. 2). Considering the phylogenetic distribution of mAAV-EVE1 among diprotodontians, we infer that infection, endogenisation, and genetic fixation of the exogenous marsupial AAV sequence that became mAAV-EVE1 occurred within a stem macropodoid population sometime after the Phalangeroidea-Macropodoidea split (45.3 MYA), but prior to the macropodoid radiation (27 MYA). The age of the ancient circulating marsupial virus could, of course, exceed the 45 MYA boundary.

### Maximum likelihood sequence reconstruction of an ancient AAV genome

**Figure 3:**
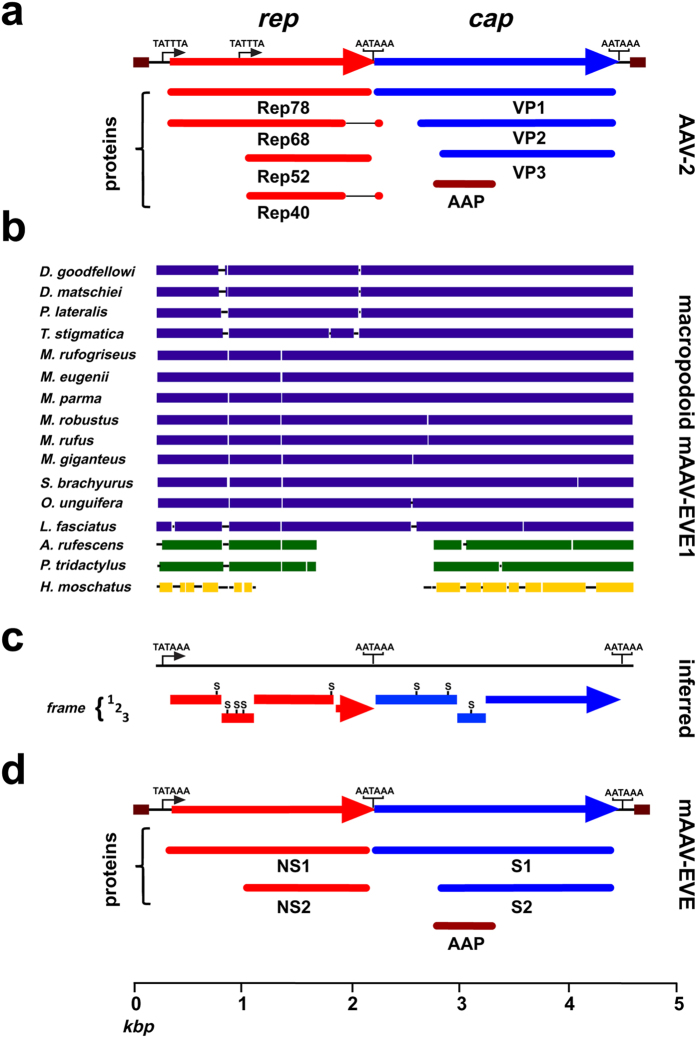
Maximum likelihood estimation of an inferred mAAV-EVE ancestral sequence. (**a**) Schematic representation of the genes and protein products encoded by the prototypical AAV serotype, AAV2. Relative positions of the P5 and P19 promoters (“TATA” boxes) and AATAAA polyadenylation signals are indicated. The central polyadenylation signal of AAV2 might not be utilised. (**b**) Schematic representation of the genetic structure of endogenous mAAV sequences from sixteen macropodoid species. Species names are indicated at the left. Macropodidae elements are in blue, Potoroidea elements are in green, and the Hypsiprymnodontidae element is in yellow. Coloured rectangles indicate areas of significant similarity compared with a multi-way alignment virtual composite sequence (90% similarity, window length = 50 bases). Gaps not bridged by a solid line represent deletions relative to the full-length mAAV-EVE1 consensus. (**c**) Raw, unedited maximum likelihood inference of the mAAV-EVE1 ancestral sequence. The *rep* gene is in red and the *cap* gene is in blue. Frameshifts are indicated by vertical discontinuities. Nonsense codons are represented by an “S”. (**d**) Schematic depiction of putative ancestral exogenous viral sequences prior to mAAV-EVE1 endogenisation, after editing for frameshifts, stop codons, and indels (Supplementary Table 1). NS1 and NS2, putative non-structural proteins; S1 and S2, putative structural proteins; AAP, putative assembly-activating protein.

A maximum likelihood algorithm, as implemented in MEGA^29^, was used to infer ancestral mAAV-EVE1 sequences from a nucleotide alignment of sixteen mAAV-EVE1 loci. The genetic structure of the orthologous mAAV-EVE1 sequences resembles that of contemporary AAVs (Fig. 3). The average size of the “full-length” mAAV-EVE1 virus-derived sequence was ~4.4 kbp. The representatives of the family Potoroidae (*A. rufescens* and *P. tridactylus*) bore internal deletions of ~1.2 kbp, whereas the EVE recovered from *H. moschatus* (the sole extant member of the family Hypsiprymnodontidae) displayed an internal deletion of 1.6 kbp. Additional minor indels were distributed throughout the various mAAV-EVE1 sequences. The average GC-content of full-length, virus-derived mAAV-EVE1 sequences is ~43%, compared to ~56%, 54%, and 46% for representative extant primate AAVs (serotypes 1 through 6), avian AAVs (strains DA1 and VR-865), and the goose/Muscovy duck parvoviruses, respectively. The majority of the nucleotide substitutions were singlets. The *rep* gene of the inferred ancestral mAAV-EVE1 genomic sequence contained three frameshift mutations and five nonsense codons (Fig. 3c). A heterogeneous region of repeated guanosine residues among the mAAV-EVE1 orthologs (resolved to glycine codons 146 and 147 of the inferred mAAV-EVE1 *rep* ORF) was recalcitrant to unambiguous alignment and was manually edited. The mAAV-EVE1 *cap* gene of the inferred genome contained three nonsense codons as well as two frameshift mutations (Fig. 3c). A putative TATA box ~90 bp upstream of the *rep* ORF and a putative polyadenylation signal (AATAAA) ~40 bp downstream of the *cap* ORF were identified in locations similar to those mapped in extant AAV genomes. A potential polyadenylation signal was also observed between the *rep* and *cap* genes of the mAAV-EVE1 sequences. A similarly located polyadenylation signal occurs within extant primate AAV genomes, and has been shown to be utilised in AAV5^30^. ITR sequences were not identified.

To ascertain potential binding sites for known transcription factors and to compare the structure of the mAAV-EVE1 NS1 (*i.e. rep*) promoter to that of an extant dependoparvovirus, the 216-nt sequence extending from the left-end of the mAAV-EVE1 genome to the start codon of the NS1 ORF and the equivalent 175-nt region of the AAV2 P5 promoter were analysed using the web-based software application TFBIND ( http://tfbind.hgc.jp)^31^. The algorithm identified ~300 transcription factor binding site (TFBS) motifs within each promoter (314 motifs within the AAV2 P5 promoter and 297 motifs within the mAAV-EVE NS1 promoter, each with some degree of binding site signature redundancy). The two promoters shared 77 of the TFBS signature motifs. Notably, putative binding sites for two factors shown to be important for the transcriptional regulation of the AAV2 P5 promoter, *viz.* YY1 and MLTF/USF^32^,^33^, occur at similar locations within each promoter. Similar to the AAV2 P5 promoter, a potential YY1 binding site was identified approximately 25 bp downstream of the putative TATA box of the mAAV-EVE1 NS1 promoter (although the upstream “−60” YY1 site was not identified). In addition, a potential binding site for MLTF/USF was identified approximately 60 bp upstream of the putative TATA box of the mAAV-EVE1 NS1 promoter, a location similar to the MLTF/USF site mapped approximately 50 bp upstream of the TATA box of the AAV2 P5 promoter^32^. Potential binding sites for equivalents of the AAV2 P19 and P40 promoters were not analysed owing to ambiguity as to the potential location of these gene-embedded promoters in the absence of transcript mapping data.

Equivalents of the major nonstructural, replication initiator protein (Rep78) and major coat protein (VP3) encoded by the prototypical AAV species (AAV2) were readily apparent (Fig. 3d). The existence of a methionine codon at an equivalent position to the AAV2 Rep52/40 ORF suggests that the exogenous ancestor of mAAV-EVE1 encoded at least one amino-terminally truncated Rep protein. A start codon at an equivalent position to the AAV2 VP1 protein suggests that the ancient exogenous virus also encoded a VP1-like molecule. An ACG codon at an equivalent position to that utilised by AAV2 for the translational initiation of the VP2 protein^34^ was not observed. This observation does not preclude, however, the possibility that the mAAV-EVE1 ancestor could have encoded a VP2-like polypeptide.

### Reconstructed ancient AAV reveals evolutionary retention of Rep and Cap protein structure

Modelling of the predicted ancestral mAAV-EVE1 major structural and non-structural proteins facilitated detailed comparisons with equivalent proteins from contemporary AAV counterparts. In addition to some notable differences, these comparisons revealed an astonishing degree of domain conservation for both classes of proteins over an evolutionary time span of tens of millions of years.

#### Rep protein

**Figure 4:**
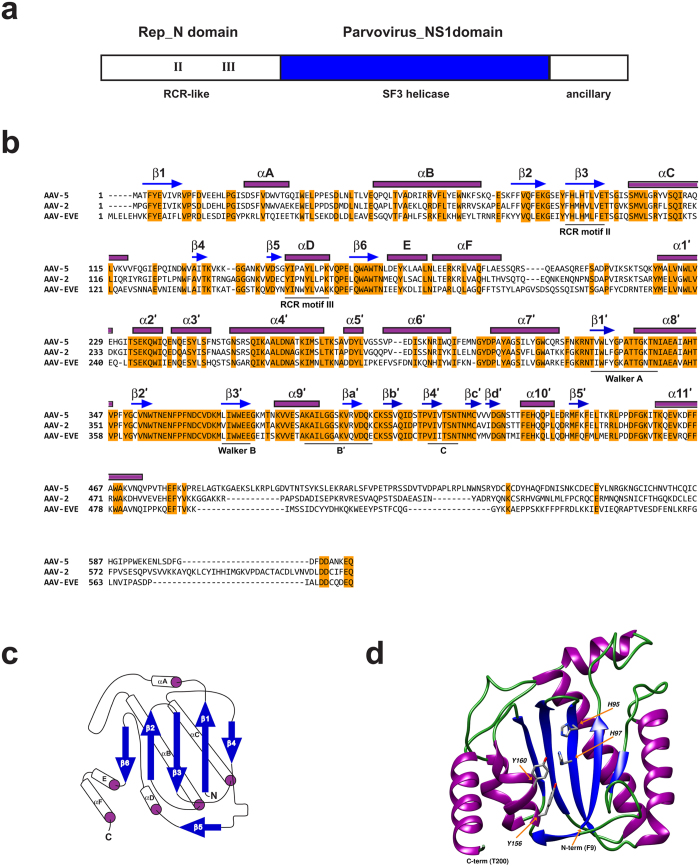
mAAV-EVE1 Rep protein. (**a**) Schematic representation of the mAAV-EVE1 Rep protein consisting of an amino-terminal nuclease domain, a central SF3 helicase domain, and a unique carboxy-terminal ancillary domain. (**b**) ClustalW alignment (most appropriate BLOSUM scoring matrix selected by ClustalW) of mAAV-EVE1 Rep with the Rep proteins encoded by AAV5 and AAV2 (ClustalW hosted by SIB Bioinformatics Research Portal ( www.expasy.org/genomics)). Putative beta strands are indicated by blue arrows. Putative alpha helices are indicated by purple rectangles. Nomenclature and approximate location of secondary structural features are from Hickman *et al.*^40^ and James *et al.*^94^ (“primed” secondary structure labels). The putative mAAV-EVE NS2 protein begins at amino acid residue 232. (**c**) Schematic representation of the mAAV-EVE1 Rep nuclease domain based upon comparison to the AAV5 Rep nuclease domain. (**d**) Molecular model of mAAV-EVE1 Rep nuclease domain (residues 9 through 200) based upon the structural determination of the nuclease domain of AAV5 Rep (QMEAN z-score = −0.11).

The overlapping polypeptides encoded by the AAV *rep* gene (Fig. 3a) are pleiotropic proteins shown to possess the nuclease and helicase activities required for initiation (and possibly termination) of AAV DNA replication, as well as packaging of nascent viral genomes^35^,^36^,^37^,^38^. Translation of the mAAV-EVE1 *rep* ORF yields an acidic 581 amino acid protein (estimated pI 5.2) with a predicted molecular weight of ~67.6 kilodaltons (kDa). A BLAST search of the NCBI non-redundant protein sequences database (nr), using the mAAV-EVE1 Rep protein as a query sequence, identified two conserved protein domains: i) an amino-terminal RepN superfamily catalytic domain associated with DNA binding and ssDNA endonuclease activity; and ii) a central parvovirus_NS1 superfamily domain associated with nucleoside triphosphate hydrolysis and helicase activity (Fig. 4a). The carboxy-terminal domain of mAAV-EVE1 Rep appears unique, with no significant similarity to known protein domains. The top four homologous Rep proteins identified by the BLAST search were encoded by bovine AAV (max score = 548), primate AAV5 (max score = 543), goat AAV-Go.1 (max score = 542), and avian AAV strain DA-1 (max score = 542). Amino acid alignment of AAV5, AAV2 and mAAV-EVE1 Rep proteins shows retention of clearly identifiable rolling circle replication (RCR) motifs II and III in the N-terminal nuclease domain of the mAAV-EVE1 Rep protein^39^ (Fig. 4b). Similar to extant dependoparvovirus Rep proteins, RCR motif I was not apparent. RCR motif II, known as the HUH motif, consists of two invariant histidine residues (positions 95 and 97 of the mAAV-EVE1 Rep protein) embedded within a patch of bulky hydrophobic amino acids (typically uHuHuuu, where u represents a hydrophobic residue). The primary sequence of the mAAV-EVE1 Rep nuclease domain was modelled on a template of AAV5 nuclease^40^ with a high degree of domain structure conservation. The AAV Rep nuclease domain fold consists of a five-stranded anti-parallel beta sheet bearing the conserved RCR motifs sandwiched between flanking alpha helical clusters (Fig. 4c). The modelled mAAV-EVE1 Rep nuclease is structurally similar to the extant AAV5 domain (QMEAN z-score −0.11), including the juxtaposition of the catalytic RCR motifs within the central cleft of the domain (Fig. 4c). Hickman *et al.*^40^ noted an acidic 38-amino acid loop between beta strand β1 and alpha helix αB that was a distinctive feature of the AAV5 nuclease domain (Fig. 4c). Although the two regions only share four aligned acidic residues, the relatively large excess negative charge of the loop region is conserved among the mAAV-EVE1 and AAV5 Rep proteins. The Superfamily 3 helicase domain fold (reviewed in Hickman and Dyda^41^), conserved among extant AAVs and other parvoviruses, was readily apparent within mAAV-EVE1 Rep residues 210 to 495, as were the conserved “Walker motifs” involved in nucleotide triphosphate binding and hydrolysis. The lysine residue of the highly conserved GKT triplet within the Walker A site was substituted with an asparagine residue in the inferred mAAV-EVE1 Rep protein. We were unable to find a precedent for this substitution in other Walker A site-containing nucleoside triphosphatases. Notably, amino acid substitutions at critical positions of known catalytic sites (*e.g.* the essential lysine residue of the Rep helicase Walker A site, the catalytic tyrosine residue of the DNA cleavage domain, and the invariant histidine residue of the VP1 phospholipase A2 domain) were observed in many of the individual mAAV-EVE1 Rep and Cap protein sequences. This pattern suggests that expression of active proteins from the endogenised viral sequence could be disadvantageous to host cell function and/or proliferation, resulting in selection of function-inactivating mutations.

#### Cap protein

**Figure 5:**
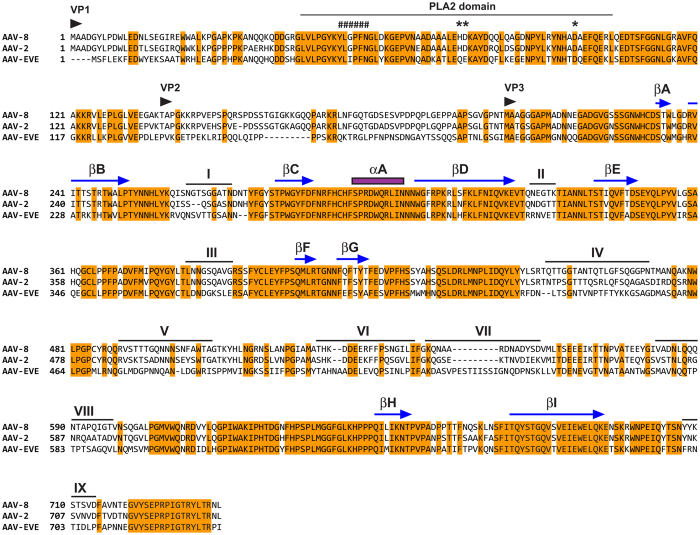
mAAV-EVE1 structural proteins. ClustalW alignment (most appropriate BLOSUM scoring matrix selected by ClustalW) of AAV8, AAV2, and mAAV-EVE1 VP1 coat proteins. Nomenclature and approximate location of secondary structural features are from Govindasamy *et al.*^90^, except for βA which is from Xie *et al.*^95^ Beta-strands are indicated by arrows. The position of the lone α-helix is indicated by a purple rectangle. PLA2, phospholipase A2 domain. Catalytic residues of the PLA2 domain are indicated by asterisks. Residues known to form a calcium-binding loop are indicated by hashtags.

The mAAV-EVE1 capsid gene encodes a VP1-like protein with a predicted molecular weight of 82.2 kDa. A phospholipase A2 domain required for parvovirus infectivity^42^ was conserved within mAAV-EVE1 VP1; however, the otherwise invariant histidine residue within the catalytic site of the domain was substituted with a glutamine in mAAV-EVE1 VP1 (Fig. 5). The VP3 equivalent of mAAV-EVE1 has a predicted molecular weight of 60.9 kDa. Analysis of the mAAV-EVE1 VP3 sequence using BLAST and sequence alignment with AAV2, AAV4, and AAV8 using ClustalW^43^ showed the highest sequence identity to AAV2 and AAV8 at ~61% compared to ~55% for AAV4. However, when the SWISS MODEL online subroutine^44^ was used to build a VP3 3D structure model for mAAV-EVE1 with AAV2 and AAV8 as reference templates, identities of 63.9% and 65.4%, respectively, were reported; accordingly, the AAV8-based model was used for further analysis.

**Figure 6:**
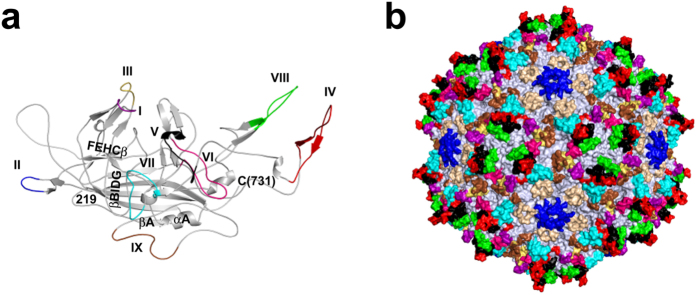
mAAV-EVE1 capsid structure. (**a**) VP3 monomer model of mAAV-EVE1. The nine variable regions (VRs) are colour-coded within a grey monomer and labelled. VR-I: purple, VR-II: blue, VR-III: yellow, VR-IV: red, VR-V: black, VR-VI: cerise pink, VR-VII: cyan, VR-VIII: green and VR-IX: brown. The core conserved secondary structure elements, the βBIDG and βCHEF β-sheets as well as αA, are labelled. The first N-terminal residue in the model (219) and C-terminal residue (731) are labelled. (**b**) The mAAV-EVE1 capsid with the VRs coloured as in (a). The HI loops are coloured in wheat. The juxtaposition of the VRs to the most prominent AAV capsid features, for example the 3-fold protrusions by VR-IV, VR-V, and VR-VIII, is evident in this image. The figures were generated using PyMOL^93^.

The VP3 monomer conserves the topology of the other available AAV structures. Salient features include β-strand A (βA), an eight-stranded β-barrel core (βBIDG-CHEF), and an α-helix (αA). Variable loop regions are dispersed among the secondary structure elements. When compared to the extant AAVs, the amino acids within the secondary structure elements are conserved while those within the apexes of the loops show the largest differences and are predicted to vary in structure (Fig. 6a). The placements of known regions of amino acid variability associated with surface loops (regions I through IX) are consistent with those of extant AAV capsids. These regions are located at or near the exterior surface of the VP3 monomer. Interestingly, VR-VII was substantially larger in mAAV-EVE1 compared to AAV2 and AAV8 (Fig. 5). The mAAV-EVE1 capsid, assembled from 60 copies of the VP3 common region of the VP, conserves the characteristic features of the AAVs: a depression at the icosahedral 2-fold axis, three protrusions surrounding an icosahedral 3-fold axis, a channel at the icosahedral 5-fold axis, and an HI loop (between βH and βI) lining a depression surrounding the 5-fold channel. The VRs cluster on the mAAV-EVE1 capsid surface to create local surface topology differences compared to other AAVs (Fig. 6b). For example, the larger VR-VII is located at the base of the 3-fold protrusions and extends into the depression surrounding the 5-fold channel, creating a unique surface topology in mAAV-EVE1. These VRs control several AAV functions, including receptor attachment, trafficking phenotypes, transduction efficiency, and antigenic reactivity^45^,^46^.

An AAG-initiated ORF encoding a potential AAP homolog, a protein involved in AAV capsid assembly^18^, was embedded within the *cap* gene (Fig. 3d). Assuming that, similar to numerous extant AAVs, translational initiation of the AAP ORF begins at a non-canonical CTG codon^47^,^48^, the putative mAAV-EVE AAP is 201 amino acids in length, with a molecular weight of ~21.6 kDa and a predicted pI of 8.30. The protein sequence demonstrates evolutionarily-conserved characteristic features of the AAP family, including a conserved core sequence flanked by hydrophobic and proline-rich regions respectively, followed by a region rich in serine/threonine residues^47^,^48^.

### Placement of mAAV-EVE1 within the *Dependoparvovirus* phylogeny

**Figure 7:**
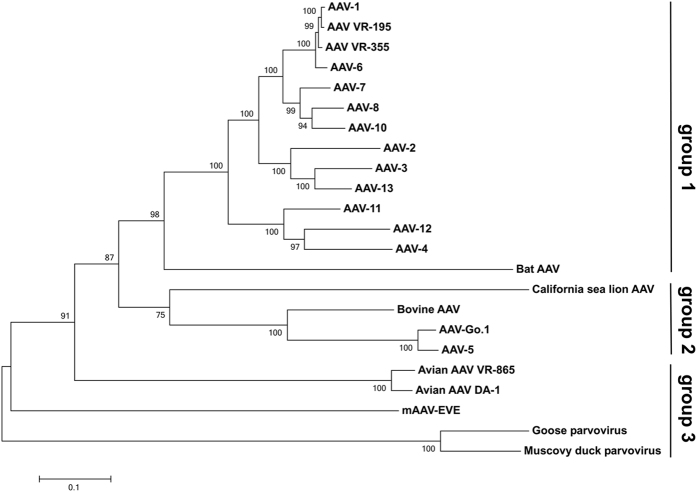
Phylogenetic analysis of viral genomic sequences. The phylogenetic relationship among the inferred mAAV-EVE1 genomic sequence and twenty-two extant dependoparvovirus genomes was analysed by the Maximum Likelihood method using a GTR + Γ substitution model. The tree bearing the highest log likelihood is shown (−40249.8723). Branch support values were derived by bootstrap analysis. Number of bootstraps = 500. A midpoint-rooted tree is shown. Scale = number of nucleotide substitutions per site.

To ascertain the phylogenetic relatedness of mAAV-EVE1 to extant dependoparvoviruses, a maximum likelihood phylogenetic tree was constructed using the reconstructed mAAV-EVE1 sequence along with genomic sequences from 22 extant members of the genus *Dependoparvovirus* (Fig. 7). This analysis placed the mAAV-EVE1 sequence near the base of the phylogenetic tree as a sister group to the non-autonomous dependoparvoviruses. The viral sequences fell into three groups: i) a diverse clade containing the primate AAVs with bat AAVs as sister taxa; ii) a clade containing AAVs infecting non-primate mammals (human serotype AAV5 and the highly homologous goat AAV, AAV-Go.1, most likely represent cross-species transmission among hosts^14^); and iii) a paraphyletic group containing mAAV-EVE1 along with avian dependoparvoviruses.

### Phylogenetic and mutation analysis of the mAAV-EVE1 locus

**Figure 8:**
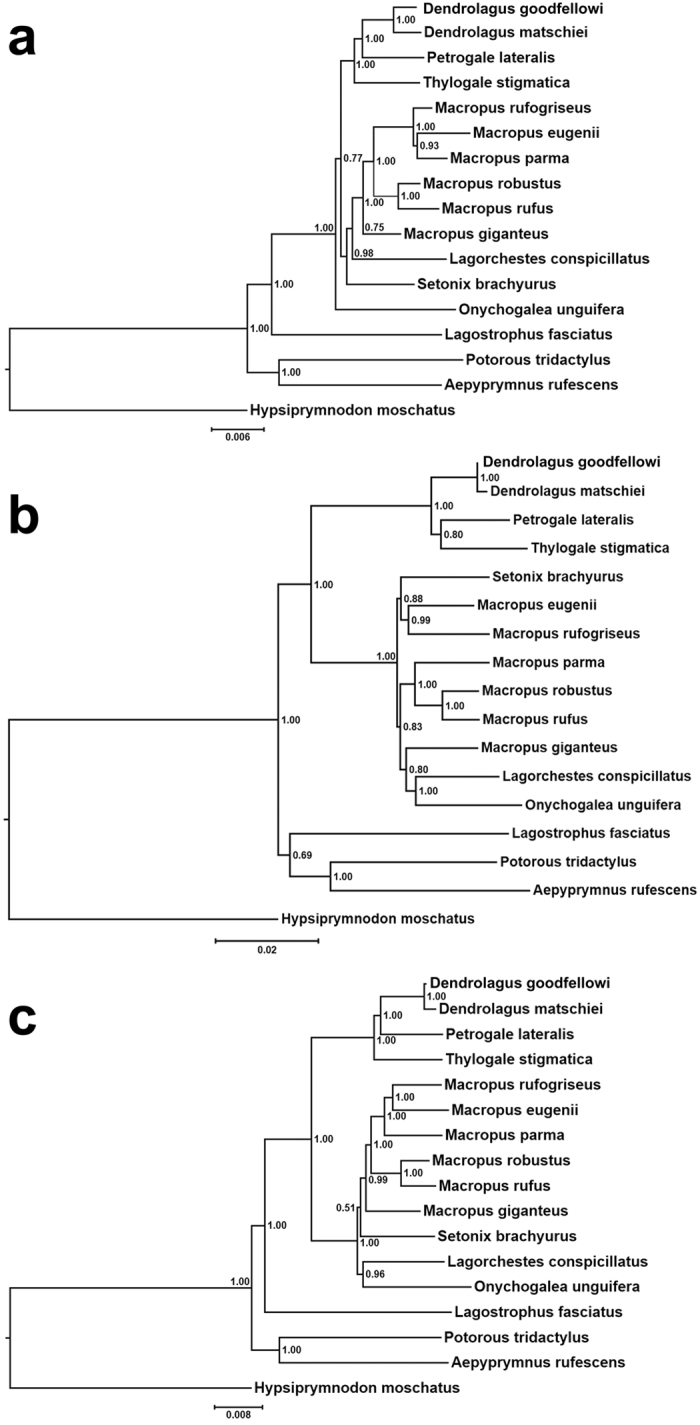
Macropodoid phylogeny based upon mAAV-EVE1 *versus* seven concatenated gene segments. (**a**) A maximum likelihood phylogenetic tree was constructed using concatenated nuclear gene segments representing exonic portions of the *ApoB*, *Brca1*, *IRBP*, *Rag1*, *vWF* , Protamine P1 and omega globin genes for the indicated species (see Meredith *et al.*^28^ for GenBank accession numbers; additional GenBank accession numbers for Protamine P1 and omega globin gene sequences are provided below). The tree was constructed using a partitioned analysis in which each gene was allowed its own model of sequence evolution as determined by Aikake Information Criteria and one thousand bootstrap pseudo-replicates. (**b**) Maximum likelihood tree constructed using the mAAV-EVE1 sequences from the indicated macropodoid species. The tree was constructed using a GTR + Γ + I substitution model and one thousand bootstrap pseudoreplicates. (**c**) Maximum likelihood phylogenetic tree for concatenated nuclear genes and mAAV-EVE1 sequences from the indicated macropodoid species. The tree was constructed using a partitioned analysis in which each gene was allowed its own model of sequence evolution as determined by Aikake Information Criteria and one thousand bootstrap pseudo-replicates. Scale = substitutions per site. GenBank accession numbers for the omega globin genes are: *P. tridactylus* - JX104646, *D. goodfellowi* - JQ042229, *M. eugenii* - AY014769, *M. giganteus* - AY014772, and *P. lateralis* - JQ042222, *T. stigmatica* - HQ283970. Genbank accession numbers for protamine P1 genes are: *H. moschatus* - AF187545, A. rufescens - AF187547, *P. tridactylus* - AF187548 , *D. goodfellowi* - AF187537, *L. fasciatus* - AY189936, *M. eugenii* - L35450, *M. giganteus* - L35333, *M. rufogriseus* - L35329, *M. rufus* - L35447, *O. unguifera* - AF187543, *S. brachyurus* - AF187541, *T. stigmatica* - AF187534.

The mAAV-EVE1 is a “fossil” insertion that was introduced into the MRCA of all Macropodiformes. Insertions occurring in a common ancestor are inherited by the offspring at the same orthologous position in the genome, and can therefore be used as shared derived characters to determine species relationships. The mAAV-EVE1 insertion event thus provides a homoplasy-free derived character that, analogous to SINE (Short Interspersed Nuclear Element) insertion, yields information regarding phylogenetic relationships among taxa^49^,^50^. In this instance, the common presence of mAAV-EVE1 supports monophyly of the Macropodoidea and, additionally, can serve as a genetic “barcode” for macropodoid species identification. Mitochondrial and nuclear gene sequences support the separation of the three macropodiform families Hypsiprymnodontidae, Potoroidae and Macropodidae and show the genetic distinctness of the banded hare-wallaby (*Lagostrophus fasciatus*, subfamily Sthenurinae) from all other extant members of the Macropodidae^51^,^52^. These findings are confirmed by analysis of the mAAV-EVE1 sequences, providing an independent way of resolving relationships within Macropodoidea (Fig. 8).

Like the well-resolved tree obtained for the concatenated nuclear genes (Fig. 8a), the tree based solely on mAAV-EVE1 sequences resolves the relationships within Macropodoidea (Fig. 8b). Although in Bayesian analysis *L. fasciatus* appears to be weakly resolved as sister to Potoroidae (0.69 Bayesian Posterior Probability [BPP]) rather than as sister to subfamily Macropodinae, both RaxML and PAUP* analyses resolved *Lagostrophus* as sister to all macropodines with moderate to strong support (95% bootstrap support RaxML, 95% PAUP*). Statistically, there is no support for a difference between the two alternative topologies in the mAAV-EVE1 tree under either parsimony (Kishino-Hasegawa [K-H] test *P* = 0.7631)^53^ or likelihood criteria (K-H test *P* = 0.39, Shimodaira-Hasegawa test *P* = 0.187)^54^.

Relationships within Macropodinae also appear to differ between trees based on concatenated nuclear genes and those based solely on mAAV-EVE1 sequences. Gatesy and Springer recently showed that combination of gene partitions in a concatenated dataset may reveal “hidden support” for tree nodes - “increased support for a clade in combined analysis of all data partitions relative to the support evident in separate analyses of the various data partitions^55^”. This principle holds for the mAAV-EVE1 data partition which, when concatenated with the nuclear gene data, results in a well-resolved tree for Macropodiformes under all three methods employed (Fig. 8c), with Potoroidae being sister to Macropodidae, Sthenurinae sister to Macropodinae, and strong resolution of all macropodine genera.

The rates of evolution of mAAV-EVE1 and the genomic flanking regions were compared. The mean pairwise distance for the mAAV-EVE1 sequences was more than twofold greater than that of the flanking regions (the mean distances of 0.073 and 0.035, respectively). The significance of this difference was tested using a sampling approach. A set of positions was arbitrarily selected from the alignment of AAVs such that the length and the nucleotide composition of these randomly generated alignments were not significantly different from the alignment of flanking regions (according to the χ^2^ test, *P* = 0.05). Analysis of 1,000 alignments constructed in this manner indicated that the mean distance of 0.035 and lower is highly unlikely (*P* = 0.002). Thus, the endogenised mAAV-EVE1 sequence evolved significantly faster than the flanking sequences. Notably, though, the downstream flanking region is highly conserved among marsupials and clearly identifiable even among several eutherians.

## Discussion

We identified an evolutionarily conserved locus that contains an AAV-derived EVE whose phylogenetic distribution among the Diprotodontia is restricted to macropodoid marsupials, indicative of an AAV genetic fixation event in the common ancestor of all Macropodiformes. Orthologous sequences were used to reconstruct the likely ORFs of the exogenous AAV that became integrated into the proto-macropodiform lineage. Given the paucity of ancient viral sequence information, this approach serves as a model for the generation of useful resources in the study of viral evolution. The predicted proteins encoded by the mAAV-EVE1 ORFs bear remarkable sequence and structural resemblance to contemporary AAV Cap, Rep and assembly-activating proteins, implying that AAVs have evolved within tight constraints imposed by both form and function. However, the catalytic sites of the endonuclease and helicase domains of the Rep proteins contain replacements of the key residues essential for activity, suggestive of selection for inactivation of the proteins encoded by integrated viral genes during macropodoid evolution.

The phylogenetic distribution of orthologous mAAV-EVE1 loci among the Diprotodontia suggests that genetic fixation of an exogenous AAV sequence occurred in the branch of the marsupial phylogeny that links the Macropodiformes to the remainder of the pouched mammals. The superfamilies Macropodoidea and Phalangeroidea are estimated to have diverged ~45 MYA, while the macropodoid radiation beginning with the basal divergence of the Hypsiprymnodontidae (represented by *H. moschatus*) from the lineage leading to the Potoroidea and Macropodidae has been dated to 26.8 MYA^28^,^51^,^56^,^57^. This defines an interval from the middle of the Eocene through the early Oligocene during which the exogenous marsupial AAV that became mAAV-EVE1 must have been actively circulating among susceptible host species. Although the exact temporal boundaries of the exogenous viral lineage are indeterminate, the origin of the exogenous marsupial AAV lineage must exceed the temporally proximal fixation boundary of 27 MYA and likely exceeds the distal fixation boundary of 45 MYA. Currently, no extant marsupial AAV strains have been described.

Other EVEs also provide evidence of the unexpected and remarkable persistence of viral lineages over millions of years. Katzourakis *et al.* determined that foamy viruses were endogenised within the genomes of members of the Pilosa (an ancient mammalian order within Xenarthra currently represented by two- and three-toed sloths) approximately 40 MYA^58^. Moreover, they concluded that foamy viruses have been circulating among susceptible mammalian hosts for at least 100 million years. Several groups of non-retroviral RNA viruses have also been determined to have an ancient origin^1^,^59^,^60^, which is in stark contrast to substitution rate-based estimates of the age of many RNA virus lineages^61^. Interestingly, the closest contemporary relatives of mAAV-EVE1 are the autonomously replicating dependoparvoviruses of migratory waterfowl, as well as other avian AAVs. This unexpected phylogenetic affinity suggests the possibility that the exogenous ancestor of mAAV-EVE1 could have been introduced to the island continent of Australia by migratory birds.

The genetic mechanism by which mAAV-EVE1 sequences became fixed within the Macropodiformes is an interesting question, and may have implications for macropodoid evolution. The data presented here support that at some point between 45 and 27 MYA, an exogenous AAV became integrated into the germline of an individual proto-macropod. Some of its F_1_ hybrid offspring, at this point heterozygous for mAAV-EVE1, would have inbred to produce F_2_ individuals that were homozygous for mAAV-EVE1. Such individuals became the ancestors of all extant Macropodiformes, which remain characterised by their mAAV-EVE1 homozygosity.

Under a neo-Darwinian model, the probability with which an allele will achieve genetic fixation within a population is proportional to its selection coefficient (a measure of its contribution to fitness)^62^. Under the assumption that the fixation of the mAAV-EVE1 allele within the lineage leading to the superfamily Macropodoidea is the result of evolutionary selective pressure, the endogenised mAAV viral sequences would have conferred a fitness advantage to their carriers. The mAAV-EVE1 *rep* and *cap* coding sequences within extant macropodoid species appear non-functional at the protein level due to the random genetic accumulation of indels, nonsense codons, and frameshifts, and are incapable of encoding full-length viral proteins. However, prior to mutational inactivation, the *rep* and *cap* genes of the mAAV-EVE1 locus may have expressed viral polypeptides for a significant period of macropodoid evolutionary history. The intracellular expression of endogenous, virus-derived polypeptides can potentially confer resistance to subsequent infection by related viruses (reviewed by Aswad and Katzourakis^63^). Although the majority of extant dependoparvoviruses are non-pathogenic, goose parvovirus and Muscovy duck parvovirus cause highly contagious and potentially fatal acute illness among susceptible avian hosts^64^. Both of these viruses are capable of autonomous replication in dividing tissue and show pronounced virulence in hatchlings. Molecular features associated with helper-independent replication and virulence of goose and Muscovy duck parvoviruses have not been defined, precluding an estimation of the degree of replication autonomy and virulence of the exogenous ancestor of the endogenised mAAV-EVE1 sequence. Nevertheless, it appears possible that endogenisation of the mAAV-EVE1 sequence into the genome of the MRCA of all Macropodoidea conferred inheritable resistance to infection by a potentially pathogenic mAAV-EVE1 ancestral virus, or mAAV-EVE1-related agents. Indeed, the mutagenic effect of endogenous retroviruses (ERVs) and other EVEs within the host genome and their potential influence on host evolutionary fitness is profound. ERVs and other EVEs can disrupt transcriptional units via insertional mutagenesis, act as a source of novel gene products usurped for cellular purposes (exaptation), foster chromosomal rearrangement, and alter host genetic regulatory networks and epistasis^65^,^66^,^67^,^68^.

An alternative possibility is that the phenotypic effect of the mAAV-EVE1 locus at the time of acquisition was effectively neutral, with a selection coefficient close to zero. The theoretical probability of fixation of a neutral allele within a population is inversely proportional to the effective population size (*i.e.*, 1/2*N*_*e*_ for diploid organisms, where *N*_*e*_ = effective population size), and the mean time to fixation of a neutral allele in a diploid population of constant size is directly proportional to four times the *N*_*e*_ (*i.e.*, 4*N*_*e*_ generations)^62^,^69^,^70^,^71^. The mean time to fixation can be substantially longer than 4*N*_*e*_ generations in expanding populations^72^. For large, randomly mating populations exhibiting a substantial degree of polymorphism, a significant reduction in effective population size, known as a “population bottleneck”, can greatly reduce both the overall heterozygosity (*i.e.*, allelic diversity) of the population as well as the mean time to fixation of a selected neutral allele. One biological means of rapid, precipitous population reduction is high-mortality infectious disease (particularly within a deme). Viral epizootics, for example, have been documented to cause mass mortality in susceptible host populations. The introduction of rinderpest virus, a morbillivirus, to Africa in the late 1800s caused the collapse of cattle populations in sub-Saharan Africa, as well as significant mortality among indigenous wildebeest populations^73^. An interesting hypothetical scenario is that fixation of mAAV-EVE1 sequences was facilitated by a population bottleneck within the proto-macropodiform lineage, caused by an epizootic of virulent mAAV or by an epizootic of its presumed helper virus (with mAAV as a non-pathogenic co-infecting satellite virus).

Endogenisation of many ancient viral lineages, including mAAV-EVE1 (this study), has been dated to around the time of the Eocene-Oligocene transition^4^,^58^,^74^. The time interval spanning the Eocene-Oligocene boundary was a period of significant global climate change and faunal extinction, and represents a period of marked northward drift of the Australian continent after its separation from Antarctica. This northward drift, accompanied by altered ocean circulation patterns, progressively led to climatic change with drying out of the northern and central parts of Australia^75^,^76^. The increasing replacement of rainforest habitats with open woodlands and savannahs in the Oligocene and later Miocene favoured radiation in the Macropodoidea to exploit them. Such ecological changes could conceivably have precipitated population bottlenecks even in the absence of any Australian panzootics. This scenario would not rule out the possibility that acquisition of mAAV-EVE1 in the proto-macropodiform lineage contributed to the conferment of an environmentally adaptive advantage, *e.g.* at the level of locomotion (which sets the Macropodiformes apart from other members of the Diprotodontia), to travel further in search of increasingly sparse sources of food.

The discovery of a plethora of EVEs derived from a broad spectrum of both RNA and DNA viruses within the genomes of numerous host species will allow researchers to examine increasing depths of ancient viral lineage. Age-matched reconstructions of lineage-associated endogenous viral genomes may serve as the input for subsequent rounds of ancestral state inference, thus “triangulating” ever older ancestral sequences. In the case of AAV, this potential well of ancient viral sequences may serve as a source of capsid variants for novel gene therapy vectors. This approach facilitates the reconstruction of far “older” AAV capsid sequences than putative ancestral AAV capsids deduced from observable genetic diversity amongst closely related contemporary AAV isolates, a strategy that has already spawned novel capsids with desirable vector properties^77^,^78^.

## Methods

### Sample acquisition

*Macropus giganteus* tissue samples were obtained in accordance with the provisions of a General Licence issued to the investigators by the NSW National Parks and Wildlife Service (NPWS, Licence number MWL000100088). The animals had been culled as part of the NSW Government Kangaroo Management Program and sample collection was exempt from Institutional Biosafety Committee and Animal Care and Ethics Committee approvals. All remaining samples were either collected from animals that had succumbed to road trauma, under the provision of a NPWS Scientific Licence held by the investigators (Licence number SL100022), or were available from previous studies in the form of purified genomic DNA or liver tissue samples.

### Cloning and sequencing of mAAV-EVE1 loci

For linker-mediated “genome walking” analysis, genomic DNA was extracted from kangaroo tissue samples (liver, muscle, and brain) using a FastPrep FP120 tissue homogeniser (ThermoSavant) utilising Lysing Matrix tubes (MP Biochemicals) according to the manufacturer’s instructions. Briefly, 50–75 mg of tissue was placed in a 2-ml tube containing MP Lysing Matrix A. To each tube, 360 μl of ATL buffer with proteinase K (DNeasy Blood & Tissue Kit; Qiagen) was added. Tissue was homogenised for 40 seconds at setting 5, followed by centrifugation at 10,000 × g for 2 minutes to collect fluid. Samples were incubated at 56 °C for 1 hour, and then centrifuged at 10,000 × g for 1 minute. Following centrifugation, 200 μl of the tissue homogenate was applied to a DNeasy column (Qiagen) and processed following the manufacturer’s instructions for animal tissue. To obtain endogenous AAV sequences, approximately 0.4 μg of genomic liver DNA was subjected to PCR amplification using Platinum Taq PCR SuperMix (Invitrogen) using combinations of previously reported primer pairs recognising conserved regions of the AAV genome (primers SIG+ and SIG−26 and primers AA55 and AA56^25^). The thermal cycling conditions were 94 °C for 5 minutes followed by 35 rounds of 94 °C for 30 seconds, 55 °C for 30 seconds, and 72 °C for 30 seconds, with a final 5 minute extension at 72 °C. A positive PCR result was obtained from liver DNA using the following primer pair:

“AA55” 5′-GTGCCCTTCTACGGCTGCGTCAACTGGACCAATGAGAACTTTCC-3′ and “SIG−” 5′-GAATCCCCAGTTGTTGTTGATGAGTC-3′. Upon identification of an endogenous AAV “anchor sequence”, linker-mediated “genome walking” was performed using the GenomeWalker Universal Kit (Clontech) according to the manufacturer’s instructions. Briefly, kangaroo liver DNA (2.5 μg) was digested overnight in separate 100-μl reactions containing individual restriction endonucleases (e.g., *Dra*I, *Nru*I, *Sca*I, or *Stu*I) to yield blunt-ended genomic DNA fragments. The restriction enzyme digest was then heat inactivated at 70 °C for 10 minutes, and the genomic DNA fragments were partially purified using a PCR Kleen Spin column (BioRad). A GenomeWalker kit-provided adapter was ligated to enzyme-digested genomic DNA fragments overnight at 16 °C using T4 DNA ligase. Following heat inactivation (70 °C for 10 minutes), residual adapters were removed by passage over a PCR Kleen Spin column (BioRad) according to manufacturer’s instructions. Following nested PCR using unique sequence/adapter-specific primers pairs, amplified PCR products were “TA-cloned” into pCR4-TOPO (Invitrogen). Following bacterial transformation and antibiotic marker selection, individual colonies were expanded in small-scale liquid culture, and plasmid DNA was isolated for sequencing using a QIAprep Spin Kit (Qiagen).

Genomic DNA for amplification across the mAAV-EVE1 locus was extracted from liver tissue samples using either a Gentra Puregene Tissue Kit (Qiagen) or a Blood & Cell Culture DNA Kit with Genomic-tip 100/G (Qiagen), in each case following the manufacturer’s protocols for DNA extraction from tissues. Amplification of the locus containing mAAV-EVE1 was initially achieved using a forward primer (AAV-EVE_flank_up, targeting the upstream flanking region: 5′-GATGTTTACAGATTAGTRTTKYATCATCAGTGCTATTTYCYCWCAAWRARRATYCC-3′) containing multiple degenerate positions to accommodate phylogenetically diverse marsupials, and a reverse primer (AAV-EVE_flank_dwn, targeting the downstream flanking region: 5′-AGGGAGAGTACCTATTATCTTAATTACTGTCAGACC-3′). The forward primer includes a 5′ non-homologous tail to facilitate reamplification. These primers amplified the locus (irrespective of its mAAV-EVE1 occupancy) from all sampled marsupials. Later, some macropodiform mAAV-EVE1 loci were amplified using a forward primer without degenerate positions (Macr(−335)flank_up: 5′-CCTGGAATTTGTGGGTGGAAACAATGATCC-3′), specifically targeted to Macropodiformes. Amplifications were carried out using the Expand Long Template PCR System (Roche) or a LongRange PCR Kit (Qiagen) according to the manufacturers’ instructions. Amplicons were gel-extracted using a Wizard SV Gel and PCR Clean-Up System (Promega) and cloned using the TOPO TA Cloning Kit for Sequencing, the Zero Blunt TOPO PCR Cloning Kit for Sequencing (Life Technologies; both in conjunction with One Shot TOP10 Chemically Competent *E. coli* cells), or the pGEM-T Easy Vector System I (Promega; in conjunction with XL10-Gold Ultracompetent Cells [Agilent]). Sanger sequencing of cloned inserts utilised primers directed against the cloning vectors, as well as internal, amplicon-specific primers, using an AB 3730xl instrument (Australian Genome Research Facility). A portion of the cloned mAAV-EVE1 sequences and “empty loci” were amplified using Platinum PCR SuperMix High Fidelity (Invitrogen) in conjunction with primers AAV-EVE_flank_up and AAV-EVE_flank_dwn. Amplified sequences were cloned by direct addition of a portion of the final PCR to topo-activated pCR4-TOPO (Invitrogen) without prior gel purification. Cloned fragments were transformed into bacterial strain DH10B (Invitrogen) by electroporation following desalting in a BioRad PCR Kleen Spin column according to manufacturer’s instructions. The cloned amplicons were sequenced at the U.S. Food and Drug Administration (FDA) Bethesda campus core facility, or by commercial vendor.

### Reconstruction of ancestral mAAV-EVE1 sequence

A maximum likelihood algorithm, as implemented in MEGA6.06^79^, was used to infer ancestral mAAV-EVE1 nucleotide sequences from a multiple sequence alignment of sixteen macropodoid mAAV-EVE1 loci (*M. robustus, M. rufus, M. parma, M. giganteus, M. eugenii, M. rufogriseus, O. unguifea, S. brachyurus, D, goodfellowi, D. matschiei, P. lateralis, T. stigmatica, L. fasciatus, A. rufescens, P. tridactylus, and H. moschatus*), with the inclusion of “empty” mAAV-EVE1 loci from related non-macropodoid marsupials (*P. breviceps*, *P. peregrinus*, *S. maculatus*, *T. vulpecula*, *L. latifrons*, *P. cinereus*, *D. marsupialis*, and *M. domestica*) serving as an outgroup (Fig. S1). The relevant sequences determined as part of this study have been assigned GenBank accession numbers KX239848-KX239872. Briefly, the twenty-four member dataset was aligned using the MUSCLE multiple sequence alignment algorithm^80^ with default settings (gap open penalty = −400; gap extend penalty = 0; clustering method (all iterations) = UPGMB; minimum diagonal length (lambda) = 24). An isolated alignment of eight outgroup nucleotides (IUPAC nucleotide code: KGRTHACY) extant within the “empty locus” sequences (most likely representing nucleotides lost from the stem-macropodoid locus during the exogenous AAV integration event) was removed from the alignment. A short heterogeneous region of predominantly reiterated guanosine residues occurring within the 5′ portion of the mAAV-EVE *rep* gene was manually aligned. The most appropriate nucleotide substitution model was determined using the “Find Best DNA/Protein Models” function in MEGA, which determines the maximum likelihood fits of twenty-four evolutionary models given the data. For the mAAV-EVE1 dataset, the Tamura 3-parameter model with heterogeneity of substitution rates among sites modelled via a discrete Gamma distribution with five rate categories (*i.e.*, T92 + G) gave the lowest Bayesian Information Criterion score (33153.754), and was chosen as the best nucleotide substitution model for further analysis. An mAAV-EVE1 evolutionary tree was constructed in MEGA using the maximum likelihood (ML) method (substitution model = T92 + G; gaps/missing data treatment = use all sites; ML heuristic method = nearest-neighbor interchange; initial tree for ML inference was generated automatically by maximum parsimony analysis). Most probable ancestral sequences at each node of the ML tree were exported as a “Detailed Text Export” file from the MEGA6 Tree Explorer module. The most probable ancestral sequences were extracted from the Detailed Text Export file using the command-line utility program, ExtAncSeqMEGA.exe^81^. Due to genomic sequence deletions within mAAV-EVE1 loci occurring within the macropodoid basal taxon, *H. moschatus,* as well as the potoroids (*A. rufescens* and *P. tridactylus*), the full-length inferred mAAV-EVE1 sequence used for ancestral AAV modelling was derived from node 39 of the mAAV-EVE1 evolutionary tree (Fig. S2), occurring at the split between *Lagostrophus fasciatus* and the remainder of the Macropodidae at approximately 13.8 MYA. Reading frames encoding the *rep* and *cap* genes within the inferred ancestral sequence were identified by a BLAST^27^ search of translated nucleotide databases (tblastx) for significant homology to extant AAV proteins using an ancestral mAAV-EVE1 query sequence. Using homology among the translated mAAV-EVE1 ORFs and extant dependoparvovirus protein sequence alignments as a guide, the raw mAAV-EVE1 ancestral nucleotide sequence was manually edited for frameshifts, nonsense codons, and indels. In all but one instance, frameshifts within the “raw” inferred mAAV-EVE1 nucleotide sequence could be resolved by correction based upon a non-frameshifted member of the dataset. A frameshift occurring within mAAV-EVE1 *rep* codon 242 was corrected by arbitrary insertion of a dinucleotide sequence (TT). At various positions, the inferred mAAV-EVE1 ancestral sequence (Node 39 sequence; Fig. S3) was manually edited to give precedence to nucleotides encoding amino acid residues among one or more mAAV-EVE1 sequences homologous with highly conserved extant AAV protein residues (Table S1).

### Datasets, BLAST searches, phylogenetic analyses

Protein and nucleotide sequences were downloaded from the NCBI and ENSEMBL websites. Similarity searches were performed using the non-redundant protein sequence database at the NCBI and the BLAST program. Multiple nucleotide and protein sequence alignments were constructed using the MUSCLE program^80^ and then adjusted manually. For data analysis, phylogenetic trees based on multiple alignments were constructed using the maximum-likelihood, neighbor-joining, minimum-economy and maximum-parsimony methods as implemented in MEGA^29^ and FASTREE 2^82^. An optimal model of substitutions for phylogenetic reconstructions was chosen using the MEGA5 program. Viewing the mAAV-EVE1 locus as a macropodoid gene, a maximum likelihood phylogenetic tree was constructed using mAAV-EVE1 sequence data from sixteen macropodoid species and compared to a maximum likelihood tree of the same species constructed using alignment of a set of seven concatenated exonic gene segments (*ApoB*, *BRCA1*, *IRBP*, *Rag1*, *vWF,* Protamine P1 and omega globin genes) available in GenBank^28^,^57^,^83^.

Phylogenetic analyses were carried out on the concatenated data set using maximum likelihood methodologies implemented in RAxML v7.2.8^84^ and PAUP* 4.0b10^85^ and Bayesian inference as implemented in MrBayes 3.2^86^ with the data treated either as a single unpartitioned block or partitioned by gene (and mAAV-EVE1). This approach allowed each gene partition to have its own model of sequence evolution as determined by the Akaike Information Criterion in jModeltest^87^. The GTR + Γ + I model was chosen for the unpartitioned data. Node support was estimated by 1,000 bootstrap pseudo-replications for RAxML and PAUP*. Bayesian analyses utilised random starting trees and two simultaneous runs of four Markov chains (one cold and three heated using default heating values) applied for 5 × 10^6^ generations with sampling every 1,000^th^ generation. The first 1.25 × 10^6^ generations were discarded from each run as burn-in. The remaining trees were used to construct a majority-rule consensus with posterior probabilities >0.95 deemed as strong support^88^. We evaluated the fit of our data to alternative phylogenetic relationships using the SH^54^ and KH^53^ tests implemented in PAUP* 4.0b10^85^.

### Building a 3D structure model for mAAV-EVE1

The inferred mAAV-EVE1 VP3 sequence was used to generate a 3D structure model with the AAV8 VP3 structure coordinates (RCSB PDB accession No. 2QA0) supplied as a reference template to the SWISS MODEL online 3D modelling server ( http://swissmodel.expasy.org/)^44^. A comparison of the mAAV-EVE1 model to the AAV4 VP3 structure (RCSB PDB accession No. 2G8G) to identify VRs was conducted using the secondary structure matching (SSM) subroutine within PDBeFOld ( http://www.ebi.ac.uk/msd-srv/ssm/)^89^. VP3 VRs were defined as stretches of two or more sequential Cα positions that are >1 Å apart (as previously defined in Govindasamy *et al.*^90^). The structures were visualised in the COOT program for further comparison of the VRs between mAAV-EVE1, AAV2, AAV4, and AAV8^91^. To enable description of the assembled mAAV-EVE1 capsid, the VP3 monomer model was used to generate a 60mer by icosahedral matrix multiplication in the Viperdb online server ( http://viperdb.scripps.edu/oligomer_multi.php)^92^. The VP3 and 60mer coordinates were used to generate secondary structure and capsid surface images, respectively, using the PyMol program^93^.

## Additional Information

**How to cite this article**: Smith, R. H. *et al.* Germline viral “fossils” guide *in silico* reconstruction of a mid-Cenozoic era marsupial adeno-associated virus. *Sci. Rep.* **6**, 28965; doi: 10.1038/srep28965 (2016).

## Supplementary Material

Supplementary Information
