# Supplementary material for: Germline viral “fossils” guide in silico reconstruction of a mid-Cenozoic era marsupial adeno-associated virus
Source: Sci Rep. 2016 Jul 5;6:28965. doi: 10.1038/srep28965 (PMC4932596; doi:10.1038/srep28965)
Supplement: Supplementary Information [file srep28965-s1.pdf]

Germline viral "fossils" guide *in silico* reconstruction of a mid-Cenozoic era marsupial adeno-associated virus

Richard H. Smith, Claus V. Hallwirth, Michael Westerman, Nicola A. Hetherington, Yu-Shan Tseng, Sylvain Cecchini, Tamas Virag, Mona-Larissa Ziegler, Igor B. Rogozin, Eugene V. Koonin, Mavis Agbandje-McKenna, Robert M. Kotin and Ian E. Alexander

```
                          1                                                50
Aepyprymnus_rufescens     AAATCCTAGA TTATGGGAGT GAGCAAGCAA AGAAGTGGCT ACACTCTCCA
Dendrolagus_goodfellowi   AAATCCTAGA TTATGAGAGC CAGCGATCAA AGAAGTGGCC ACATTCTCCT
Dendrolagus_matschiei     AAATCCTAGA TTATGAGAGC CAGCGATCAA AGAAGTGGCC ACATTCTCCT
Hypsiprymnodon_moschatus  AAATCCTACA TTATGAGAGC AAGCGGGCAA AGGAGTGGCC ACACTCTCCA
Lagostrophus_fasciatus    .AATCTTAGA TTATGAGAGC CAGGGATCAA AGAAGTGGCC ACACTCTCCA
Macropus_eugenii          AAATCCTAGA TTATAAGAGC CAGTGATCAA AGAAGTGGCC ACACTCTCCT
Macropus_parma            AAATCCTAGA TTATGAGAGC CAGAGATCAA AGAAGTGGCC ACACTCTCCT
Macropus_robustus         AAATCCTAGA TTATGAGAGC CAGCGATCAA AGAAGTGGCC ACACTCTCCT
Macropus_rufogriseus      AAATCCTAGA TTGTGAGAGC CAGCGATCAA AGAAGTGGCC ACACTCTCCT
Macropus_rufus            AAATCTTAGA TTACGAGAGC CAGCGATCAA AGAAGTGGCC ACACTCTCCT
Macropus_giganteus        AAATCCTAGA TTATGAGAGC CAGCGATCAA AGAAGTGGCC ACACTCTCCT
Onychogalea_unguifera     AAATCCTAGA TTATGAGAGC CAGTGATCAA AGAAGTRGCC ACACTCTCCT
Petrogale_lateralis       AAATCCTAGA TTGTGAGAGC CAGCGAACAA AGAAGTGGCC ACATTCTCCT
Potorous_tridactylus      AAATCCTAGA TTATGGGAGA GAGGGAGCAA AGAAGTGGCC ACACTCTCCA
Setonix_brachyurus        AAATCCTAGA TTATGAGAGC CAGCGATCAA AGAAGTGGCC ACACTCTCCT
Thylogale_stigmatica      AAATCCTAGA TTGTGAGAGC CAGCGATCAA AGAAGTGGCC ACATTCTCCT
Didelphis_marsupialis     AGATCCTGGA TTTTG..... .......... .......... ..........
Lasiorhinus_latifrons     AAATCATGGA TTATG..... .......... .......... ..........
Petaurus_breviceps        .AATCCTGGA TTATG..... .......... .......... ..........
Phascolarctos_cinereus    AAATCATAGA TTATG..... .......... .......... ..........
Pseudocheirus_peregrinus  AAATCCTGGA TTATG..... .......... .......... ..........
Spilocuscus_maculatus     AAATCCTGGA TTCTG..... .......... .......... ..........
Trichosurus_vulpecula     AAATCCTGGA TTATG..... .......... .......... ..........
Monodelphis_domestica     CCATCATGGA TTACA..... .......... .......... ..........

                          51                                              100
Aepyprymnus_rufescens     TAAGATTTGA AAAGCCC... .AAGCAGATG ATGTTAATTA CCCATAATGC
Dendrolagus_goodfellowi   TAAGATTTGA AAAGCCCACC AAAGCAGATG ATGT.AATTA CCCATAATGC
Dendrolagus_matschiei     TAAGATTTGA AAAGCCCACC AAAGCAGATG ATGT.AATTA CCCATAATGC
Hypsiprymnodon_moschatus  TTAGATTTGA AAAGCCCGCC AAAGCGGATG ACGT.AACTA CTCAGAATGC
Lagostrophus_fasciatus    TAAGATTTGA AAAGCCTGCC AAAGCAGATG ATGT.AATTA CCCATAATGC
Macropus_eugenii          TAAGATTTGA AAAGCCCACC AAAGCAGATG ATGT.AATTA CCCATAATGC
Macropus_parma            TAAGATTTGA AAAGCCCACC AAAGCAGATG ATGT.AATTA CCCATAATGC
Macropus_robustus         TAAGATGTGA AAAGCCCACC AAAGCAGATG ATGT.AATTA CCCATGATGC
Macropus_rufogriseus      TAAGATTTGA AAAGCCCACC AAAGCAGATG ACGT.AATTA CCCATAATGC
Macropus_rufus            TAAGATTTGA AAAGCCCACC AAAGCAGATG ATGT.AATTA CCCATGATGC
Macropus_giganteus        TAAGATTTGA AAAGCCCACC AAAGCAGATG ATGT.AATTA CCCATAATGC
Onychogalea_unguifera     TAAGATTTGA AAAGCCCACC AAAGCAGATG ATGT.AATTA CCCATAATAC
Petrogale_lateralis       TAAGATTTGA AAAGCCCACC AAAGCAGATG ATGT.AATTA CCCATAATGC
Potorous_tridactylus      TAAAATTTGA AAAGCCCGCC AAAGCAGATG ATGT.AATTA CCCATAATGC
Setonix_brachyurus        TAAGATTTGA AAAGCCCACC AAAGCAGATG ATGC.AATTA CCCATAATGC
Thylogale_stigmatica      TAAGATTTGA AAAGCCCACC AAAGCAGATG ATGT.AATTA CCCATAATGC
Didelphis_marsupialis     .......... .......... .......... .......... ..........
Lasiorhinus_latifrons     .......... .......... .......... .......... ..........
Petaurus_breviceps        .......... .......... .......... .......... ..........
Phascolarctos_cinereus    .......... .......... .......... .......... ..........
Pseudocheirus_peregrinus  .......... .......... .......... .......... ..........
Spilocuscus_maculatus     .......... .......... .......... .......... ..........
Trichosurus_vulpecula     .......... .......... .......... .......... ..........
Monodelphis_domestica     .......... .......... .......... .......... ..........

                          101                                             150
Aepyprymnus_rufescens     AATTAGAATC AGTCCTAGAC TGAGTT..GC AAGAGGCTAT AAAAAGAAGG
Dendrolagus_goodfellowi   AATTGGAATC AGTCCCAGAC TGCATT..GC AAGAGGCTAT AAAAAGAAGG
Dendrolagus_matschiei     AATTGGAATC AGTCCCAGAC TGCATT..GC AAGAGGCTAT AAAAAGAAGG
```

```
Hypsiprymnodon_moschatus AATTGAAATC AGTCCCAGAC TGCATTGCGC AAGAAGCTAT AAAAAGAAAG
Lagostrophus_fasciatus   AATTGGAATC AGTCCCAGAC TGCATT..GC AAGAGGTTAC AAAAAGAAGA
Macropus_eugenii         AATTGGAATC AGTCCCAGAC TGCATT..GC AAGAGGCTAT AAAAAGAAGG
Macropus_parma           AATTGGAATC AGTCCCAGAC TGCATT..GC AAGAGGCTAT AAAAAGAAGG
Macropus_robustus        AATTGGAATC AGTCCCAGAC TGCATT..GC AAGAGGCTAT AAAATGAAGG
Macropus_rufogriseus     AATTGGAATC AGTCCCAGAC TGCATT..GC AAGAGGCTAT AAAAAGAAGG
Macropus_rufus           AATTGGAATC AGTCCCAGAC TGCATT..GC AAGAGGCTAT AAAATGAAGG
Macropus_giganteus       AATTGGAATC AGTCCCAGAC TGCATT..GC AAGAGGCTAT AAAAAGTAGG
Onychogalea_unguifera    AATTGGAATC AGTCCCAGAT TGCATT..GC AAGAGGCTAT AAAAAGAAGG
Petrogale_lateralis      AATTGGAATC AGTCCCAGAC TGCATT..GC AAGAGGCTAT AAAAAGAAGG
Potorous_tridactylus     AATTGGAATC AGTCCCAGAC TGTGTT..GT AAGAGGCTAT AAAAAGAAGG
Setonix_brachyurus       AATTGGAATC AGTCCCAGGC TGCATT..GC AAGAAGCTAT AAAAAGAAGG
Thylogale_stigmatica     AATTGGAATC AGTTCCAGAC TGCATT..GC AAGAGGCTAC AAAAAGAAGG
Didelphis_marsupialis    .......... .......... .......... .......... ..........
Lasiorhinus_latifrons    .......... .......... .......... .......... ..........
Petaurus_breviceps       .......... .......... .......... .......... ..........
Phascolarctos_cinereus   .......... .......... .......... .......... ..........
Pseudocheirus_peregrinus .......... .......... .......... .......... ..........
Spilocuscus_maculatus    .......... .......... .......... .......... ..........
Trichosurus_vulpecula    .......... .......... .......... .......... ..........
Monodelphis_domestica    .......... .......... .......... .......... ..........

                         151                                                200
Aepyprymnus_rufescens    GTGTATGCCT AGAAGTTCAT T.ATCATCTA CCAACTGAGC TAGTGAGCAT
Dendrolagus_goodfellowi  ATGTGTGCCT AGAAATTCAT TAATCATCTA CCGACTGAGC GAGTGTGCAT
Dendrolagus_matschiei    ATGTGTGCCT AGAAATTCAT TAATCATCTA CCGACTGAGC GAGTGTGCAT
Hypsiprymnodon_moschatus GCATACGCCA AGAAATTCAT TAATCATCTA CCAACCAAGC GAGTGAGCGT
Lagostrophus_fasciatus   GTGTATGACT AGAAATGCAT TAATCACCCA CTGACTGAGC AAGTATGCAT
Macropus_eugenii         ATGTGTGCCT AGAAATTCAT TAATCATCTA CTGACTGAGC GAGTGTGCAT
Macropus_parma           ATGTGTGCCT AGAAATTCAT TAATCATCTA CCGACTGAGC GAGTGTGCAT
Macropus_robustus        ATGTGTGCCT AGAAATTCAT TAATCATCTA CCGACTGAGT GAGTGTGCAT
Macropus_rufogriseus     ATGTGTGCCT AGAAATTCAT TAATCATCTA CTGACTGAGC GAGTGTGCAT
Macropus_rufus           ATGTGTGCCT AGAAATTCAT TAATCATCTA CCGACCGAGT GAGTGTGCAT
Macropus_giganteus       ATGTGTGCCT AGAAATTCAT TAATCACCTA CCGACTGAGC GAGTGTGCAT
Onychogalea_unguifera    ATGTGTGCCT AGAAATTCAT TAATCATCTA CCGACTGAGC GAGTGTGCAT
Petrogale_lateralis      ATGTGTGCCT AGAAATTCAT TAATCATCTA CCAACTGAGC GAGTGTGCAT
Potorous_tridactylus     GTGTATGCCT AAAAATTCAT TAATCATCTA CCAACTGAGC TAGTGAGCAT
Setonix_brachyurus       ATGTGTGCCT AGAAATTCAT CAATCATCTA CCGACTGAGC GAGTGTGCAT
Thylogale_stigmatica     ATGTGTGCCT AAAAATTCAT TAATCATCTA CCGACTGAGC GAGTGTGCAT
Didelphis_marsupialis    .......... .......... .......... .......... ..........
Lasiorhinus_latifrons    .......... .......... .......... .......... ..........
Petaurus_breviceps       .......... .......... .......... .......... ..........
Phascolarctos_cinereus   .......... .......... .......... .......... ..........
Pseudocheirus_peregrinus .......... .......... .......... .......... ..........
Spilocuscus_maculatus    .......... .......... .......... .......... ..........
Trichosurus_vulpecula    .......... .......... .......... .......... ..........
Monodelphis_domestica    .......... .......... .......... .......... ..........

                         201                                                250
Aepyprymnus_rufescens    CAAAGGAAGA AGAATAAATA GAAGAGATGA AGACATGCTG GAGCTGGAAC
Dendrolagus_goodfellowi  CAAAAGAAGA AGAATAAATA GAAGAGATGA AGACATGCTG GAGCTGGAAC
Dendrolagus_matschiei    CAAAAGAAGA AGAATAAATA GAAGAGATGA AGACATGCTG GAGCTGGAAC
Hypsiprymnodon_moschatus CAAAAAAAGA AGAATAAATA T..GAGATGA AGATATGCCG GAGCTGAAAC
Lagostrophus_fasciatus   CAAAAGAAGA AGACTAAATA GAAGAGATGA AGACATGCTG GAGCTGGAAC
Macropus_eugenii         CAAAAGAAGA AGAATAAATA GAAGAGATGA AGACATGCTG GAGCTGGAAC
Macropus_parma           CAAAAGAAGA AGAATAAATA GAAGAGATGA AGACATGCAG GAGCTGGAAC
Macropus_robustus        CAAAAGAAGA AGAATAAATA GTAGAGATGA AGACATGCTG GAGCTGGTAC
Macropus_rufogriseus     CAAAAGAAGA AGAATAAATA GAAGAGATGA AGACATGCTG GAGCTAGAAC
Macropus_rufus           CAAAAGAAGA AGAATAAATA GTAGAGATGA AGACATGCTG GAGCTGGAAC
Macropus_giganteus       CAAAAGAAGA AGAATAAATA GAAGAGATGA AGACATGCTG GAGCTGGAAC
Onychogalea_unguifera    CAAAAGAAGA AGAATAAATA GAAGAAATGA AGACATGCTG GAGCTGGAAC
Petrogale_lateralis      CAAAAGAAGA AGAATAAATA GAAGAGATGA AGACACGCTG GAGCTGGAAC
Potorous_tridactylus     CAAAAGAAGA AGAATAAATA AAAGAGATGA AGACATGCTG GAACTGGAAC
```

```
Setonix_brachyurus        CCAAAGAARA ARAATAAATA GAAGAGATGA AGACATGCTG GAGCTGGAAT
Thylogale_stigmatica      CAAAAGAAGA AGAATAAATA GAAGAGATGA AGACATGCTG GAGCTGGAAC
Didelphis_marsupialis     .......... .......... .......... .......... ..........
Lasiorhinus_latifrons     .......... .......... .......... .......... ..........
Petaurus_breviceps        .......... .......... .......... .......... ..........
Phascolarctos_cinereus    .......... .......... .......... .......... ..........
Pseudocheirus_peregrinus  .......... .......... .......... .......... ..........
Spilocuscus_maculatus     .......... .......... .......... .......... ..........
Trichosurus_vulpecula     .......... .......... .......... .......... ..........
Monodelphis_domestica     .......... .......... .......... .......... ..........

                          251                                                300
Aepyprymnus_rufescens     ACGTGAAGTT TTATAAGGCA ATTATCCTTG TGCCTTGTCA GACTTGGAGT
Dendrolagus_goodfellowi   ATGTGAAGTT TTATGAGGYA ATTTTCCTTG TCCCCAG..A GACTTGGAGT
Dendrolagus_matschiei     ATGTGAAGTT TTATGAGGCA ATTTTCCTTG TCCCCAG..A GACTTGGAGT
Hypsiprymnodon_moschatus  ACATCAAGTT TTATGAGGCA ATTATCTGCA TGCCCAG..A GACTTGGAGT
Lagostrophus_fasciatus    ACGTGAAGTT TTCTGAGGCA ATTTCCCTTG TGCCCAG..A GACTTGGAGT
Macropus_eugenii          ACGTGAAGTT TTATGAGGCA ATTTTCCTTG TGCCCAG..A GACTTGGAGT
Macropus_parma            ACGTGAAGTT TTATGGGGCA ATTTTCCTTG TGTCCAG..A GACTTGGAGT
Macropus_robustus         ACGTGAAGTT TTATGAGGCA ATTTTCCTTG TGCCCAG..A GATTTGGAGT
Macropus_rufogriseus      ACGTGAAGTT TTATGAGGCA ATTTTCCTTG TGCCCAG..A GACTTGGAGT
Macropus_rufus            ACGTGAAGTT TTATGAGGCA ATTTTCCTTG TGCCCAG..A GATTTGGAGT
Macropus_giganteus        ACGTGAAGTT TTATGAGGCA ATTTTCTTTG TGCCCAG..A GACTTGGAGT
Onychogalea_unguifera     ACATGAAGTT TTATGAGGCA ATTTTCCTTG TGCCCAR..A GACTTGGAGT
Petrogale_lateralis       ATGTGAAGTT TTATGAGGCA ATTTTCCTTG TGCCCAG..A GACTTGGAGT
Potorous_tridactylus      AAGAGAAGTT TTATGAGGCA ATTATCCTTG TGCCCAG..A GACTTCAAGT
Setonix_brachyurus        ACCTGAAGTT TTATGAGGCA ATTTTCCTTG TGCCCAG..A GACTTGGAGT
Thylogale_stigmatica      ATGTGAAGTT TTATGAGGCA ATTTTCCTTG TGCCCAG..A GACTTGGAGT
Didelphis_marsupialis     .......... .......... .......... .......... ..........
Lasiorhinus_latifrons     .......... .......... .......... .......... ..........
Petaurus_breviceps        .......... .......... .......... .......... ..........
Phascolarctos_cinereus    .......... .......... .......... .......... ..........
Pseudocheirus_peregrinus  .......... .......... .......... .......... ..........
Spilocuscus_maculatus     .......... .......... .......... .......... ..........
Trichosurus_vulpecula     .......... .......... .......... .......... ..........
Monodelphis_domestica     .......... .......... .......... .......... ..........

                          301                                                350
Aepyprymnus_rufescens     CTGACATCCC TGGCTATCCC AAGAAACTGG TCACTCAGAT AGAAGAGACC
Dendrolagus_goodfellowi   CTGACATCCC TGGCTATCCC AAGAGACTGG TCACTCAGAT AGAAGAGACC
Dendrolagus_matschiei     CTGACATCCC TGGCTATCCC AAGAGACTGG TCACTCAGAT AGAAGAGACC
Hypsiprymnodon_moschatus  CTGATGCCCC TGGCTACCCT GAGAGACTGG TCACTCAGGT AGAAGAGACC
Lagostrophus_fasciatus    CTGACATCCC TGGCTATCCT AAGAGACTGG TCGCTCAGAT AGAAGAGACC
Macropus_eugenii          CTGACATCCC TGGCTATCCT AAGAGACTGG TCACTCAGAC RRAACAGACC
Macropus_parma            CTGACATCCC TGGCTATCCT AAGAGACTGG TCACTCAGAT AGAAGAGACC
Macropus_robustus         CTGACATCCC TGGCTATCCT AAGAGACTGG TCACTCAGAT AGAAGAGACC
Macropus_rufogriseus      CTGACATCCC TGGCTATCCT AAGAGACTGG TCACTCAGAC AGAAGAGACC
Macropus_rufus            CTGACATCCC TGGCTATCCT AAGAGACTGG TCACTCAGAT AGAAGAGACC
Macropus_giganteus        CTGACATCCC TGGCTATCCT AAAAGACTGG TCACTCAGAT AGAAGAGACC
Onychogalea_unguifera     CTGACATCCC TGGCTATCCT AAGAGACTGG TCACCCAGAT AGAARAGACC
Petrogale_lateralis       CTGACATCCC TGGCTATACT AAGAGACTGG TCACTCAGAT ARAAGAGACC
Potorous_tridactylus      CTGACATCCC TGGCTATCCT AAGAGACCGG TCACTCAGAT AGAAGAGACC
Setonix_brachyurus        STGACATCCC TGGCTATCCT AARAGACTGG TCACTCARAT AGAARAGACC
Thylogale_stigmatica      CTGACATCCC TGGCTATCCC AAGAGACTGG TCACTCAAAT AGAAGAGACC
Didelphis_marsupialis     .......... .......... .......... .......... ..........
Lasiorhinus_latifrons     .......... .......... .......... .......... ..........
Petaurus_breviceps        .......... .......... .......... .......... ..........
Phascolarctos_cinereus    .......... .......... .......... .......... ..........
Pseudocheirus_peregrinus  .......... .......... .......... .......... ..........
Spilocuscus_maculatus     .......... .......... .......... .......... ..........
Trichosurus_vulpecula     .......... .......... .......... .......... ..........
Monodelphis_domestica     .......... .......... .......... .......... ..........
```

```
                             351                                                400
Aepyprymnus_rufescens        AAGTGGACAC TTTCATAAAA GGACGACCTG GACTTGGAAG TGGTGGAAAG
Dendrolagus_goodfellowi      AAGTGGACAC TTTCAGAAAA GGATGACCTG GACTTGGAAG CGYTGGAGAG
Dendrolagus_matschiei        AAGTGGACAC TTTCAGAAAA GGATGACCTG GACTTGGAAG CGCTGGAGAG
Hypsiprymnodon_moschatus     AAGTGGATGC TTTTGGAAAA AAACGACCTG GACTTGGAAG TGGTTGAGAG
Lagostrophus_fasciatus       AAGCGGACAC TTTCAGAAAA GGACGAGCTG GACTTGGAAG CGGTGGTGAG
Macropus_eugenii             ARGTGGATGC TTTCAGAAAA GGACAACCTG GACTTGGAAG CTTTGGAGAG
Macropus_parma               AAGTGGACGC TTTCAGAAAA GGACAACCTG GACTTGGAAG CTTTGGAGAG
Macropus_robustus            AAGTGGACGC TTTCAGAAAA GGACAACCTG GACTTGGAAG CTTTGGAGAG
Macropus_rufogriseus         AAGTGGACGC TTTCAGAAAA GGACAACCTG GACTTGGAAG CTTTGGAGAG
Macropus_rufus               AAGTGGATGC TTTCAGAAAA GGACAACCTG GACTTGGAAG CTTTGGAGAG
Macropus_giganteus           AAGTGGACGC TTTCAGAAAA GGACAACCTG GACCTGGAAG CTTTGGAGAG
Onychogalea_unguifera        AAGTGGACAC TTTCARAAAA GGACAACCTG GACTTGGAAG CTTTGGAGAG
Petrogale_lateralis          AAGTGGACAC TTTCAGAAAA GGACGACCTG GACTTGGAAG CGTTGGAGAG
Potorous_tridactylus         AAGTGGACAC TTTCGGGAAA GGACGACCCG GACTTGGAAG CAGTGAAGAG
Setonix_brachyurus           AAGTGGACGC TTTCARAAAA GGACAACCTG GACTTGGAAG CTTTGGAGAG
Thylogale_stigmatica         AAGTGGACAC TTTCAGAAAA GGACGACCTG GACTTGGAAG CATTGGAGAG
Didelphis_marsupialis        .......... .......... .......... .......... ..........
Lasiorhinus_latifrons        .......... .......... .......... .......... ..........
Petaurus_breviceps           .......... .......... .......... .......... ..........
Phascolarctos_cinereus       .......... .......... .......... .......... ..........
Pseudocheirus_peregrinus     .......... .......... .......... .......... ..........
Spilocuscus_maculatus        .......... .......... .......... .......... ..........
Trichosurus_vulpecula        .......... .......... .......... .......... ..........
Monodelphis_domestica        .......... .......... .......... .......... ..........

                             401                                                450
Aepyprymnus_rufescens        TGGACAGGTA ACATTTGCT. ......CATC TATTCTACTG CAAATTCTTT
Dendrolagus_goodfellowi      TGGACAGGTA ACATTTGCC. ......CATC TATTCTCCCA CAAATTCCTT
Dendrolagus_matschiei        TGGACAGGTA ACATTTGCC. ......CATC TATTCTCCCA CAAATTCCTT
Hypsiprymnodon_moschatus     TGGACAGGTA ACATTAGCCC AGGTAACATT TGCTCTTCTG CAAA.TCCTT
Lagostrophus_fasciatus       TGGACAGCTA ACATTTGCC. ......CATC TATTCTCCCG CAAATTCCTT
Macropus_eugenii             TCGACAAGTA ACATTTGCC. ......CATC TATTCTCTCG CAAATTCCTT
Macropus_parma               TCGACAGGTA ACATTTGCC. ......CATC TATTCTCCCG CAAATTCCTT
Macropus_robustus            TCGACAGGTA ACATTTGCC. ......CGTC TATTCTCCCG CAAATTCCTT
Macropus_rufogriseus         TCGACAAGTA ACATTTGCC. ......CATC TATTCTCCTG CAAATTCCTT
Macropus_rufus               TCGACAGGTA ACATTTGCC. ......CATC TATTCTCCCG CAAATTCCTT
Macropus_giganteus           TCGACAGGTA ACACTTGCC. ......CATC TATTCTCCCG CAAATTCCTT
Onychogalea_unguifera        TTGACAGGTA ACATTTGCC. ......CATC TATTCTCCCG CAAATTCCTT
Petrogale_lateralis          TGGACAGGTA GCATTTGCC. ......CATC TATYCTGCCG CAAATTCCTT
Potorous_tridactylus         TGGACAGGTA ACATTTGCC. ......CATC TATTCTCCCT CAAATTCTTT
Setonix_brachyurus           TCGACAGGTA ATATTTGCC. ......CATC TATTCTCCCG CAAATTCCTC
Thylogale_stigmatica         TGGACAGGTA ACATTTGCC. ......CATC TATTCTCTCA CAAATTCCTT
Didelphis_marsupialis        .......... .......... .......... .......... ..........
Lasiorhinus_latifrons        .......... .......... .......... .......... ..........
Petaurus_breviceps           .......... .......... .......... .......... ..........
Phascolarctos_cinereus       .......... .......... .......... .......... ..........
Pseudocheirus_peregrinus     .......... .......... .......... .......... ..........
Spilocuscus_maculatus        .......... .......... .......... .......... ..........
Trichosurus_vulpecula        .......... .......... .......... .......... ..........
Monodelphis_domestica        .......... .......... .......... .......... ..........

                             451                                                500
Aepyprymnus_rufescens        GAGCACTGGG AGTACCTGAC AAGAAATCGA GAATTCAAAT ACTATGTCCA
Dendrolagus_goodfellowi      AAGCACTGGG AGTACCTGAC AAGAAATCAA GAATTCAAAT ACCATGTCCA
Dendrolagus_matschiei        AAGCACTGGG AGTACCTGAC AAGAAATCAA GAATTCAAAT ACCATGTCCA
Hypsiprymnodon_moschatus     AAGCACGGGG AGTACCTAAC AAGAAACTAA GAATTCAAAT ACTATGTCCA
Lagostrophus_fasciatus       AAGCACTGGG AGTACCTGAC AAGAAATCGA AAGTTCAAAT ACTATGTCCA
Macropus_eugenii             AATCACTGGG AGTACCTGAC AAGAAATCGA GAATTCAAAT ACTATGTCCA
Macropus_parma               AAGCACTGGG AGTACCTGAC AAGAAATCGA GAATTCAAAT ACTATGTCCA
Macropus_robustus            AAGCACTGGG AGTACCTGAG AAGAAATCGA GAATTCAAAT ACTATGTCCA
Macropus_rufogriseus         AAGCACTGGG AGTACCTGAC AAGAAATCGA GAATTCAAAT ACTATGTCCA
Macropus_rufus               AAGCACTGGG AGTACCTGAG AAGAAATCGA GAATTCAAAT ACTATGTCCA
```

```
Macropus_giganteus        AAGCACTGGG AGTACCTGAC AAGAAATCGA GAATTCAAAT ACTATGTCCA
Onychogalea_unguifera     AAGCACTGGG AGTACCTGAC AAGAAATCGA GAATTCAAAT ACTATGTTCA
Petrogale_lateralis       AAGCACTGGG AGTACCTGAC AAGAAATCGA GAATTCAAAT ACCATGTCCA
Potorous_tridactylus      AAGTACTGGG AGTACCTGAA AAGAAATCGA GAATTCAAAT ACCATGTCCA
Setonix_brachyurus        AAGCACTGGG AGTACCTGAC AAGAAATCGA GAATTCAAAT ACTATGTCCA
Thylogale_stigmatica      AAGCACTGGG AGTACCTGAC AAGAAATCGA GAATTCAAAT ACCATGTCCA
Didelphis_marsupialis     .......... .......... .......... .......... ..........
Lasiorhinus_latifrons     .......... .......... .......... .......... ..........
Petaurus_breviceps        .......... .......... .......... .......... ..........
Phascolarctos_cinereus    .......... .......... .......... .......... ..........
Pseudocheirus_peregrinus  .......... .......... .......... .......... ..........
Spilocuscus_maculatus     .......... .......... .......... .......... ..........
Trichosurus_vulpecula     .......... .......... .......... .......... ..........
Monodelphis_domestica     .......... .......... .......... .......... ..........

                          501                                                550
Aepyprymnus_rufescens     GCTGAAAAAG GGTGAGATCT ATTACCATTT ACATATACTT TTTGGGACCA
Dendrolagus_goodfellowi   GCTGAWAAAG GGGGAGATCT ATTACCATTT ACATATGCTT TTTGAGACCA
Dendrolagus_matschiei     GCTGATAAAG GGGGAGATCT ATTACCATTT ACATATGCTT TTTGAGACCA
Hypsiprymnodon_moschatus  GCTGGAAAAG GGTGAGATAT ATTACCATTT ACATACTCTT TTTGAGACCA
Lagostrophus_fasciatus    GCTGAAAAAG GGTGAGATCT ATTACCATTT ATACATGCTT TTTGAGACCA
Macropus_eugenii          GCTGAAAAAG GGTGAGATCT ATTACCATTT ACATATGCTT TTTGAGACCA
Macropus_parma            GCTTAAAAAG GGTGAGATCT ATTACCATTT ACATATGCTT TTTGAGACCA
Macropus_robustus         GCTGAAAAAG GGTGAGATCT ATTATCATTT ACATATGCTT TTTGAGACCA
Macropus_rufogriseus      GCTGAAAAAG GGTGAGATCT ATTACCATTT ACATATGCTT TTTGAGACCA
Macropus_rufus            GCTGAAAAAG GGTGAGATCT ATTATCATTT ACATATGCTT TTTGAGACCA
Macropus_giganteus        GCTGAAAAAG GGTGAGATCT ATTACCATTT ACATATGCTT TTTGAGACCA
Onychogalea_unguifera     GCTGAAAAAG GGTGAGATCT ATTACCATTT ACATATGCTT TTTGAGACCA
Petrogale_lateralis       GCTGAAAAAG GGGGAGATCT ATTACCATTT ACATATGCTT TTTGAGACCA
Potorous_tridactylus      GATGAAAAAG GGTGAGATCT ATTACCATTT ACATATGCTT TTTGAGACCA
Setonix_brachyurus        GCTGAAAAAG GGTGAGATCT ATTACCATTT ACATATGCTT TTTGAGACCA
Thylogale_stigmatica      GCTGAAAAAG GGGGAGATCT ATTACCATTT ACATATGATT TTTGAGACCA
Didelphis_marsupialis     .......... .......... .......... .......... ..........
Lasiorhinus_latifrons     .......... .......... .......... .......... ..........
Petaurus_breviceps        .......... .......... .......... .......... ..........
Phascolarctos_cinereus    .......... .......... .......... .......... ..........
Pseudocheirus_peregrinus  .......... .......... .......... .......... ..........
Spilocuscus_maculatus     .......... .......... .......... .......... ..........
Trichosurus_vulpecula     .......... .......... .......... .......... ..........
Monodelphis_domestica     .......... .......... .......... .......... ..........

                          551                                                600
Aepyprymnus_rufescens     GTGGAAGTCA GTCAATGGTG CTCAGCCGTT ACATCAGCCA GATCAAGACC
Dendrolagus_goodfellowi   GTGGAATTCA GTGCATGGTG CTCAGYCGTT ACATCAGCCA GATCAAGACT
Dendrolagus_matschiei     GTGGAATTCA GTGCATGGTG CTCAGCCGTT ACATCAGCCA GATCAAGACT
Hypsiprymnodon_moschatus  GTGGAATTCA GGCCACGGTG CTCAGCCATT ACATCAGCCA G..CAAGACC
Lagostrophus_fasciatus    GTAGAATTCA GTCCATGGTG CTCAGCCGTT ACATCAGCCA GATCAAGACC
Macropus_eugenii          GTGGAATTCA GTGCATGGTG CTCAGTCGTT ACATCAGTCA GATCAAGACC
Macropus_parma            GTGGAATTCA GTCCATGGTG CTCAGCCGTT ACACCAGCCA GATCAAGACC
Macropus_robustus         GTGGAATTCA GTCCATGGTG CTCAGCCGTT ACATCAGCCA GATCAAGACC
Macropus_rufogriseus      GTGGAATTCA GTGCATGGTG CTCAGCCGTT ACATCAGTCA GATCAAGACC
Macropus_rufus            GTGGAATTCA GTCCATGGTG CTCAGCCATT ACATCAGCCA GATCAAGACC
Macropus_giganteus        GTGGAATTCA GTCCATGGTG CTCAGCCGTT ACACCAGCCA GATCAAGACC
Onychogalea_unguifera     GTGGAATTCA GTCCATGGTG CTCAGCCGTT ACATCAGCCA GATCAAGACC
Petrogale_lateralis       GTGGAATTCA GTGCATGGTG CTCAGCCATT ACATCAGCCA GATCAAGACC
Potorous_tridactylus      GTGGAATTCA GTCCATGGTG CTCAGCCATT ACATCAGCCA GATCAAGACC
Setonix_brachyurus        GTGGAATTCA GTGCATGGTG CTCAKCCATT ATATCAGTCA GATCAAGACC
Thylogale_stigmatica      GTGGAATTCA GTGCATGGTG CTCAGCCATT ACATCAGCCA GATCAAAACC
Didelphis_marsupialis     .......... .......... .......... .......... ..........
Lasiorhinus_latifrons     .......... .......... .......... .......... ..........
Petaurus_breviceps        .......... .......... .......... .......... ..........
Phascolarctos_cinereus    .......... .......... .......... .......... ..........
Pseudocheirus_peregrinus  .......... .......... .......... .......... ..........
```

```
Spilocuscus_maculatus     .......... .......... .......... .......... ..........
Trichosurus_vulpecula     .......... .......... .......... .......... ..........
Monodelphis_domestica     .......... .......... .......... .......... ..........

                          601                                              650
Aepyprymnus_rufescens     TCGCTGCAAG CTGAAGTCTC TAACAATGCT GAGGTTAATA TC.......G
Dendrolagus_goodfellowi   TTGCCCCAAG CTGAAGTCTC TAACAATGCT GAGGTTAATA TG.......G
Dendrolagus_matschiei     TTGCCCCAAG CTGAAGTCTC TAACAATGCT GAGGTTAATA TG.......G
Hypsiprymnodon_moschatus  ACGCTGTAAG CGGAAGTCTA TAACAATGCT GAGGTTAATA TC.......G
Lagostrophus_fasciatus    TCGCTGCAAG CAGAAGTCTG TAACAACGCT GAGGTTAATA CA.......G
Macropus_eugenii          TCGCTGTGAG CTAAAGTCTC TAACAATGCT GAGGTTAATA ACAAAAACTG
Macropus_parma            TCGCTGTGAG CTAAAGTCTC TAACAATGCT GAGGTTAATA TCAAAAACTG
Macropus_robustus         TCGCTGCGAG CTAAAGTCTC TAACAATGCT GAGGTTAATA TCGAAAACTG
Macropus_rufogriseus      TCGCTGCGAG CTAAAGTCTC TAACAATGCT GAGGTTAATA TCGAAAACTG
Macropus_rufus            TCGCTGCGAG CTAAAGTCTC TAACAATGCT GAGGTTAATA TCGAAAACTG
Macropus_giganteus        CCGCTGCGAG CTAAAGTCTC TAACAATGCT GAGGTTAATA TCAAAAACTG
Onychogalea_unguifera     TCACTGAGAG CTAAAGTCTC TAACCATGCT GAGGTTAATA TAGAAAACTG
Petrogale_lateralis       TTGCCGCAAG CTGAAGTCTC TAACGATGCT GAGATTAATA TG.......G
Potorous_tridactylus      TTGCTGCAAG CTGAAGTCTC TAACAATGCT GAGGTTAATA TC.......G
Setonix_brachyurus        TCTCTGAGAA CTAAAGTCTC TAACAATGCT GAGGTTAATA TCGAAAACTG
Thylogale_stigmatica      TTGCTGCAAG CTGAAGTCTC TAACAATGCT GAGGTTAATA TG.......G
Didelphis_marsupialis     .......... .......... .......... .......... ..........
Lasiorhinus_latifrons     .......... .......... .......... .......... ..........
Petaurus_breviceps        .......... .......... .......... .......... ..........
Phascolarctos_cinereus    .......... .......... .......... .......... ..........
Pseudocheirus_peregrinus  .......... .......... .......... .......... ..........
Spilocuscus_maculatus     .......... .......... .......... .......... ..........
Trichosurus_vulpecula     .......... .......... .......... .......... ..........
Monodelphis_domestica     .......... .......... .......... .......... ..........

                          651                                              700
Aepyprymnus_rufescens     AAAACTTACT GGCCGTTACG ACTACTAAGG CCTCCCGGGG GAGGTGGGGG
Dendrolagus_goodfellowi   AAAACTGGCT GGCCATTACG ACGATTAAGG CCACTGGGGG G.........
Dendrolagus_matschiei     AAAACTGGCT GGCCATTACG ACGATTAAGG CCACTGGGGG G.........
Hypsiprymnodon_moschatus  AAAACTGACT GACCATTATG AAGACTAAGG CCACGGTG.. ..........
Lagostrophus_fasciatus    AAAACTGACT GGCCATTACG ACGACTAAGG CCACCGGGGG GG........
Macropus_eugenii          AAAACTGACT GGCCATTACG ACGACTAAGG CCACGGGGGG GTGGTGGGGT
Macropus_parma            AAAACTGACT GGCCATTACA ACGACTAAGG CCACCGGGGG G.........
Macropus_robustus         AAAACTGACT GGCCATTACG ACGACTAAGG CCACCGGGGG TGG.......
Macropus_rufogriseus      AAAACTGACT GGCCATTATG ACGACTAAGG CCACTGGTGG GG........
Macropus_rufus            AAAACTGACT GGCCATTACG ACGGCTAAGG CCACCGGTGG GG........
Macropus_giganteus        AAAACTGACT GGCCATTACA ACGACTAAGG CCACCGGGGG G.........
Onychogalea_unguifera     AAAACTGACT GGCCATTACG ACCACTAAGG CCACCGGGGG G.........
Petrogale_lateralis       AAAACTGGCT GGCCATTACG GCGATTAAGG CCACTGGGGG G.........
Potorous_tridactylus      AAAACTGACT GGCCATTACG AC...TAAGG CCACCTGGGG GGGGGGGGG.
Setonix_brachyurus        AAAACTGACT GGCCATTACG ACGACTAAGG CCACCGGGGG G.........
Thylogale_stigmatica      AAAACTGGCT GGCCATTATG ACGATTAAGG CCACTGGGGG GGGGGGG...
Didelphis_marsupialis     .......... .......... .......... .......... ..........
Lasiorhinus_latifrons     .......... .......... .......... .......... ..........
Petaurus_breviceps        .......... .......... .......... .......... ..........
Phascolarctos_cinereus    .......... .......... .......... .......... ..........
Pseudocheirus_peregrinus  .......... .......... .......... .......... ..........
Spilocuscus_maculatus     .......... .......... .......... .......... ..........
Trichosurus_vulpecula     .......... .......... .......... .......... ..........
Monodelphis_domestica     .......... .......... .......... .......... ..........

                          701                                              750
Aepyprymnus_rufescens     GGGGGGTCAA CCAAACAAGT GGACTTTAAC TGCATCAA.T TGGTATTTAA
Dendrolagus_goodfellowi   .....GTCAA CCAAACAGGT GGACTATAGT TGCATCAA.C TGGTATTTAA
Dendrolagus_matschiei     .....GTCAA CCAAACAGGT GGACTATAGT TGCATCAA.C TGGTATTTAA
Hypsiprymnodon_moschatus  .....GTCAA CCAAACAGGT GGACTATGAT TACGTCAA.C TGGTATGTAG
Lagostrophus_fasciatus    .....ATCAA CCAAACAGGT GGACTATAAT TGCATCAA.C TGGTATTTAA
Macropus_eugenii          .....GTCAA CCAAACAGGT GGACTATAAT TGCATCAA.C TGGTATTTAA
```

```
Macropus_parma            .....GTCAA CCAAACAGGT GGACTATAAT TGCATCAA.C TGGTATTTAA
Macropus_robustus         .....GTCAA CCAAACAGGT GGACTATAAT TGCATCAG.C TGGTATTTAA
Macropus_rufogriseus      .....GTCAA ACAAACAGGT GGACTATAAT TGCATCAA.C TGGTATTTAA
Macropus_rufus            .....GTCAA CCAAACAGGT GGACTATAAT TGCATCAA.C TGGTATTTAA
Macropus_giganteus        .....GTCAA CCAAACAGGT GGACTATAAT TGCATCAA.C TGGTATTTAA
Onychogalea_unguifera     .....GTCAA CCAAACAGGT GGACTGTAAT TGCATCAACC TGGTATTTAA
Petrogale_lateralis       .....GTCAA CCAAGCAGGT GGACTATAGT TGCATCAA.C TGGTATTTAA
Potorous_tridactylus      .....GTCAA TCAAACAGGT AGACTATAAC TGCATCAA.T TGGTATTTAA
Setonix_brachyurus        .....GTCAA CCAAACAGGT GGATTATAAT TACATCAA.C TGGTATTTAA
Thylogale_stigmatica      .....GTCAA CCAATCAGGT GGACTATAGT TGCATCAA.C TGGTATTTAA
Didelphis_marsupialis     .......... .......... .......... .......... ..........
Lasiorhinus_latifrons     .......... .......... .......... .......... ..........
Petaurus_breviceps        .......... .......... .......... .......... ..........
Phascolarctos_cinereus    .......... .......... .......... .......... ..........
Pseudocheirus_peregrinus  .......... .......... .......... .......... ..........
Spilocuscus_maculatus     .......... .......... .......... .......... ..........
Trichosurus_vulpecula     .......... .......... .......... .......... ..........
Monodelphis_domestica     .......... .......... .......... .......... ..........

                          751                                                800
Aepyprymnus_rufescens     TGGCATAAAA ACAACCAGAA TTTCCGTGGG TGTGGACAAA TATTGAGGAA
Dendrolagus_goodfellowi   TAGCATAAAA ACAATCAGAA TTTCAGTGGG GGTGGACAAA TATTGAGAAA
Dendrolagus_matschiei     TAGCATAAAA ACAATCAGAA TTTCAGTGGG GGTGGACAAA TATTGAGAAA
Hypsiprymnodon_moschatus  TGGGGAAAAA ACAACCAGAA TTCCAGTGGG CGTGGACAAG TATTGAGGAA
Lagostrophus_fasciatus    TGGCATAAAA ACAACCAGAA TTTCAGTGGG TGTGGACAAA TATTGAGGAA
Macropus_eugenii          TAGCATAAAA GCAACCAGAA TTTCAGTGGG GGTGGACAAA TATTGAGGAA
Macropus_parma            TAGCATAAAA ACAACCAGAA TTTCAGTGGG GGTGGACAAA TATTGAGGAA
Macropus_robustus         TAGCATAAAA ACAACCAGAA TTTC.GTGGG GGTGGACAAA TATTGAGGAA
Macropus_rufogriseus      TAGCATAAAA ACAACCAGAA TTTCAGTGGG GGTGGACAAA TATTGAGGAA
Macropus_rufus            TAGCATAAAA ACAACCAGAA TTTCAGTGGG GGTGGACAAA TATTGAGGAA
Macropus_giganteus        TAGCATAAAA ACAACCAGAA TTTCAGTGGG GGTGGACAAA TATTGAGGAA
Onychogalea_unguifera     TAGCATA... ..AACCAGAA TTTCAGTGGG GGTGGATAAA TATTGAGGAA
Petrogale_lateralis       TAGCATAAAA ACAATCAGAA TTTCAGTGGG GGTGGACAAA TATTGAGGAA
Potorous_tridactylus      TGGCATAAAA ACAACCAGAA TTTCAGTGGG TATGGACAAA TATTGAGGAA
Setonix_brachyurus        TAGCATAAAA ACAACCAGAA TTTCAGTGGG GGTGGACAAA TATTGAGGAA
Thylogale_stigmatica      TAGCATAAAA ACAACCAGAA TTTCAGTGGG GGTGGACAAA TATTGAGGAA
Didelphis_marsupialis     .......... .......... .......... .......... ..........
Lasiorhinus_latifrons     .......... .......... .......... .......... ..........
Petaurus_breviceps        .......... .......... .......... .......... ..........
Phascolarctos_cinereus    .......... .......... .......... .......... ..........
Pseudocheirus_peregrinus  .......... .......... .......... .......... ..........
Spilocuscus_maculatus     .......... .......... .......... .......... ..........
Trichosurus_vulpecula     .......... .......... .......... .......... ..........
Monodelphis_domestica     .......... .......... .......... .......... ..........

                          801                                                850
Aepyprymnus_rufescens     TATAAGGACT TGATCCTCAA TATCCCCGCC AGACTGTAGC CCGCAGGCTA
Dendrolagus_goodfellowi   TATAAGGACT TGATCCTCAA TATCCCCGCC AGACTGCAGC TCGCAGGCTA
Dendrolagus_matschiei     TATAAGGACT TGATCCTCAA TATCCCCGCC AGACTGCAGC TCGCAGGCTA
Hypsiprymnodon_moschatus  TATAAAGACT TGATCCTTAA TATTCCCACC ACACTGAAGC CTGATGTCCA
Lagostrophus_fasciatus    TATAAGGACT TGATCCTCAA TATCCCTGCC AGACTGCAGC TCGCAGGCTA
Macropus_eugenii          TATAAGGACT TAATCCTTAA TATCCCCGCC AGACCGCAGC TTATAGGCTA
Macropus_parma            TATAAGGACT TGATCCTCAA TATCCCCGCC AGACCACAGC TCATAGGCTA
Macropus_robustus         TATAAGGACT TGATCCTCAA TATCCCCGCC AGACCACAGC TCATAGGCTA
Macropus_rufogriseus      TATAAGGACT TGATCCTCAA TATCCCTGCC AGACCGCAGC TCGTAGGCTA
Macropus_rufus            TATAAGGACT TGATCCTCAA TATCCCCGCC AGACCGCAGC TCATAGGCTA
Macropus_giganteus        TATAAGGACT TGATCCTCAA TATCCCCGCC AGACCGCAGC TCGTAGGCTA
Onychogalea_unguifera     TGTAAGGACT TGATCCTCAA TATCCCCGCC AGACCGCAGC TCGTAGGCTA
Petrogale_lateralis       TATAAGGACT TGATCATCAA TATCCCCACC AGACTGCAGC TCGCAGGCTA
Potorous_tridactylus      TATAAGGACT TGATCCTCAA TATCCCCRCC AGACTGTAGC TCGCAGGCTA
Setonix_brachyurus        TATAAGGACT TGATCCTCAA TATCCCCGCC AGACCGCAGC TCGTAGGCTA
Thylogale_stigmatica      TATAAGGACT TGATCCTCAA TATCCCCGCC AGAGGGCAGC TCACAGGGTA
Didelphis_marsupialis     .......... .......... .......... .......... ..........
```

```
Lasiorhinus_latifrons      .......... .......... .......... .......... ..........
Petaurus_breviceps         .......... .......... .......... .......... ..........
Phascolarctos_cinereus     .......... .......... .......... .......... ..........
Pseudocheirus_peregrinus   .......... .......... .......... .......... ..........
Spilocuscus_maculatus      .......... .......... .......... .......... ..........
Trichosurus_vulpecula      .......... .......... .......... .......... ..........
Monodelphis_domestica      .......... .......... .......... .......... ..........

                           851                                              900
Aepyprymnus_rufescens      GTTCTTCACA TCGACCTACT TGGCTCCCGG GGTGAGTGAC TCCCAATCCT
Dendrolagus_goodfellowi    GTTCTTCACA TCGACCTACT TGGCTCCTGG GGTGAGTGAT TCCCAATCCT
Dendrolagus_matschiei      GTTCTTCACA TCGACCTACT TGGCTCCTGG GGTGAGTGAT TCCCAATCCT
Hypsiprymnodon_moschatus   GTTCTTCACA TCGACCTACT TGACTCCTGG AGTGAGTGAC TCTCAATCCT
Lagostrophus_fasciatus     GTTCTTTACA TCAACCTACT TGGCTCCTGG GGTGAGTGAC TCCCAATCCT
Macropus_eugenii           GTTCTTCACA TAGACCTACT TCGCTCCTGG GGTGAGTGAC TCCCAATCCT
Macropus_parma             GTTCTTCACA TAGACCAACT TTGCTCCTGG GGTGAATGAC TCCCAATCCT
Macropus_robustus          GTTCTTCACA TAGACCTACT TCGCTCCTGG GGTGAGTGAC TCCCAATCCT
Macropus_rufogriseus       GTTCTTCACA TAGACCTACT TCGCTCCTGG GGTGAGTGAC TCCCAATCCT
Macropus_rufus             GTTCTTCACA TAGACCTACT TCGCTCCTGG GGTGAGTGAC TCCCAATCCT
Macropus_giganteus         GTTCTTCACA TAGACCTACT TCGCTCCTGG GGTGAGTGAC TCCCAATCCT
Onychogalea_unguifera      GTTCTTCACA TAGACCTACT TCGCTCCTGG GGTGAGTGAC TCCCAATCCT
Petrogale_lateralis        GTTMTTCACA TCGACCTACT TGGCTCCTGG GGTGAGTGAC TCCCAATCCT
Potorous_tridactylus       GCTCTTCACA TCGACCTACT TGGCTCCCGG GGTGAGTAAC TCCCAATCCT
Setonix_brachyurus         GTTCTTCACA TAGACCTACT TCACTCCTGG GGTGAGTGAC TCCCAATCCT
Thylogale_stigmatica       GTTCTTCACA TCGACCTGCT TGGCTCCTGG GGTGAGTGAC TCCCAATCCT
Didelphis_marsupialis      .......... .......... .......... .......... ..........
Lasiorhinus_latifrons      .......... .......... .......... .......... ..........
Petaurus_breviceps         .......... .......... .......... .......... ..........
Phascolarctos_cinereus     .......... .......... .......... .......... ..........
Pseudocheirus_peregrinus   .......... .......... .......... .......... ..........
Spilocuscus_maculatus      .......... .......... .......... .......... ..........
Trichosurus_vulpecula      .......... .......... .......... .......... ..........
Monodelphis_domestica      .......... .......... .......... .......... ..........

                           901                                              950
Aepyprymnus_rufescens      CTTAAATCTC TAATACTTCT GGTGCTCCCT CCTATTGTGA CAGAAATACC
Dendrolagus_goodfellowi    CYTAAATCTC TAATACTTCT GGTGCT..CT TCTATTGTGA TAGAAATACT
Dendrolagus_matschiei      CTTAAATCTC TAATACTTCT GGTGCT..CT TCTATTGTGA TAGAAATACT
Hypsiprymnodon_moschatus   CTCAAGTCTC CAATACTTCT GGTGCTCCCA TCTATCGTGG TAGAAATACC
Lagostrophus_fasciatus     CTTAAATCTC TAATACTTCT GGTGTTCCCT TCTATTGTGA TAGAAATACT
Macropus_eugenii           CTTAAATCTC TAATACTTCT GGTGCT..CT TCTATTGTTA TAGAAATACT
Macropus_parma             CTTAAATCTC TAATACTTCT GGTGCT..CT TCTATTGTTA TAGAAATACT
Macropus_robustus          CTTAAATCTC TAATACTTCT GGTGCT...T TCTATTGTTA TAGAAATACT
Macropus_rufogriseus       CTTAAATCTC TAATACTTCT GGTGCT..CT TCTATTGTTA TGGAAATACT
Macropus_rufus             CTTAAATCTC TAATACTTCT GGTGCT...T TCTATTGTTA TAGAAATACT
Macropus_giganteus         CTTAAATCTC TAATACTTCT GGTGCT..CT TCTATTGTTA TAGAAATACT
Onychogalea_unguifera      CTTAAATCTC TAATACTTCT GGTGYT..TT TCTATTGTTA TAGAAATACT
Petrogale_lateralis        CTTAAATCTC TAATACTTCT GGTGCT..CT TCTATTGTGA TAGAAATAGC
Potorous_tridactylus       CTTAAATCTC TAATACTTCT GGTGCTCCCT TCTATTGTGA CAGAAACACC
Setonix_brachyurus         CTTAAATCTC TAATACTTCT GGTGCT..CT TCTATTGTTA TAGAAATACT
Thylogale_stigmatica       CTTAAATCTC TAATACTTCT GGTGCT..CT TCTATTGTGA CAGAAATACC
Didelphis_marsupialis      .......... .......... .......... .......... ..........
Lasiorhinus_latifrons      .......... .......... .......... .......... ..........
Petaurus_breviceps         .......... .......... .......... .......... ..........
Phascolarctos_cinereus     .......... .......... .......... .......... ..........
Pseudocheirus_peregrinus   .......... .......... .......... .......... ..........
Spilocuscus_maculatus      .......... .......... .......... .......... ..........
Trichosurus_vulpecula      .......... .......... .......... .......... ..........
Monodelphis_domestica      .......... .......... .......... .......... ..........

                           951                                             1000
Aepyprymnus_rufescens      GAGAGATACA TGGAGCTTGT AAATTGGCTT GTGGAGCAGT ACCTCTGAAA
Dendrolagus_goodfellowi    GAGAGATATG TGGAGCTTGT AAATTGGCTT GTGGAGCAGC ACCTCTGAAA
```

```
Dendrolagus_matschiei    GAGAGATATG TGGAGCTTGT AAATTGGCTT GTGGAGCAGC ACCTCTGAAA
Hypsiprymnodon_moschatus GAGAGATACA TGGAGC.... .......... .......... ..........
Lagostrophus_fasciatus   GAGAGATACA TGGAGTTTGT AAATTGGCTC GTGGAGCAGC ACCTCTGAAA
Macropus_eugenii         GAGAGATACA TGGAGCTTGT AAATTGGCTT GTGGAGCAGC ACCTCTGAAA
Macropus_parma           GAGAGATACA TGGAGCTTGT AAATTGGCTT GTGGAGCAGC ACCTCTGAAA
Macropus_robustus        GAGAGATACA TGGAGCTTGT AAATTGGCTT GTGGAGCAGC ACCTCTGAAA
Macropus_rufogriseus     GAGAGATACA TGGAGCTTGT AAATTGGCTT GTGGAGCAGC ACCTCTGAAA
Macropus_rufus           GAGAGATACA TGGAGCTTGT AAATTGGCTT GTGGAGCAGC ACCTCTGAAA
Macropus_giganteus       GAGAGATACA TGGAGCTTGT AAATTGGCTT GTGGAGCAGC ACCTCTGAAA
Onychogalea_unguifera    GAGAGATACA TGGAGCTTGT AAATTGGTTT GTGGAGCAGC ACCTCTGAAA
Petrogale_lateralis      AAGAGATATG TGGAGCTTGT AAATTGGCTT GTGGAGCAGC ACCTCTGAAA
Potorous_tridactylus     GAGAGATACA TGGAGCTTGT AAATTGGCTT GTGGAGCAGC ACCTCTGAAA
Setonix_brachyurus       GAGAGATACA TGGAGCTTGT AAATTGGCTT GTGGAGCAGC ACCTCTGAAA
Thylogale_stigmatica     GAGAGATACG TGGAGCTTGT AAATTGGCTT GTGGAGCAGC ACCTCTGAAA
Didelphis_marsupialis    .......... .......... .......... .......... ..........
Lasiorhinus_latifrons    .......... .......... .......... .......... ..........
Petaurus_breviceps       .......... .......... .......... .......... ..........
Phascolarctos_cinereus   .......... .......... .......... .......... ..........
Pseudocheirus_peregrinus .......... .......... .......... .......... ..........
Spilocuscus_maculatus    .......... .......... .......... .......... ..........
Trichosurus_vulpecula    .......... .......... .......... .......... ..........
Monodelphis_domestica    .......... .......... .......... .......... ..........

                         1001                                               1050
Aepyprymnus_rufescens    A.GCAGTGGA TTATTGAAAA TCAGGAAAGT TATCTC.TCT CACCAAT.CC
Dendrolagus_goodfellowi  A.GCAGTGGR TTATTGAAAA TCAGGAAAGT TATCTC.TGT CACCAAT.CC
Dendrolagus_matschiei    A.GCAGTGGA TTATTGAAAA TCAGGAAAGT TATCTC.TGT CACCAAT.CC
Hypsiprymnodon_moschatus .......... .......... .......... .......... ..........
Lagostrophus_fasciatus   A.GCAGTGGA TTACTGAAAA TCAGGAAAGT TATCTC.TCT CACCAAA.TC
Macropus_eugenii         A.GCAGTGGA TTATTGAAAA TCAGGAAAGT TATCTC.TGT CACCAAT.CC
Macropus_parma           A.GCAGTGGA TTATTGAAAA TCAGGAAAGT TATCTC.TGT CACCAAT.CC
Macropus_robustus        A.GCAATGCA TTATTGAAAA TCAGGAAAGT TATCCC.CGT CACCAAT.CC
Macropus_rufogriseus     A.GCAGTGGA TTATTGAAAA TCAGGAAAGT TATCTC.TGT CACCAAT.CC
Macropus_rufus           A.GCAGTGCA TTATTGAAAA TCAGGAAAGT TATCTC.CGT CACCAAT.CC
Macropus_giganteus       A.GCAGTGGA TTATTGAAAA TCAGGAAAGT TATCTC.TGT CACCAAT.CC
Onychogalea_unguifera    A.GCAGTGGA TTATTGAAAA TCAGGAAAGT TATCTC.TGT CACCAAT.CC
Petrogale_lateralis      A.GCAGTGGA TTATTGAAAA TCGGRAAAGT TATCTC.TGT CACCAAT.CC
Potorous_tridactylus     A.GCAGTGGA TTATTGAAAA TCAGGAAAGT TATCTC.TCT CACCAAT.CC
Setonix_brachyurus       A.GCAGTGGA TTATTGAAAA TCAGGAAAGT TATCTC.TGT CACCAAT.CC
Thylogale_stigmatica     AGGCAGTGGA TTATTGAAAA TCAGGAAAGT TATCTCATGT CACCAATGCC
Didelphis_marsupialis    .......... .......... .......... .......... ..........
Lasiorhinus_latifrons    .......... .......... .......... .......... ..........
Petaurus_breviceps       .......... .......... .......... .......... ..........
Phascolarctos_cinereus   .......... .......... .......... .......... ..........
Pseudocheirus_peregrinus .......... .......... .......... .......... ..........
Spilocuscus_maculatus    .......... .......... .......... .......... ..........
Trichosurus_vulpecula    .......... .......... .......... .......... ..........
Monodelphis_domestica    .......... .......... .......... .......... ..........

                         1051                                               1100
Aepyprymnus_rufescens    ACTAGCAATG GAGCCAGGCG GATTAAAGTC GCTCTGAACA ATGCTTCTAA
Dendrolagus_goodfellowi  ACTAGAAATG GAGCCAGGAA GATTAAAGTC GCTCTGGACA ATGCTTCTAA
Dendrolagus_matschiei    ACTAGAAATG GAGCCAGGAA GATTAAAGTC GCTCTGGACA ATGCTTCTAA
Hypsiprymnodon_moschatus .......... .......... .......... .......... ..........
Lagostrophus_fasciatus   ACTAGCAATG GAGCCAGGCA GATTAAAGTC GCTCTGGACA ATGCTTCTAA
Macropus_eugenii         ACTAGCAATG CAGCCAGGAA GATTAAAGTT GCTCTGGACA ATGCTTCTAA
Macropus_parma           ACTAGCAATG GAGCCAGGAA GATTAAAGTC GCTCTGGACA ATGCTTCTAA
Macropus_robustus        ACTAGCAATG GAGCCAGGAA GATTAAAGTT GCTCTGGACA ATGCTTCTAA
Macropus_rufogriseus     ACTAGCAATG GAGCCAGGAA GATTAAAGTC GCTCTGGACA ATACTTCTAA
Macropus_rufus           ACTAGCAATG GAGCCAGGAA GATTAAAGTC ACTCTGGACA ATGCTTCTAA
Macropus_giganteus       ACTAGCAATG GAGCCAGGAA GATTAAAGTT GCTCGGGACA ATGCTTCTAA
Onychogalea_unguifera    ACTAGCAATG GAGTCAGGAA GATTAAAGTC TCTCTGGACA ATGCTTCTAG
Petrogale_lateralis      ACTAGAAATG GAGCCAGGAA GATTAAAGTC GCTCTGGACA ATGCTTCTAA
```

```
Potorous_tridactylus      ACTAGCAATG GAGCCAGGCA GATTAAAGTC GCTCTGGACA GTGCTTCTAA
Setonix_brachyurus        ACTAGCAATG GAGCCAGGAA TATTCAAGTC GCTCTGGACA ATGCTCCTAA
Thylogale_stigmatica      ACTAGAAATG GAGCCAGGAA GATTAAAGTT GCTMTGGACA ACACTTCTAA
Didelphis_marsupialis     .......... .......... .......... .......... ..........
Lasiorhinus_latifrons     .......... .......... .......... .......... ..........
Petaurus_breviceps        .......... .......... .......... .......... ..........
Phascolarctos_cinereus    .......... .......... .......... .......... ..........
Pseudocheirus_peregrinus  .......... .......... .......... .......... ..........
Spilocuscus_maculatus     .......... .......... .......... .......... ..........
Trichosurus_vulpecula     .......... .......... .......... .......... ..........
Monodelphis_domestica     .......... .......... .......... .......... ..........

                          1101                                               1150
Aepyprymnus_rufescens     AATTATGAAT CTGACCAAAA ATACGGA.TG ATTATCTTAT CCTGAAAGAA
Dendrolagus_goodfellowi   AATTATGAAT CTGACCAAAA ATGAGGA.TG ATTACCTTAT CCCGAAAGAA
Dendrolagus_matschiei     AATTATGAAT CTGACCAAAA ATGAGGA.TG ATTACCTTAT CCCGAAAGAA
Hypsiprymnodon_moschatus  .......... .......... .......... .......... ..........
Lagostrophus_fasciatus    AATTAAGAAT CTGACCAAAA ATGCAGA.TG ATTACCTTAT CCCGAAAGAA
Macropus_eugenii          AATTATGAAT CTGACCAAAA ATGCAGA.TG ATTACCTTAT CCCGAAAGAA
Macropus_parma            AACTATGAAT CTGAGCAAAA ATGCAAA.TG ATTACCTTAT CCCAAAAGAA
Macropus_robustus         AATTATGGAT CTGACCGAAA ATGCAGATTG ATTACCTTAT CCCGAAAGAA
Macropus_rufogriseus      AATTATGAAT CTGACCAAAA ATGCAGA.TG ATTACCTTAT CCCTAAAGAA
Macropus_rufus            AATTATGAAT CTGACCAAAA ATGCAGA.TG ATTACCTTAT CCCGAAAGAA
Macropus_giganteus        AATTATGAAT CTGACCAAAA ATGCAGA.TG ATTACCTTAT CCCAAAAGAA
Onychogalea_unguifera     AATTATGAGT CTGACCAAAA ATGCAGA.TG ATTACCTTAT CCTGAAAGAA
Petrogale_lateralis       AATTATGAAT CTGACCAAAA ATGARGA.TG ATTACCTTAT CCCAAAAGAA
Potorous_tridactylus      AGTTATGAAT CTGACCAAAA ATGCAGA.TG ATTACCTTAT CCCGAAAGAA
Setonix_brachyurus        AATTATGAAT CTGACCAAAA ATGCAGA.TG ATTACCTTAT CCTGAAAGAA
Thylogale_stigmatica      AATTATGAAT CCGACCAAAA ATGAGGA.TG ATTACCTTAT CCCGACAGAA
Didelphis_marsupialis     .......... .......... .......... .......... ..........
Lasiorhinus_latifrons     .......... .......... .......... .......... ..........
Petaurus_breviceps        .......... .......... .......... .......... ..........
Phascolarctos_cinereus    .......... .......... .......... .......... ..........
Pseudocheirus_peregrinus  .......... .......... .......... .......... ..........
Spilocuscus_maculatus     .......... .......... .......... .......... ..........
Trichosurus_vulpecula     .......... .......... .......... .......... ..........
Monodelphis_domestica     .......... .......... .......... .......... ..........

                          1151                                               1200
Aepyprymnus_rufescens     TTTGTCAGTT TTGACAACAT TAAACAGAAC CATATCTGTT GGATCTTTAA
Dendrolagus_goodfellowi   TTTGTCAGTT TTGACAACAT TAAACAGAAC CTTATCTATT GGATCTTTAA
Dendrolagus_matschiei     TTTGTCAGTT TTGACAACAT TAAACAGAAC CTTATCTATT GGATCTTTAA
Hypsiprymnodon_moschatus  .......... .......... .......... .......... ..........
Lagostrophus_fasciatus    TTTGTCAGTT TTGACAACAT TAAACAGAAC CATATCTATT GGAGCTTTAA
Macropus_eugenii          TTTGTCAGTT TTGACAACAT TAAACAGAAC CATATCTATT GGATCTTTAA
Macropus_parma            TTTGTCAGTT TTGATAACAT TAAACAGAAC CATATCTATT GGATCTTTAA
Macropus_robustus         TTTGTCAG.T TTGACACCAT TAAACAGAGC CATATCTATT GGATCTTTAA
Macropus_rufogriseus      TTTGTCAGTT TTGACAACAT TAAACAGAAC CATATCTATT GGATCTTTAA
Macropus_rufus            TTTGTCAGTT TTGACACCAT TAAACAGAGC CATATCTATT GGATCTTTAA
Macropus_giganteus        TTTGTCAGTT TTGATAACAT TAAACAGAAC CATATCTATT GGATCTTTAA
Onychogalea_unguifera     TYTGTCAGTT TCGATAACAT TAAACAGAAC CATATCTATT GGATCTTTAA
Petrogale_lateralis       TTTGTCAGTT TTGACAACAT TAAACAGAAC CTTATCTATT GGATCTTTAA
Potorous_tridactylus      TTTGCCAG.T TTGACAACAT TAAACAGTAC CATATCTATT GGATCTTTAA
Setonix_brachyurus        TTTGTCAGTT TTGACAACAT TGAACAGAAC CATATCTATT GGATCTTTAA
Thylogale_stigmatica      TTTGTCAGTT TTGACAACAT TAAACAGAAC T.TATCTATT GGATCTTTAA
Didelphis_marsupialis     .......... .......... .......... .......... ..........
Lasiorhinus_latifrons     .......... .......... .......... .......... ..........
Petaurus_breviceps        .......... .......... .......... .......... ..........
Phascolarctos_cinereus    .......... .......... .......... .......... ..........
Pseudocheirus_peregrinus  .......... .......... .......... .......... ..........
Spilocuscus_maculatus     .......... .......... .......... .......... ..........
Trichosurus_vulpecula     .......... .......... .......... .......... ..........
Monodelphis_domestica     .......... .......... .......... .......... ..........
```

```
                             1201                                              1250
Aepyprymnus_rufescens        GAATGGGTAC GACCCTCTTT ATTCTGGAGC TATCCTGGTG GGCTGGA.CC
Dendrolagus_goodfellowi      GAATGTTTAC TACCCTCTTT ATGCTGGATC TATCCTGGTG GGCTGGG.CC
Dendrolagus_matschiei        GAATGTTTAC TACCCTCTTT ATGCTGGATC TATCCTGGTG GGCTGGG.CC
Hypsiprymnodon_moschatus     .......... .......... .......... .......... ..........
Lagostrophus_fasciatus       GAATGGATAC GACCCTCTTT ATGCTGGATC TATCCTGGTG GTCTGGG.TT
Macropus_eugenii             GAATGGATAC .......... .....GGATC TATCCTGGTA GGCTGGG.CC
Macropus_parma               GAATGGATAC .......... .....GGATC TATCCTGGTG GGCTGGG.CC
Macropus_robustus            GAATGGATAG .......... .....GGATC TATCCTGGTG GGCTGGGCCC
Macropus_rufogriseus         GAATGGATAC .......... .....GGATC TATCCTGGTA GGCTGGG.CC
Macropus_rufus               GAATGGATA. .......... .....TGATC TATCCTGGTG GGCTGGG.CC
Macropus_giganteus           GAATGGATAG .......... .....GGATC TATCCTGGTG GGCTGGG.CC
Onychogalea_unguifera        GACTGGATAT .......... .....GGATC TATCCTGGTG GGCTGGG.CC
Petrogale_lateralis          GAATGTTTAC TACCCTCTTT ATGCTGGATC TATCCTGGTG GGCTGGG.CC
Potorous_tridactylus         GAATAGATAT GACCCTCTTT ATTCTGGAGC TATYCTCGTG GGCTGGG.CC
Setonix_brachyurus           GAATGGATAT .......... .....GGATC TATCCTGGTG GGCTGGG.CC
Thylogale_stigmatica         GAATGTTTAC TACCCTCTTT ATGCTGGATC TATCCTGGTG GGCTGGG.CC
Didelphis_marsupialis        .......... .......... .......... .......... ..........
Lasiorhinus_latifrons        .......... .......... .......... .......... ..........
Petaurus_breviceps           .......... .......... .......... .......... ..........
Phascolarctos_cinereus       .......... .......... .......... .......... ..........
Pseudocheirus_peregrinus     .......... .......... .......... .......... ..........
Spilocuscus_maculatus        .......... .......... .......... .......... ..........
Trichosurus_vulpecula        .......... .......... .......... .......... ..........
Monodelphis_domestica        .......... .......... .......... .......... ..........

                             1251                                              1300
Aepyprymnus_rufescens        AGGTAAGAAT TCGGCAAAAG AAACACCATC TGATTCTGTG GAAAGGCCAC
Dendrolagus_goodfellowi      AGAAAAGAAT TTGGCAAAAG AAACACCATC TGGTTCTATG GAAAGGCCAC
Dendrolagus_matschiei        AGAAAAGAAT TTGGCAAAAG AAACACCATC TGGTTCTATG GAAAGGCCAC
Hypsiprymnodon_moschatus     .......... .......... .......... .......... ..........
Lagostrophus_fasciatus       AGAAAAGAGT TTGGCAAAAG AAACACCATC TGGTTCTATG GAAAGGCCAC
Macropus_eugenii             AGAAAAGAAT TTGGCAAAAG AAACACCATC TGGGTCTATG GAAAGGCCAC
Macropus_parma               AGAAAAGAAT TTGGCAAAAG AAACACCATC TGGGTCTACG GAAAGGCCAC
Macropus_robustus            AGAAAAGAAT TTGGCAAAAG AAACACCATC TGGGTCTATG GAAAGGCCAC
Macropus_rufogriseus         AGAAAAGAAT TTGGCAAAAG AAACACCATC TGGGTCTATG GAAAGGCCAC
Macropus_rufus               GGAAAAGAAT GTGGCAAAAG AAACACCATC TGGGTCTATG GAAAGGCCAC
Macropus_giganteus           AGAAAAGAAT TTGGCAAAAG AAACACCATC TGGGTCTATA GAAAGGCCAC
Onychogalea_unguifera        AGAAAAGAAT TTGGCAAAAG AAACACTATC TGGGTCTATG GAAAGGCCAC
Petrogale_lateralis          AGAAAAGAAT TTGGCAAAAG AAACACCATC TGGTTCTATG GAAAGGCCAC
Potorous_tridactylus         AGAAAAGAAT TTGGCAAAAG AAATACCATC TGGTTCTATG GAAAGGCCAC
Setonix_brachyurus           AGAAAAGAAT TTGGCAAAAG AAACACCATC TGGGTCTATG GAAAGGCCAC
Thylogale_stigmatica         AGAAAAGAAT TTGGCAAAAG AAACACCATC TGGTTCTATG GAAAGGCCAC
Didelphis_marsupialis        .......... .......... .......... .......... ..........
Lasiorhinus_latifrons        .......... .......... .......... .......... ..........
Petaurus_breviceps           .......... .......... .......... .......... ..........
Phascolarctos_cinereus       .......... .......... .......... .......... ..........
Pseudocheirus_peregrinus     .......... .......... .......... .......... ..........
Spilocuscus_maculatus        .......... .......... .......... .......... ..........
Trichosurus_vulpecula        .......... .......... .......... .......... ..........
Monodelphis_domestica        .......... .......... .......... .......... ..........

                             1301                                              1350
Aepyprymnus_rufescens        TACCGGGAAC ACTAACATTG CGGAAGCCGT TGCACACACG GTGCCTTTAT
Dendrolagus_goodfellowi      TTCTGGGAAT AYTAACATTG TGGAAGCCAT TGCACACTCA GTGCCCTTAT
Dendrolagus_matschiei        TTCTGGGAAT ACTAACATTG TGGAAGCCAT TGCACACTCA GTGCCCTTAT
Hypsiprymnodon_moschatus     .......... .......... .......... .......... ..........
Lagostrophus_fasciatus       TACTGGGAAC AGTAACATTG CGGAAGTCGT TGCACACACG GTGCCCTTAT
Macropus_eugenii             TACTGGGAAC ACTAACATTG TGGAAGCCGT TGCACACACG GTGCCCTTCT
Macropus_parma               TACTGGGAAC ACTAACATTG TGGAAGCCAT TGCACACATG GTGCCCTTCT
Macropus_robustus            TACTGGGAAC ACTAACATTG TGGAAGCCGT TGCACACCCG GTGCCCTTCT
Macropus_rufogriseus         TACTGAGA.. ACTAACATTG TGGAAGCCGT TGCACACACG GTGCCCTTCT
```

```
Macropus_rufus            TACTGGGAAC ACTAACATTG TGGAAGCCAT TGCACACCCG GTGCCCTTCT
Macropus_giganteus        TACTGGGAAC ACTAACATTG TGGAAGCCGT TGCACACATG GTGCCCTTCT
Onychogalea_unguifera     TACTGGGAAC ACTAACATTG TGGAAGCCGT TGCACACACG GTGCCCTTCT
Petrogale_lateralis       TACTGGGAAT ACTAACATTG CAGAAGCCAT TGCACACTCG GTGCCCTTAT
Potorous_tridactylus      TACTGGGAAC ACTAACATTG CGGAAGCCAA TGCACACACG GTGCCCTTAT
Setonix_brachyurus        TATTGGGAAC GCTAACATTG TGGAAGCCGT TGCACACATG GTGCCCTTCT
Thylogale_stigmatica      TACTGGGAAT AGTAACATTG CAGAAGCCAT TGCACACTCA GTGCCGTTAT
Didelphis_marsupialis     .......... .......... .......... .......... ..........
Lasiorhinus_latifrons     .......... .......... .......... .......... ..........
Petaurus_breviceps        .......... .......... .......... .......... ..........
Phascolarctos_cinereus    .......... .......... .......... .......... ..........
Pseudocheirus_peregrinus  .......... .......... .......... .......... ..........
Spilocuscus_maculatus     .......... .......... .......... .......... ..........
Trichosurus_vulpecula     .......... .......... .......... .......... ..........
Monodelphis_domestica     .......... .......... .......... .......... ..........

                          1351                                                1400
Aepyprymnus_rufescens     ACGGGTATGT GAATTGGGCT AATGAGAACT TTCCATTCAA TGACTGTGTG
Dendrolagus_goodfellowi   ATGGGCATGT GAATTGGACT AATGAGAACT TTCCATTCAG TGATTGTGTG
Dendrolagus_matschiei     ATGGGCATGT GAATTGGACT AATGAGAACT TTCCATTCAG TGATTGTGTG
Hypsiprymnodon_moschatus  .......... .......... .......... .......... ..........
Lagostrophus_fasciatus    CAGGGTATGT GAATTGGATT TATGAGAACT TTCCATTCAG TAACTGTGTG
Macropus_eugenii          ACGGGTATGT GAATTGGACT AATGAGAACT TTCCATTCAG TGATTGTGTG
Macropus_parma            ATGGGTATGT GAATTGGACT AATGAGAACT TTCCATTCAA TGATTGTGTG
Macropus_robustus         ACGGGTATGT GAATTGGACT AATGAGAACT TTCCATTCAA TGATTGTGTG
Macropus_rufogriseus      ACGGGTATGT GAATTGGACT AATGAGAACT TTCCATTCAA TGATTGTGTG
Macropus_rufus            ACGGGTATGT GAATTGGACT AATGAGAACT TTCCATTCAA TGATTGTGTG
Macropus_giganteus        ACAGGTATGT GAATTGGACT AATGAGAACT TTCCATTCAA TGATTGTGTG
Onychogalea_unguifera     ACGGATATGT GAATTGGACC AATGAGAACT TTCCATTCAA TGATTATGTG
Petrogale_lateralis       ATGGGTATGT GAATTGGACA AATGAGAACT TTCCATTCAA TGATTGTGTG
Potorous_tridactylus      ACGGGAACGT GAATTGGACT AATGAGAACT TTCCATTCAG TCACTGTGTG
Setonix_brachyurus        ACAGGTATGT GAATTGGACT AATGAGAACT TTCCATTCAA TGATTGTGTG
Thylogale_stigmatica      ATGGGTATGT GAATTGGACT AATGAGACCT TTCCATTCAA TGATTGTGTG
Didelphis_marsupialis     .......... .......... .......... .......... ..........
Lasiorhinus_latifrons     .......... .......... .......... .......... ..........
Petaurus_breviceps        .......... .......... .......... .......... ..........
Phascolarctos_cinereus    .......... .......... .......... .......... ..........
Pseudocheirus_peregrinus  .......... .......... .......... .......... ..........
Spilocuscus_maculatus     .......... .......... .......... .......... ..........
Trichosurus_vulpecula     .......... .......... .......... .......... ..........
Monodelphis_domestica     .......... .......... .......... .......... ..........

                          1401                                                1450
Aepyprymnus_rufescens     GATAAAATAC TCATCTGGTG GGAGGAAGGT GAGATTACCT CTACGGTGGT
Dendrolagus_goodfellowi   GATAAAATGC TCATCTGGTG GGAGGAAGGT GAGATTACCT CTATGGTGGT
Dendrolagus_matschiei     GATAAAATGC TCATCTGGTG GGAGGAAGGT GAGATTACCT CTATGGTGGT
Hypsiprymnodon_moschatus  .......... .......... .......... .......... ..........
Lagostrophus_fasciatus    GATAAAATGC TCATTTGGTG GGAGGAAGGC GAGATTACCC CTAAGGTGAC
Macropus_eugenii          GATAAAATGC TCATCTGGTG GGAGGAAGGC AAGATTACCT CTATGGTGGT
Macropus_parma            GATAAAATGC TCATCTGGTG GGAGGAAGGC AAGATTACCT CTATGGTGGT
Macropus_robustus         GATAAAATGC TCATCTGGTG GGAGGAAGGC AAGATTACCT CTATGGTGGT
Macropus_rufogriseus      GATAAAATGC TCATCTGGTG GGAGGAAGGC AAGATTACCT CTATGGTGGT
Macropus_rufus            GATAAAATGC TCATCTGGTG GGAGGAAGGC AAGATTACCT CTATGGTGGT
Macropus_giganteus        GATAAAATGC TCATCTGGTG GGAGGAAGGC AAGATTACCT CTATGGTGGT
Onychogalea_unguifera     GATAAAATGC TCATCTCGTG GGAGCAAGGC AAGATTACCT TTATGGTGGT
Petrogale_lateralis       GATAAAATGC TCATCTGGTG GGAGGAAGGA GAGATTACCT CTATGGTGGT
Potorous_tridactylus      GATAAAATGC TCATCTGGTG GGAGGAAGGC GAGATTACCT CTAAGGTAGT
Setonix_brachyurus        GATAAAATGC TCATCTGGTG GGAGGAAGGC AAGATTACCT CTATGGTGGT
Thylogale_stigmatica      GATAAAATGC TCATCTGGTG GGAGGAAGGC GAGATTGCCT CTATGGTGGT
Didelphis_marsupialis     .......... .......... .......... .......... ..........
Lasiorhinus_latifrons     .......... .......... .......... .......... ..........
Petaurus_breviceps        .......... .......... .......... .......... ..........
Phascolarctos_cinereus    .......... .......... .......... .......... ..........
```

```
Pseudocheirus_peregrinus .......... .......... .......... .......... ..........
Spilocuscus_maculatus    .......... .......... .......... .......... ..........
Trichosurus_vulpecula    .......... .......... .......... .......... ..........
Monodelphis_domestica    .......... .......... .......... .......... ..........

                         1451                                               1500
Aepyprymnus_rufescens    TGAGACAGCT AAAGCCA... ..TCCTTGGA GGAGCTAAAG TTCAGGTGGA
Dendrolagus_goodfellowi  TGAGACAGCT AAAGCCA... ..TCCTTGAA GGAGCTAAAG TTCAGGTGGA
Dendrolagus_matschiei    TGAGACAGCT AAAGCCA... ..TCCTTGAA GGAGCTAAAG TTCAGGTGGA
Hypsiprymnodon_moschatus .......... .......... .......... .......... ..........
Lagostrophus_fasciatus   TGAGAAAGCT AAAGCCA... ..TCCTTGGA GGAGCTAAAG TTCCAGTGGA
Macropus_eugenii         TGAGACAGCT AAAGCCA... ..TCCTTGGA GGAGCTAAAG TTCAGGTGGA
Macropus_parma           TGAGACAGCT AAAGCCA... ..TCCTCAGA GGAGCTAAAG TTCAGGTGGA
Macropus_robustus        TGAGACAGCT AAAGCCA... ..TCCTTGGA GGAGCTAAAG TTCAGGTGGA
Macropus_rufogriseus     TGAGACAGCT AAAGCCA... ..TCCTCAGA GGAGCTAAAG TTCAGGTGGA
Macropus_rufus           TTAGACAGCT AAAGCCA... ..TCCTTGGA GGAGCTAAAG TTCAGGTGGA
Macropus_giganteus       TGAGACAGCT AAAGCCA... ..TCCTCGGA GGAGCTAAAG TTCAGGTGGA
Onychogalea_unguifera    TGAGACAGCT AAAGCCA... ..TCCTCGGA GGAGCTAAAG TTCAGGTGGA
Petrogale_lateralis      TGAGACAGCT AAAGCCA... ..TCCTTGAA GGWGCTAAAG TTCAGGTGGA
Potorous_tridactylus     AGAGACAGTT AAAGCGATCC TCTCCTCAGA GGAGCTAAAG TTCAAGTGGA
Setonix_brachyurus       TGAGACAGCT AAAGCCA... ..TCCTCGGA GGAGCTAAAG TTCAGGTGGA
Thylogale_stigmatica     TGAGACAGCT AAAGCCA... ..TCCTTGAA GGAGCTAAAG TTCAGGTGGA
Didelphis_marsupialis    .......... .......... .......... .......... ..........
Lasiorhinus_latifrons    .......... .......... .......... .......... ..........
Petaurus_breviceps       .......... .......... .......... .......... ..........
Phascolarctos_cinereus   .......... .......... .......... .......... ..........
Pseudocheirus_peregrinus .......... .......... .......... .......... ..........
Spilocuscus_maculatus    .......... .......... .......... .......... ..........
Trichosurus_vulpecula    .......... .......... .......... .......... ..........
Monodelphis_domestica    .......... .......... .......... .......... ..........

                         1501                                               1550
Aepyprymnus_rufescens    CCAGGAATCT CAGTCCTCTG TTCAAATTGA TTGTACTCCA GTCATC...A
Dendrolagus_goodfellowi  CCAGGAGTGT AAGTCCTCTC TTCAAATTGA TTGTACTCCA GTYATCATCA
Dendrolagus_matschiei    CCAGGAGTGT AAGTCCTCTC TTCAAATTGA TTGTACTCCA GTCATCATCA
Hypsiprymnodon_moschatus .......... .......... .......... .......... ..........
Lagostrophus_fasciatus   CCAGGAATGT AAGTCCTCTG TTCAAACTGA TTGTACTCCA GTCATCATCA
Macropus_eugenii         CCAGGAATGT AAGTCCTCTG TTCAAATTGA TTGTACTCCA GTCATCATCA
Macropus_parma           CCAGGAATGT AAGTCCTCTG TTCAAATTGA TTGTACTCCA GTCATCATTA
Macropus_robustus        CCAGGAATGT AAGTCCTCTG TTCAAATTGA TTGTGCTCCA GTCATCATCA
Macropus_rufogriseus     CCAGGAATGT AAGTCCTCTG TTCAAATTGA TTGTACTCCA GTCATCATCA
Macropus_rufus           CCAGGAATGT AAGTCCTCTG TTCAAATTGA TTGTACTCCA GTCATCATCA
Macropus_giganteus       CCAGGAATGT AAGTCCTCTG TTCAAATTGA TTGTACTCCA GTCTTCATTA
Onychogalea_unguifera    CCAGGAATGT AAGTCCTCTG TTCAAATTCA TTGTACTCCA GTCATAATTA
Petrogale_lateralis      CCAGGAGTGT AAGTCCTCTC TTCAAATTGA TTGTACTCCA GTCATCATCA
Potorous_tridactylus     CTAGGAATGT AAGTCCTCTG TTCAAATTGA TTGTACTCCA GTCATTATCA
Setonix_brachyurus       CCAGGAATGT AAGTCCTCTG TTCAAATTGA TAGTACTCCA GTCATCATCA
Thylogale_stigmatica     CCAGGAGTGT AAGGCCTCTC TTCAAATTGA TTGTGCTCCA GTCATCATCA
Didelphis_marsupialis    .......... .......... .......... .......... ..........
Lasiorhinus_latifrons    .......... .......... .......... .......... ..........
Petaurus_breviceps       .......... .......... .......... .......... ..........
Phascolarctos_cinereus   .......... .......... .......... .......... ..........
Pseudocheirus_peregrinus .......... .......... .......... .......... ..........
Spilocuscus_maculatus    .......... .......... .......... .......... ..........
Trichosurus_vulpecula    .......... .......... .......... .......... ..........
Monodelphis_domestica    .......... .......... .......... .......... ..........

                         1551                                               1600
Aepyprymnus_rufescens    CCTCCAA... .......... .......... .......... ..........
Dendrolagus_goodfellowi  CCTCCAACAC CAACATGTAC TACATGGTGG ACSAGAACAC TATGATCTTT
Dendrolagus_matschiei    CCTCCAACAT CAACATGTAC TACATGGTGG ACCAGAACAC TATGATCTTT
Hypsiprymnodon_moschatus .......... .......... .......... .......... ..........
Lagostrophus_fasciatus   CCTCCAACAC CGACATGCGC TACATGGTGG ACGGGAACAC TATGATCTTT
```

```
Macropus_eugenii          CCTCCAACAC CAACATGTGC TACATGGTGG ATCGGAACAC TATGATCTTT
Macropus_parma            CCTCCAGCAC CAACATGTGC TACGAGGTGG ATCAGAGCAC TATGATCTTT
Macropus_robustus         CCTCCAGCAC CGACATGTGC TACGTGGTGG ATCAGAACAC TATGATCTTT
Macropus_rufogriseus      CCTCCAACAC CGACATGTGC TACGTGGTGG ATCGGAACAC TATGATCTTT
Macropus_rufus            CCTCCAGCAC CGACATGTGC TACGTGGTGG ATCAGAACAC TATGATCTTT
Macropus_giganteus        CCTCCAGCAC CAACATGTGC TACGAGGTAG ATCAGAACAC TATGATCTTC
Onychogalea_unguifera     CCTCCAACAC CAACATGTGC TATGTGGTGG ATCAGAACAC TATGATCTTT
Petrogale_lateralis       CCTCCAACAC CAACATGTGC TACATGGTGG ACTGGAACAC TATGATCTTT
Potorous_tridactylus      CCTCCAA... .......... .......... .......... ..........
Setonix_brachyurus        CCTCCAACAC TGACATGTGC TACGTGGTGG ATCGGAACAC TATGATCTTT
Thylogale_stigmatica      CCTCCAACAC CAACATGTGC TACATGGTGG ACCGGAACAC TATGATCTTT
Didelphis_marsupialis     .......... .......... .......... .......... ..........
Lasiorhinus_latifrons     .......... .......... .......... .......... ..........
Petaurus_breviceps        .......... .......... .......... .......... ..........
Phascolarctos_cinereus    .......... .......... .......... .......... ..........
Pseudocheirus_peregrinus .......... .......... .......... .......... ..........
Spilocuscus_maculatus     .......... .......... .......... .......... ..........
Trichosurus_vulpecula     .......... .......... .......... .......... ..........
Monodelphis_domestica     .......... .......... .......... .......... ..........

                          1601                                            1650
Aepyprymnus_rufescens     .......... .......... .......... .......... ..........
Dendrolagus_goodfellowi   GAACACAAGC AGTCATTACA AGACCACATG TTTCAATTCA TGCTCATGGA
Dendrolagus_matschiei     GAACACAAGC AGTCATTACA AGACCACATG TTTCAATTCA TGCTCATGGA
Hypsiprymnodon_moschatus .......... .......... .......... .......... ..........
Lagostrophus_fasciatus    GAACACAAGC AGTTGTTACA AGACCACATG TTTCAATTCA TGCTCATGGA
Macropus_eugenii          GAGCATAAGC AGTTGTTACA AGACCACATG TTTCAATTCA TGCTCGTGGA
Macropus_parma            GAGCACAAGC AGTTGTTACA AGACCACATG TTTCAATTCA TGCTTGTGGA
Macropus_robustus         GAGCACAAGC AGTTGTTACA AGACCACATG TTTCAATTCA TGCTTGTGGA
Macropus_rufogriseus      GAGCATAAGC AGTTGTTACA AGACCACATG TTTCAATTCA TGCTCATGGA
Macropus_rufus            GAGCACAAGC AGTTGTTGCA AGACCACATG TTTCAATTCA TGCTCATGGA
Macropus_giganteus        GAGCACAAGC AGTTGTTACA AGACCACATG TTTTAATTCA TGCTCATGGA
Onychogalea_unguifera     GAGCACAAGC AGTTGTTACA AGACCACATG TTTCAATTCA TGCTCGTGGA
Petrogale_lateralis       GAACACAAGG AGTCATTACA AGACCACATG TTTCAATTCA TGCTCATGGA
Potorous_tridactylus      .......... .......... .......... .......... ..........
Setonix_brachyurus        GAGCACAAGC AGTTGTTACA AGACCACATG TTTCAATTCA TGTTCGTGGA
Thylogale_stigmatica      GAACACAAGC AGTCATTACA AGACCAC..G TTTCAATTCA TGCTTGTGGA
Didelphis_marsupialis     .......... .......... .......... .......... ..........
Lasiorhinus_latifrons     .......... .......... .......... .......... ..........
Petaurus_breviceps        .......... .......... .......... .......... ..........
Phascolarctos_cinereus    .......... .......... .......... .......... ..........
Pseudocheirus_peregrinus .......... .......... .......... .......... ..........
Spilocuscus_maculatus     .......... .......... .......... .......... ..........
Trichosurus_vulpecula     .......... .......... .......... .......... ..........
Monodelphis_domestica     .......... .......... .......... .......... ..........

                          1651                                            1700
Aepyprymnus_rufescens     .......... .......... .......... .......... ..........
Dendrolagus_goodfellowi   GAGACTTTCT GATGACTTTG GAAAAGTGAC AAAGGAGGAG GTGTGTCAGT
Dendrolagus_matschiei     GAGACTTTCT GATGACTTTG GAAAAGTGAC AAAGGAGGAG GTGTGTCAGT
Hypsiprymnodon_moschatus .......... .......... .......... .......... ..........
Lagostrophus_fasciatus    GAGGCTTCCT CATGACTTTG GCAAGGTGAC AAAGGAGGAA GTGAGTTAGT
Macropus_eugenii          GAGACTTCCT GATGACTTTG GCAAGGTGAC AAAGGAGGAG GTGCGTCAGT
Macropus_parma            GAGACTTCCT GATGACTTTG GCAAGGTGAC AAAGGAGGAG GTGCGTCAGT
Macropus_robustus         GAGACTTCCT GATGACTTTG GCAAGGTGAC AAAGGAGGAG GTGCATCAGT
Macropus_rufogriseus      GAGACTTCCT GATGACTTTG GCAAGGTGAC AAAGGAGGAG GTGCATCAGT
Macropus_rufus            GAGACTTCCT GATGACTTTG GCAAGGTGAC AAAGGAGGAG GTGCATCAGT
Macropus_giganteus        GAGACTTCCT GATGACTTTG GCAAGGTGAC AAAGGAGGAG GTGCAGCAGT
Onychogalea_unguifera     GAGACTTCCT GATGAC..TG GCAAGGTGAC AAAGGAGGGG GTGCGTCAGT
Petrogale_lateralis       GAGACTTTCT GATGACTTTG GAAAAGTGGC AAAGGAGGAG GTGTGTCAGT
Potorous_tridactylus      .......... .......... .......... .......... ..........
Setonix_brachyurus        GAGACTTCCT GATGACTTTG GCAAGGTGAC AAAGGAGGAG GTGCATCATT
Thylogale_stigmatica      GAGACTTTCT GATGACTTTG GAAAAGTGAC AAAGGAGGAG GGGTGTCAGT
```

```
Didelphis_marsupialis     .......... .......... .......... .......... ..........
Lasiorhinus_latifrons     .......... .......... .......... .......... ..........
Petaurus_breviceps        .......... .......... .......... .......... ..........
Phascolarctos_cinereus    .......... .......... .......... .......... ..........
Pseudocheirus_peregrinus  .......... .......... .......... .......... ..........
Spilocuscus_maculatus     .......... .......... .......... .......... ..........
Trichosurus_vulpecula     .......... .......... .......... .......... ..........
Monodelphis_domestica     .......... .......... .......... .......... ..........

                          1701                                               1750
Aepyprymnus_rufescens     .......... .......... .......... .......... ..........
Dendrolagus_goodfellowi   T.TTTTAAAT GGGCAGCTGT TAATCAAATT CCTCCTAAGC AGGAATTCAC
Dendrolagus_matschiei     T.TTTTAAAT GGGCAGCTGT TAATCAAATT CCTCCTAAGC AGGAATTCAC
Hypsiprymnodon_moschatus  .......... .......... .......... .......... ..........
Lagostrophus_fasciatus    TTTTTAAAAT GGGTAGCTGT TAATCAAATT CCCCCTAAGC AGGAATTCAC
Macropus_eugenii          TCTTTTAAAT GGGCAGCTGT TAATCAAATT CCCCCTGAGC AGGAATTCAC
Macropus_parma            TCTTTTAAAT GGGCAGCTGT TAATCAAATT CCCCCTGAGC AGGAATTCAC
Macropus_robustus         TCTTTTAAAT GGGCAGCTGT TAATCAAATT CCCCCTGAGC AGGAATTCAC
Macropus_rufogriseus      TCTTTTAAAT GGGCAGCTTT TAATCAAATT CCCCCCGAGC AGGAATTCAC
Macropus_rufus            TCTTTTAAAT GGGCAGCTGT TAATCAAATT CCCCCTGAGC AGGAATTCAC
Macropus_giganteus        TCTTTTAAAT GGGCAGCTGT TAATCAAATT CCCCCTGAGC AGGAATTCAC
Onychogalea_unguifera     TCTTTTAAAT GGGCAGCTGT TAATCAAATT CTGCCTGAGC AGGAATTCAT
Petrogale_lateralis       T.TTTTAAAT GGGCAGCTGT TAATCAAATT CTCCCCAAGC AGGAATTCAC
Potorous_tridactylus      .......... .......... .......... .......... ..........
Setonix_brachyurus        TCTTTTAAAT GGGCAGCTGT TAATCAAATT CCCCCTGAGT AGGGATTCAC
Thylogale_stigmatica      T.TTTTAAAT GGGCAACTGT TAATCAAATT CTCCCTAAGC AGGAATTCAC
Didelphis_marsupialis     .......... .......... .......... .......... ..........
Lasiorhinus_latifrons     .......... .......... .......... .......... ..........
Petaurus_breviceps        .......... .......... .......... .......... ..........
Phascolarctos_cinereus    .......... .......... .......... .......... ..........
Pseudocheirus_peregrinus  .......... .......... .......... .......... ..........
Spilocuscus_maculatus     .......... .......... .......... .......... ..........
Trichosurus_vulpecula     .......... .......... .......... .......... ..........
Monodelphis_domestica     .......... .......... .......... .......... ..........

                          1751                                               1800
Aepyprymnus_rufescens     .......... .......... .......... .......... ..........
Dendrolagus_goodfellowi   TATCAAGAAG ATTACATCAT CCATTAACTG TTACTATGAC CACAGGCGGA
Dendrolagus_matschiei     TATCAAGAAG ATTACATCAT CCATTAACTG TTACTATGAC CACAGGCGGA
Hypsiprymnodon_moschatus  .......... .......... .......... .......... ..........
Lagostrophus_fasciatus    TGTCAAGAAG ATTATGTCAT CCATTGACTG TTACTATGGC CACAAGCAGA
Macropus_eugenii          TTTCAAGAAG ATTATGTCAT CCATTGACTG TTATTATGAC CACAAGCAGA
Macropus_parma            TGTCAAGAAG ATTATGTCAT CCATTGACTG TTATTATGAC CACAAGCAGA
Macropus_robustus         TGTCAAGAAG ATTATGTCAT CCATTGACTG TTATTATGAC CACAAGCAGA
Macropus_rufogriseus      TGTCAAGAAG ATTATGTCAT CCATTGACTG TTATTATGAC CACAAGCAGA
Macropus_rufus            TGTCAAGAAG ATTATGTCAT CCATTGACTG TTATTATGAC CACAAGCAGA
Macropus_giganteus        TGTCAAGAAG ATTATGTCAT CCATTGACTG TTAGTGTGAC CACAAGCAGA
Onychogalea_unguifera     TGTCAAGAAG ATTATGTCAT CCATTGACTG TTATTATGAC CACAAGCAGA
Petrogale_lateralis       TATCAAGAAG ATTACATCAT CCATTGACTG TTACTATGAC CACAGGTGGA
Potorous_tridactylus      .......... .......... .......... .......... ..........
Setonix_brachyurus        TGTCAAGAAG ATTATGTCAT CCATTGACTG TTATTATGAC CACAAGCAGA
Thylogale_stigmatica      TATCAAGAAG ATTACATCAT CCATTGACTG TTACTATGAC CACAGGTGGA
Didelphis_marsupialis     .......... .......... .......... .......... ..........
Lasiorhinus_latifrons     .......... .......... .......... .......... ..........
Petaurus_breviceps        .......... .......... .......... .......... ..........
Phascolarctos_cinereus    .......... .......... .......... .......... ..........
Pseudocheirus_peregrinus  .......... .......... .......... .......... ..........
Spilocuscus_maculatus     .......... .......... .......... .......... ..........
Trichosurus_vulpecula     .......... .......... .......... .......... ..........
Monodelphis_domestica     .......... .......... .......... .......... ..........

                          1801                                               1850
Aepyprymnus_rufescens     .......... .......... .......... .......... ..........
```

```
Dendrolagus_goodfellowi  AATGGGAGGA GTATCCCTCG ACTTTCTGTC AAGGGGGCTA T.AAAAGGCC
Dendrolagus_matschiei    AATGGGAGGA GTATCCCTCG ACTTTCTGTC AAGGGGGCTA T.AAAAGGCC
Hypsiprymnodon_moschatus .......... .......... .......... .......... ..........
Lagostrophus_fasciatus   AATGGGAGGA GTATCCCTCG ACTTTCTGTC AAGGGGGCTA TAAAAAGGCT
Macropus_eugenii         AATGGGAGGA GTATCCCTCG ACTTTCTATC AAGGGGGCTA TAAAAAGGCC
Macropus_parma           AATGGGAGGA GTATCCCTCG ACTTTCTATC AAGGGGGCTA TAAAAAGGGT
Macropus_robustus        AATGGGAGGA GTATCCCTCG ACTTTCTATC AAGGGGGCTA TAAAAAGGCC
Macropus_rufogriseus     AATGGGAGGA GTATCCCTCG ACTTTCTATC AAGGGGGCTA TAAAAAGGGC
Macropus_rufus           AATGGGAGGA GTATCCCTCG ACTTTCTATC AAGGGGGCTA TAAAAAGGCC
Macropus_giganteus       AACGGGAGGA GTATCCCTCA ACTTTCTATC AAGGGGGCTA TAAAAAGGGC
Onychogalea_unguifera    AATGGGAGGA GTATCCCTCG ACTTTCTTAC AAGGGGGCTA TGAAAAGGGT
Petrogale_lateralis      AATGGGAGGA GTATCCCTCC ACTTTCTGTC AAGGGGGCTA T.AAAAGGCC
Potorous_tridactylus     .......... .......... .......... .......... ..........
Setonix_brachyurus       AATGGGAGGA GTATCCCTCA ACTTTCTATC AAGGGGGCTA TAAAAAGGCT
Thylogale_stigmatica     AATGGGAGAA GTATCCCTGG ACTTTCCGTC AAGGGGGCTA T.AAAAGGCC
Didelphis_marsupialis    .......... .......... .......... .......... ..........
Lasiorhinus_latifrons    .......... .......... .......... .......... ..........
Petaurus_breviceps       .......... .......... .......... .......... ..........
Phascolarctos_cinereus   .......... .......... .......... .......... ..........
Pseudocheirus_peregrinus .......... .......... .......... .......... ..........
Spilocuscus_maculatus    .......... .......... .......... .......... ..........
Trichosurus_vulpecula    .......... .......... .......... .......... ..........
Monodelphis_domestica    .......... .......... .......... .......... ..........

                         1851                                              1900
Aepyprymnus_rufescens    .......... .......... .......... .......... ..........
Dendrolagus_goodfellowi  AAGCCTCCCT CAAAAAAGTT CTTTCCATTC TGGGATTTGA AGAAAGTTGA
Dendrolagus_matschiei    AAGCCTCCCT CAAAAAAGTT CTTTCCATTC TGGGATTTGA AGAAAGTTGA
Hypsiprymnodon_moschatus .......... .......... .......... .......... ..........
Lagostrophus_fasciatus   GAGCCTCCCT TGAAAAAGTT CTTTCCATTC AGGGATCTGA AGAAAATTGA
Macropus_eugenii         GAGCCTCCCT CGAAAAAGTT CTTTCCATTT CGGGATTTGA AGAAAATTGA
Macropus_parma           GAGCCTCCCT CGAAAAAGTT CTTTCCATTT CTGGATTTGA AGAAAATTGA
Macropus_robustus        GAGCCTCCCT CGAAAAAGTT CTTTCCATTT CAGGATTTGA AGAAAATTGA
Macropus_rufogriseus     GAGCCTCCCT CAAAAAAGTT CTTTCCATTT CAGGATTTGA AGAAAATTGA
Macropus_rufus           GAGCCTCCCT TGAAAAAGTT CTTTCCATTT TGGGATTTGA AGAAAATTGA
Macropus_giganteus       GAGCCTCCCT CAAAAAAGTT CTTTCCATTT CGGCATTTGA AGAAAATTGA
Onychogalea_unguifera    GAGCCTCCCT CGAAAAAGTT CTTTCCATTT TGGGATTTGA AGAAAATTGA
Petrogale_lateralis      GAGCCTCCCT CAAAAAAGTT CTTTCCGTTC TGGGATTTGA AGAAAGTTGA
Potorous_tridactylus     .......... .......... .......... .......... ..........
Setonix_brachyurus       GAGCCTCCCT TGAAAAAGTT CTTTCCATTT CGGGATTTGA AGAAAATTGA
Thylogale_stigmatica     GAGCCTCCCT CAAAAAAGTT CTTTCCATTC TGGGATTTGA AGAAAGTTGA
Didelphis_marsupialis    .......... .......... .......... .......... ..........
Lasiorhinus_latifrons    .......... .......... .......... .......... ..........
Petaurus_breviceps       .......... .......... .......... .......... ..........
Phascolarctos_cinereus   .......... .......... .......... .......... ..........
Pseudocheirus_peregrinus .......... .......... .......... .......... ..........
Spilocuscus_maculatus    .......... .......... .......... .......... ..........
Trichosurus_vulpecula    .......... .......... .......... .......... ..........
Monodelphis_domestica    .......... .......... .......... .......... ..........

                         1901                                              1950
Aepyprymnus_rufescens    .......... .......... .......... .......... ..........
Dendrolagus_goodfellowi  GGTCATTGAG CAGAGAGCCC CCACAGTGGA TTYGGACTTC AAGAATCTCA
Dendrolagus_matschiei    GGTCATTGAG CAGAGAGCCC CCACAGTGGA TTCGGACTTC AAGAATCTCA
Hypsiprymnodon_moschatus .......... .......... .......... .......... ..........
Lagostrophus_fasciatus   GGTCATCAAG CAGAGAGCCC CCACAGTGGA ATCGGACTTT GAGAATCTCA
Macropus_eugenii         AGTCATCGAG CAGAGAGTCC CCACAGTGGA ATTGAACTTT GAGAATCTCA
Macropus_parma           GGTCATCAAG CAGAGAGCCC CCACGGTGGA ATTGAACTTT GGGAATCTCA
Macropus_robustus        AGTCATCGAG CAGAGAGCCC CCACAGTGGA ATTGAACTTT GAGAATCTCA
Macropus_rufogriseus     GGTCATTAAG CAGAGAGCCC CCACAGTGGA ATTGAACTTT GAGAATCTCA
Macropus_rufus           GGTCATCGAG CAGAAAGCCC CCACAGTGGA ATTGAACTTT GAGAATCTCA
Macropus_giganteus       GGTCATCGAG CAGAGAGCCC CCACAATGGA ATTGAACTTT GAAAATCTCA
Onychogalea_unguifera    GGTCATTGAG CAGAGAGCCC CCACAGTGGA ACTGAACTTT GAGAATCTCA
```

```
Petrogale_lateralis       GGTCATTGAG CAGAGAGCCC CCACAGTGGA TTCGGACTTC AAGAATCTCA
Potorous_tridactylus      .......... .......... .......... .......... ..........
Setonix_brachyurus        GGTCATCGAG CAGAGAGCCC CCACAGTGGA ATTGAACTTT GAGAATCTCA
Thylogale_stigmatica      GGTCATTGAG CAGAGAGCCC CCACAGTGGA TTCAGACTTC AAGAATCTCA
Didelphis_marsupialis     .......... .......... .......... .......... ..........
Lasiorhinus_latifrons     .......... .......... .......... .......... ..........
Petaurus_breviceps        .......... .......... .......... .......... ..........
Phascolarctos_cinereus    .......... .......... .......... .......... ..........
Pseudocheirus_peregrinus .......... .......... .......... .......... ..........
Spilocuscus_maculatus     .......... .......... .......... .......... ..........
Trichosurus_vulpecula     .......... .......... .......... .......... ..........
Monodelphis_domestica     .......... .......... .......... .......... ..........

                          1951                                               2000
Aepyprymnus_rufescens     .......... .......... .......... .......... ..........
Dendrolagus_goodfellowi   AAAGGTTTGG TCTCAATGTA ATTCTTGCTT CT........ .GCTTTGGTT
Dendrolagus_matschiei     AAAGGTTTGG TCTCAATGTA ATTCTTGCTT CT........ .GCTTTGGTT
Hypsiprymnodon_moschatus .......... .......... .......... .......... ..........
Lagostrophus_fasciatus    AAAGGTTTGG TGTCAATGTA ATTCCCACTT CTGATCCTGT TGCTTTGGAT
Macropus_eugenii          AAAGGTTTGG TCTCAATGTA ATTCCCACTT CTGATCCTAT TGCTTTGGAT
Macropus_parma            AAAGGTTTGG TCTCAATGTA ATTCCTGCTT CTGATCCTAT TGCTTTGGAT
Macropus_robustus         AAAGGTTTGG TCTCAATGTA ATTCCTGCTT CTGATCCTAT CGCTTTGGAT
Macropus_rufogriseus      AAAGGTTTGG TCTCAATGTA ATTCCTGCTT CTGATCCTAT TGCTTTGGAT
Macropus_rufus            AAAGGTTTGG TCTCAATGTA ATTCCTGCTT CTGATCCTAT TGCTTTGGAT
Macropus_giganteus        AAAGGTTTGG TCTCAATGTA ATTCCCGCTT CTGATCCTAT TGCTTTGGAT
Onychogalea_unguifera     AAAGGTTTGG TCTCAATGTA ATTCCCGCTT CTGATCCTAT TGCTTTGGAT
Petrogale_lateralis       AAAGGTTTGG TCTCAATGTA ATTCTTGCTT CT........ .GCTTTGGTT
Potorous_tridactylus      .......... .......... .......... .......... ..........
Setonix_brachyurus        AAAGGTTTGG TCTCAATGTA ATTCCCGCTT CTGATCCTAT TGCTTTGGAT
Thylogale_stigmatica      AAAGGTTTGG TCTCAATGTA ATTCTTGCTT CT........ .GCTTTGGTT
Didelphis_marsupialis     .......... .......... .......... .......... ..........
Lasiorhinus_latifrons     .......... .......... .......... .......... ..........
Petaurus_breviceps        .......... .......... .......... .......... ..........
Phascolarctos_cinereus    .......... .......... .......... .......... ..........
Pseudocheirus_peregrinus .......... .......... .......... .......... ..........
Spilocuscus_maculatus     .......... .......... .......... .......... ..........
Trichosurus_vulpecula     .......... .......... .......... .......... ..........
Monodelphis_domestica     .......... .......... .......... .......... ..........

                          2001                                               2050
Aepyprymnus_rufescens     .......... .......... .......... .......... ..........
Dendrolagus_goodfellowi   GATTGTCAAG ATGAGCAAT. AAATCCGTAG ATGTCTTTTT TTGGAAAAAT
Dendrolagus_matschiei     GATTGTCAAG ATGAGCAAT. AAATCCGTAG ATGTCTTTTT TTGGAAAAAT
Hypsiprymnodon_moschatus .......... .......... .......... .......... ..........
Lagostrophus_fasciatus    GACTGTCAGG ATGAGCAGT. AAATCCGTAG ATGTCTTTTT T.GGAGAAAT
Macropus_eugenii          GACTGTCAGG ATGAGGAAT. AAATCCCTAG ATGTCTTTTT T.GGAGAAAT
Macropus_parma            GACTGTCAGG ATGAGGAAT. AAATCCCTAG ATGTCTTTTT T.GGAGAAAT
Macropus_robustus         GACTGTCAGG ATGAGGAAT. AAATCCCTAG ATGTCTTTTT T.GGAGAAAT
Macropus_rufogriseus      GACTGTCAGG ATGAGGAATA AAATCCCTAG ATGTCTTTTT T.GGAGAAAT
Macropus_rufus            GACTGTCAGG ATGAGGAAT. AAATCCCTAG ATGTCTTTTT T.GGAGAAAT
Macropus_giganteus        GACTGTCAGG ATGAGGAAT. AAATCCCTAG ATGTCTTTTT T.GGAGAAAT
Onychogalea_unguifera     GACTGTCAGG ATGAGGAAT. AAACCCCTAG ATGTCTTTTT T.GTAGAAAT
Petrogale_lateralis       GACTGTCAAG ATGAGCAAT. AAATCCATAG ATGTCTTTTT TCGGAAAAAT
Potorous_tridactylus      .......... .......... .......... .......... ..........
Setonix_brachyurus        GACTGTCAGG ATGAGGAAT. AAATCCCTAG ATGTCTTTTT T.GGAGAAAT
Thylogale_stigmatica      GACTGTCAAG ATGAGCAAT. AAATCCGTAG ATGTCTTTTT TTGGAAAAAT
Didelphis_marsupialis     .......... .......... .......... .......... ..........
Lasiorhinus_latifrons     .......... .......... .......... .......... ..........
Petaurus_breviceps        .......... .......... .......... .......... ..........
Phascolarctos_cinereus    .......... .......... .......... .......... ..........
Pseudocheirus_peregrinus .......... .......... .......... .......... ..........
Spilocuscus_maculatus     .......... .......... .......... .......... ..........
Trichosurus_vulpecula     .......... .......... .......... .......... ..........
```

```
Monodelphis_domestica     .......... .......... .......... .......... ..........

                          2051                                               2100
Aepyprymnus_rufescens     .......... .......... .......... .......... ..........
Dendrolagus_goodfellowi   TTGAGGACTG ATAGGAAAAG TTAGCTGCTA CTTGGAGACA CC.TTGAATC
Dendrolagus_matschiei     TTGAGGACTG ATAGGAAAAG TTAGCTGCTA CTTGGAGACA CC.TTGAATC
Hypsiprymnodon_moschatus  .......... .......... .......... .......... ..........
Lagostrophus_fasciatus    TTGAGGAATG GTATGAAAAG TCAGCTGCTA CTTGGAGATG CC.TTGAAGT
Macropus_eugenii          TTGAGGACTG GTACAAAAAG TCAGCTGCTA TTTGGAGACA CC.TTGAAGC
Macropus_parma            TTGAGGACTG GTACAAAAAG TCAGCTGCTA TTTGGAGACA CCTTTGAAGC
Macropus_robustus         TTGAGGACTG ATACAAAAAG TCAGCTGCTA TTTGGAGACA C..TTGAAGC
Macropus_rufogriseus      TTGAGGACTG GTACAAAAAG TCAGCTGCTA TTTGGAGACA CC.TTGAAGA
Macropus_rufus            TTGAGGACTG GTACAAAAAG TCAGCTGCTA TTTGGAGACA CC.TTGAAGC
Macropus_giganteus        TTGAGGACTG GTACAAAAAG TCAGCTGCTA TTTGGAGACA CC.TTGAAGC
Onychogalea_unguifera     TTGAGGACTG GTACAAAAAG TCAGCTGCTA TTTGGAAACA CC.TTGAAGC
Petrogale_lateralis       TTGAGGACTG ATAGGAAAAG TTAGCTGCTA CTTGGAGAAA CC.TTGAATC
Potorous_tridactylus      .......... .......... .......... .......... ..........
Setonix_brachyurus        TTGAGGACTG GTACAAAAAG TCAGCTGCTA TTTGGAGACA CC.TTGAAGC
Thylogale_stigmatica      TTGTGGACTG ATAGGAAAAG TTAGCTGCTA CTTGGAGACA CC.TTGAATC
Didelphis_marsupialis     .......... .......... .......... .......... ..........
Lasiorhinus_latifrons     .......... .......... .......... .......... ..........
Petaurus_breviceps        .......... .......... .......... .......... ..........
Phascolarctos_cinereus    .......... .......... .......... .......... ..........
Pseudocheirus_peregrinus  .......... .......... .......... .......... ..........
Spilocuscus_maculatus     .......... .......... .......... .......... ..........
Trichosurus_vulpecula     .......... .......... .......... .......... ..........
Monodelphis_domestica     .......... .......... .......... .......... ..........

                          2101                                               2150
Aepyprymnus_rufescens     .......... .......... .......... .......... ..........
Dendrolagus_goodfellowi   TGGCCCACCT CATCCTAAAG CTAATCAACA ACATCAAAAT GACTCTCATG
Dendrolagus_matschiei     TGGCCCACCT CATCCTAAAG CTAATCAACA ACATCAAAAT GACTCTCATG
Hypsiprymnodon_moschatus  .......... .......... .......... .......... ..........
Lagostrophus_fasciatus    TGGCCCACCT CATCCTAAAG CTAATCAACA ACATCAAGAC TACTCTCATG
Macropus_eugenii          TGGCCCACCT CATCCTAAAG CTAATCAACA ACATCAAGAT GACTCTCATG
Macropus_parma            TGGCCCACCT CATCCTAAAG CTAATCAACA ACATCAAGAT GACTTTCATG
Macropus_robustus         TGGCCCACCT CATCCTAAAG CTAATCAACA ACATCAAGAT GACTCTCATG
Macropus_rufogriseus      TGGCCCACCT CATCCTAAAG CTAATCAACA ACATCAAGAT GACTCTCATG
Macropus_rufus            TGGCCCACCT CATCCTAAAG CTAATCAACA ACATCAAGAT GACTCTCATG
Macropus_giganteus        TGGCCCACCT CATCCTAAAG CTAATCAACA ACATCAAGAT GATTCTCATG
Onychogalea_unguifera     TGGCCCACYT CATCCTAAAG CTAATCAACA ACATCAAGAT GACTCTCATG
Petrogale_lateralis       TGGCCCACCT CATCCTAAAG CTAATCAACA ACATCAGGAT GACTCTCATG
Potorous_tridactylus      .......... .......... .......... .......... ..........
Setonix_brachyurus        TGGCCCACCT CATCCTAAAG CTAATCAACA ACATCAAGAT GACTCTCATG
Thylogale_stigmatica      TGGCCCACCT CATACTAAAG CTAATCAACA ACATCAAGAT GACTCTCATG
Didelphis_marsupialis     .......... .......... .......... .......... ..........
Lasiorhinus_latifrons     .......... .......... .......... .......... ..........
Petaurus_breviceps        .......... .......... .......... .......... ..........
Phascolarctos_cinereus    .......... .......... .......... .......... ..........
Pseudocheirus_peregrinus  .......... .......... .......... .......... ..........
Spilocuscus_maculatus     .......... .......... .......... .......... ..........
Trichosurus_vulpecula     .......... .......... .......... .......... ..........
Monodelphis_domestica     .......... .......... .......... .......... ..........

                          2151                                               2200
Aepyprymnus_rufescens     .......... .......... .......... .......... ..........
Dendrolagus_goodfellowi   GACTGGTTCT GCCAGGCTAT AAGTATCTCA TTCCCTTTAA TGGTCTCTAT
Dendrolagus_matschiei     GACTGGTTCT GCCAGGCTAT AAGTATCTCA TTCCCTTTAA TGGTCTCTAT
Hypsiprymnodon_moschatus  .......... .......... .......... .......... ..........
Lagostrophus_fasciatus    GACTGGTTCT GCCAGGCTAT AAGTATCTCA TTCCCTTTAA TGGTCTCTAT
Macropus_eugenii          GACTGGTTCT GGCAGGCTAT AAGTATCTTA TTCCCTTTAA TGGTCTCTAT
Macropus_parma            GACTGGTTCT GCCAGGCTAT AAGTATTTTA TTCGCTTTAA TGGTCTCTAT
Macropus_robustus         GACTGGTTCT GCCAGGCTAT AAGTATCTTA TTCCCTTTAA TGGTCTCTAT
```

```
Macropus_rufogriseus      GACTGGTTTT TTCAGGCTAT AAGTATCTTA TTCCCTTTAA TGGTCTCCAT
Macropus_rufus            GACTGGTTCT GCCAGGCTAT AAGTATCTTA TTCCCTTTAA TGGTCTCTAT
Macropus_giganteus        GACTGGTTCT GCCAGGCTAT AAGCATCTTA TTCCCTTTAA TGGTCTCTAT
Onychogalea_unguifera     GACTGGTTCT GCCAGGCTAT AAGTATCTTA TTCCCTTTAA TGGTCTCTAT
Petrogale_lateralis       GACTGGTTCT GCCAGGCTAT AAGTATCTCA TTCCCTTTAA TGGTCTCTAT
Potorous_tridactylus      .......... .......... .......... .......... ..........
Setonix_brachyurus        GACTGGTTCT GCCAGGCTAT AAGTATCTTA TTCCCTTTAA TGATCTCTAT
Thylogale_stigmatica      GACTGGTTCT GCCAGGCTAT AAGTATCTCA TTCCCTTTAA TGGTCTCTAT
Didelphis_marsupialis     .......... .......... .......... .......... ..........
Lasiorhinus_latifrons     .......... .......... .......... .......... ..........
Petaurus_breviceps        .......... .......... .......... .......... ..........
Phascolarctos_cinereus    .......... .......... .......... .......... ..........
Pseudocheirus_peregrinus  .......... .......... .......... .......... ..........
Spilocuscus_maculatus     .......... .......... .......... .......... ..........
Trichosurus_vulpecula     .......... .......... .......... .......... ..........
Monodelphis_domestica     .......... .......... .......... .......... ..........

                          2201                                               2250
Aepyprymnus_rufescens     .......... .......... .......... .......... ..........
Dendrolagus_goodfellowi   AAGGGGGAGC CAGTTAATCA AGGGGACAAA GCCACACTGG AGCAAGAGAA
Dendrolagus_matschiei     AAGGGGGAGC CAGTTAATCA AGGGGACAAA GCCACACTGG AGCAAGAGAA
Hypsiprymnodon_moschatus  .......... .......... .......... .......... ..........
Lagostrophus_fasciatus    AAGGGGGAGC CAGTTAATCA AGCAGGCAAA GCCACTCTGG AACAAGAGAA
Macropus_eugenii          AAGGGGGAGC CAGTTAATTA AGCAGACAAA GCCACACTCG AACAAGAGAA
Macropus_parma            AAGGGGGAGC CAGTTAATCA AGCAGACAAA GCCACACTCG AACAAGAGAA
Macropus_robustus         AAGGGGGAGC CAGTTAATCA AGCAGACAAA GCCACACTCG AACAAGAGAA
Macropus_rufogriseus      AAGGGAGAGC TAGTTAATCA AGCAGACAAA GCCACACTTG AACAAGAGAA
Macropus_rufus            AAGGGGGAGC CAGTTAATCA AGCAGACAAA GCCACACTCG AACAACCGAA
Macropus_giganteus        AAGGGAGAGC CAGTTAATCA AGCAGACAAA GCCACACTTG AAGAAGAGAA
Onychogalea_unguifera     AAGGGGGAGC CAGTTAATCA AGCAGACAAA GCCACACTCA AACAAGAGAA
Petrogale_lateralis       AAGGGGGAGC CAGTTAATCA AGGGGACAAA GCCACACTGG AGCAAGAGAA
Potorous_tridactylus      .......... .......... .......... .......... ..........
Setonix_brachyurus        AAGGGGGAGC CAGTTAATCA AGCAGACAAA GCCACACTCA AACAAGAGAA
Thylogale_stigmatica      AAGGGGGAGC CAGTTAATCA AGGGGACAAA GCCACACTGG AGCAAGAGAA
Didelphis_marsupialis     .......... .......... .......... .......... ..........
Lasiorhinus_latifrons     .......... .......... .......... .......... ..........
Petaurus_breviceps        .......... .......... .......... .......... ..........
Phascolarctos_cinereus    .......... .......... .......... .......... ..........
Pseudocheirus_peregrinus  .......... .......... .......... .......... ..........
Spilocuscus_maculatus     .......... .......... .......... .......... ..........
Trichosurus_vulpecula     .......... .......... .......... .......... ..........
Monodelphis_domestica     .......... .......... .......... .......... ..........

                          2251                                               2300
Aepyprymnus_rufescens     .......... .......... .......... .......... ..........
Dendrolagus_goodfellowi   AGCTTACGAT CAATTCCTCA AAGAAGGGGA AAATCCTTAC CTCACCTACA
Dendrolagus_matschiei     AGCTTACAAT CAATTCCTCA AAGAAGGGGA AAATCCTTAC CTCACCTACA
Hypsiprymnodon_moschatus  .......... .......... .......... .......... ..........
Lagostrophus_fasciatus    ATCCTACGAT CAATTCCTCA AAGAAGGGGA AAATCCTTAC CTCACCTACA
Macropus_eugenii          AGCCTATGAT CAATTCCTCA AAGAAGGGGA AAATCCTTTC CTCACCTACA
Macropus_parma            AGCCTATGAT CAATTCCTCA AAGAAGGGGA AAATCCTTTC CTCACCTACA
Macropus_robustus         AGCCTATGAT CAATTACTCA AGGAAGGGGA AAATCCTTTC CTCACCTACA
Macropus_rufogriseus      AGCCTATGAT CAATTCCTCA AAG....... AAATCCTTTC CTCGCCTACA
Macropus_rufus            AGCCTATGAT CAATTCCTCA AAGAAGGGGA AAATCCTTTT CTCACCTACA
Macropus_giganteus        AGCCTATGAT CAATTCCTCA AAGAAGGGGA AAATCCTTTC CTCACCTACA
Onychogalea_unguifera     ARCCTATGAT CAATTCCTCA AAGAAGGGGA AAATCCTTTC CTCACCTACA
Petrogale_lateralis       AGCTTACGAT CAATTCCTCA AAGAAGGAGA AAATCCTGAC CTCACCTACA
Potorous_tridactylus      .......... .......... .......... .......... ..........
Setonix_brachyurus        AGCCTATGAT CAATTCCTCA AAGAAGGGGA AAATCCTTTC CTCACCTACA
Thylogale_stigmatica      AGCTTATGAT CAATTCCTCA AAGAAGGGGA AAATCCTTAC CTCACCTACA
Didelphis_marsupialis     .......... .......... .......... .......... ..........
Lasiorhinus_latifrons     .......... .......... .......... .......... ..........
Petaurus_breviceps        .......... .......... .......... .......... ..........
```

```
Phascolarctos_cinereus    .......... .......... .......... .......... ..........
Pseudocheirus_peregrinus .......... .......... .......... .......... ..........
Spilocuscus_maculatus     .......... .......... .......... .......... ..........
Trichosurus_vulpecula     .......... .......... .......... .......... ..........
Monodelphis_domestica     .......... .......... .......... .......... ..........

                          2301                                               2350
Aepyprymnus_rufescens     .......... .......... .......... .......... ..........
Dendrolagus_goodfellowi   ACCACACAGA CCAAGAGTTC CAGGARRACC TTTCGGAGGA CACTTTGTTT
Dendrolagus_matschiei     ACCACACAGA CCAAGAGTTC CAGG...ACC TTTCGGAGGA CACTTTGTTT
Hypsiprymnodon_moschatus .......... .......... .......... .......... ..........
Lagostrophus_fasciatus    AGCACACAGA CCCAGAGTTC CAGGAAAAAC TTTCAGAGGA CACTTCATTT
Macropus_eugenii          ACCACACAGA CCAAGAGTTC CAGGAAAAAC TTTCAGAGGG CACTTCATTT
Macropus_parma            ACCACAAAGA CCAAGAGTTC CAGGAAAAAC TTTCGGAGGG CACTTCGTTT
Macropus_robustus         ACCACACAGA CCAAGAGTTC CAGGAAAAAT TTTCGGAGGG CACTTCGTTT
Macropus_rufogriseus      ACCACACAGA CCAAGAGTTC CAGGAAAAAC TTTCGGAGGG CACTTCGTTT
Macropus_rufus            ACCACACAGA CCAAGAGTTC CAGGAAAAAC TTTCGGAGGG CACTTTGTTT
Macropus_giganteus        ACCACACAGA CCAAGAGTTC CAGGAAAAGC TTTCGGAGGG CACTTCGTTT
Onychogalea_unguifera     ACCACACAGA CCAAGAGTTC CAGGAAAAAC TTTCAGAGGG CACTTCGTTT
Petrogale_lateralis       ACCACACAGA CCAAGAGTTC CAGGAAAAAC TTTCGGAGGA CACTTTGTTT
Potorous_tridactylus      .......... .......... .......... .......... ..........
Setonix_brachyurus        ACCACACAGA CCACGAGTTC CAGGAAAAAC TTTCGGAGGA CACTTCGTTT
Thylogale_stigmatica      ACCACACGGA CCAAGAGTTC CAGGAAAAAC TTTTGGAGGA CACTTTGTTT
Didelphis_marsupialis     .......... .......... .......... .......... ..........
Lasiorhinus_latifrons     .......... .......... .......... .......... ..........
Petaurus_breviceps        .......... .......... .......... .......... ..........
Phascolarctos_cinereus    .......... .......... .......... .......... ..........
Pseudocheirus_peregrinus .......... .......... .......... .......... ..........
Spilocuscus_maculatus     .......... .......... .......... .......... ..........
Trichosurus_vulpecula     .......... .......... .......... .......... ..........
Monodelphis_domestica     .......... .......... .......... .......... ..........

                          2351                                               2400
Aepyprymnus_rufescens     .......... .......... .......... .......... ..........
Dendrolagus_goodfellowi   GGTGGTAACC TTGGCAAGGC AGTGTTTCAA GGAAAGAAAT GACTGCTTAA
Dendrolagus_matschiei     GGTGGTAACC TTGGCAAGGC AGTGTTTCAA GGAAAGAAAT GACTGCTTAA
Hypsiprymnodon_moschatus .......... .......... .......... .......... ..........
Lagostrophus_fasciatus    GGTGCTAACC TTGGCAAGGC AGTGCTTCAA GGAAAGAAAC CTCTGCTTAA
Macropus_eugenii          GGTGGTAACC TTGGCAAGGC AGTGTTTCAA GGAAAGAAAT GACTGCTTAA
Macropus_parma            GGTGGTAACC TTGGCAAGGC AGTGTTTCAA GGAAAGAAAT GACTGCTTAA
Macropus_robustus         GGTGGTAACC TTGGCAAGGC AGTGTTTCAA GGAAAGAAAT GACTGCTTAA
Macropus_rufogriseus      GGTGGTAACC TTGGCAAGGC AGTGTTTCAA GGAAAGAAAT GACTGCTTAA
Macropus_rufus            GGTGGTAACC TTGGCAAGGC AGTGTTTCAA GGAAAGAAAT GACTGCTTAA
Macropus_giganteus        GGTGGTAACC TTGGCAAGGC AGTGTTTCAA GGAAAGAAAT GACTGCTTAA
Onychogalea_unguifera     GGTGGTAACC TTGGCAAGGC AGTGTTTCAA GGAAAGAAAT GACTGCTTAA
Petrogale_lateralis       GGTGGTAACC TTGGCAAGGC AGTGTTTCAA GGAAAGAAAT GACTGCTTAA
Potorous_tridactylus      .......... .......... .......... .......... ..........
Setonix_brachyurus        GGTGGTAACC TTGGCAAGGC AGTGTTTCAA GGAAAGAAAT GACTGCTTAA
Thylogale_stigmatica      GGTGATAACC TTGGCAAGGC AGTGTTTCAA GGAAAGAAAT GACTGCTTAA
Didelphis_marsupialis     .......... .......... .......... .......... ..........
Lasiorhinus_latifrons     .......... .......... .......... .......... ..........
Petaurus_breviceps        .......... .......... .......... .......... ..........
Phascolarctos_cinereus    .......... .......... .......... .......... ..........
Pseudocheirus_peregrinus .......... .......... .......... .......... ..........
Spilocuscus_maculatus     .......... .......... .......... .......... ..........
Trichosurus_vulpecula     .......... .......... .......... .......... ..........
Monodelphis_domestica     .......... .......... .......... .......... ..........

                          2401                                               2450
Aepyprymnus_rufescens     .......... .......... .......... .......... ..........
Dendrolagus_goodfellowi   GCCATTAGGA TTAGTAGAAC CAGACCTGGA GCCTGTGAAA GGAGAAACTC
Dendrolagus_matschiei     GCCATTAGGA TTAGTAGAAC CAGACCTGGA GCCTGTGAAA GGAGAAACTC
Hypsiprymnodon_moschatus .......... .......... .......... .......... ..........
```

```
Lagostrophus_fasciatus   GCCATTAGGA GTAGTAGAAC CAGACCTGGA TCCTGTGAAA GGAGAAACTC
Macropus_eugenii         GCCATTAGGA GCAGTAGAAC CAGACCTGGA GCCTGTGAAA GGAGAAACTC
Macropus_parma           GCCATTAGGA GTAGTAGAAC CAGACCTGCA GCCTGTGAAA GGAGAAACTC
Macropus_robustus        GCCATTAGGA GTAGTAGAAC CAGACCTGGA GCCTGTGAAA GGAGAAACTC
Macropus_rufogriseus     GCCATTAGGA GTAGTAGAAC CAGACCTGGA GCCTGTGAAA GGAGAAACTC
Macropus_rufus           GCCATTAGGA GTAGTAGAAC CAGACCTGGA GCCTGTGAAA GGAGAAACTC
Macropus_giganteus       GCCATTAGGA GTAGTAGAAC CAGACCTGGA GCCTGTGAAA GGAGAAACTC
Onychogalea_unguifera    GTCATTAGGA GTAGTAGAAC CATACCTGGA GCCTGTGCAA GGAGAAACTT
Petrogale_lateralis      GCCATTAGGA TTAATAGAAC CAGACCTGGA GCCTGTGAAA GGAGAAACTC
Potorous_tridactylus     .......... .......... .......... .......... ..........
Setonix_brachyurus       GCCATTAGGA GTAGTAGAAC CAGAACTGGA GCCTGTGAAA GGAGAAACTC
Thylogale_stigmatica     GCCATTAGGA TTAGTAGAAC CAGACCTGGA GCCTGTGAAA GGAGAAACTC
Didelphis_marsupialis    .......... .......... .......... .......... ..........
Lasiorhinus_latifrons    .......... .......... .......... .......... ..........
Petaurus_breviceps       .......... .......... .......... .......... ..........
Phascolarctos_cinereus   .......... .......... .......... .......... ..........
Pseudocheirus_peregrinus .......... .......... .......... .......... ..........
Spilocuscus_maculatus    .......... .......... .......... .......... ..........
Trichosurus_vulpecula    .......... .......... .......... .......... ..........
Monodelphis_domestica    .......... .......... .......... .......... ..........

                         2451                                              2500
Aepyprymnus_rufescens    .......... .......... .......... .......... ..........
Dendrolagus_goodfellowi  CTGAGAAGCT GTGCATCCCT CAGCATCTCC AAATCCCTCC TCTTCCATCT
Dendrolagus_matschiei    CTGAGAAGCT GTGCATCCCT CAGCATCTCC AAATCCCTCC TCTTCCATCT
Hypsiprymnodon_moschatus .......... .......... .......... .......... ..........
Lagostrophus_fasciatus   CTGAGAAGCT GCACATCCCT CAGCAACTCC AAATCCTTCC TCCTCCATCT
Macropus_eugenii         CTGAGAAGCT GTGCATCCCT CAGCAACTCA AAATCCCTCC TCCTTCTTCT
Macropus_parma           CTGAGAAGCT GTGCATCCCT CAGCAACTCC AAATCCCTCC TCCTTCTTCT
Macropus_robustus        CTGAGAAGCT GTGCATCCCT CAGCAACTCC AAATCCCTCC TCCTTCTTCT
Macropus_rufogriseus     CTGAGAAGCT GTGCATCCCT CAGCAACTCC AAATCCCTCC TCTTTCTTTT
Macropus_rufus           CTGAGAAGCT GTGCATCCCT CAGCAACTCC AAATCCCTCC TCCTTCTTCT
Macropus_giganteus       CTGAGAAGCT GTGCATCCCT CAGCAACTCC AAATCCCTCC TCCTTCTTCT
Onychogalea_unguifera    CTGAGAAGCT GTGCATCCTT CAGCAACTCC AAATTCCTCC TTTT...TCT
Petrogale_lateralis      CTGAGAAGCT GCGCATCCCT CAGCATCTCC AAATCCCTCC TCTTCCATCT
Potorous_tridactylus     .......... .......... .......... .......... ..........
Setonix_brachyurus       CTGAGAAGCT GTGCATCCCT CAGCAACTCC AAATCCCTCC TCCTTCTTCT
Thylogale_stigmatica     CTGAAAAGCT GTGCATCCCT CAGCATCTCC AAATCCCTCC TCTTCCATCT
Didelphis_marsupialis    .......... .......... .......... .......... ..........
Lasiorhinus_latifrons    .......... .......... .......... .......... ..........
Petaurus_breviceps       .......... .......... .......... .......... ..........
Phascolarctos_cinereus   .......... .......... .......... .......... ..........
Pseudocheirus_peregrinus .......... .......... .......... .......... ..........
Spilocuscus_maculatus    .......... .......... .......... .......... ..........
Trichosurus_vulpecula    .......... .......... .......... .......... ..........
Monodelphis_domestica    .......... .......... .......... .......... ..........

                         2501                                              2550
Aepyprymnus_rufescens    .......... .......... .......... .......... ..........
Dendrolagus_goodfellowi  AAGCAACAAA AGATGAGAGG ACTCCCTTTC AACACAAACA GCGACAATGG
Dendrolagus_matschiei    AAGCAACAAA AGATGAGAGG ACTCCCTTTC AACACAAACA GCGACAATGG
Hypsiprymnodon_moschatus .......... .......... .......... .......... ..........
Lagostrophus_fasciatus   AAGGGACAAA AGACGAGAGG ACCCCCTTTT AACCGAAACA GCAACAATGG
Macropus_eugenii         AAAAGACAAA AGACAAGAGG ACTCCCTTTC AACCCAAACA GTGACAATGG
Macropus_parma           AAACGACAAA AGACAAGAGG ACTCCCTTTC AACCCAAACA GTGAAAATGG
Macropus_robustus        AAACGACAAA AGACAAGAGG ACTCCCTTTC AACCCAAACA ATGACAATGG
Macropus_rufogriseus     AAACGACAAA AGACAAGAGG ACTCCCTTTC AACCCAAACA GTGACAATGG
Macropus_rufus           AAACGACAAA AGACAAGAGG ACTCCCTTTC AACCCAAACA GTGACAATGG
Macropus_giganteus       GA........ .....AGAGG ACTCCCTTTC AACCCAAACA GTGACAATGG
Onychogalea_unguifera    AAACGACAAA AGACAAGAGG ACTCCCTTTA AACCCAAACA GTGACAATGG
Petrogale_lateralis      AAGCAACAAA AGATGAGAGG ACTCCCTTTC AACCCAAACA GAGACAATGG
Potorous_tridactylus     .......... .......... .......... .......... ..........
Setonix_brachyurus       AAACGACAAA AGACAAGAGG ACTCCTTTTC AACCCAAACA GTGACAATGG
```

```
Thylogale_stigmatica      AAGCAACAAA AGATGAGAGG ACTCCCTTTC AACCCAAACA GCGACAATGG
Didelphis_marsupialis     .......... .......... .......... .......... ..........
Lasiorhinus_latifrons     .......... .......... .......... .......... ..........
Petaurus_breviceps        .......... .......... .......... .......... ..........
Phascolarctos_cinereus    .......... .......... .......... .......... ..........
Pseudocheirus_peregrinus  .......... .......... .......... .......... ..........
Spilocuscus_maculatus     .......... .......... .......... .......... ..........
Trichosurus_vulpecula     .......... .......... .......... .......... ..........
Monodelphis_domestica     .......... .......... .......... .......... ..........

                          2551                                              2600
Aepyprymnus_rufescens     .......... .......... .......... .......... ..........
Dendrolagus_goodfellowi   AGCATACACC AGCAGTCAGC AATCAGTCCC CACTAATATG GGATCTGGTA
Dendrolagus_matschiei     AGCATACACC AGCAGTCAGC AATCAGTCCC CACTAATATG GGATCTGGTA
Hypsiprymnodon_moschatus  .......... .......... .......... .......... ..........
Lagostrophus_fasciatus    AGCATACACT AGCAGTCAGC GATCAGCCCC CACTAATTTG GGATCTGGTA
Macropus_eugenii          ACCATACAAC AGCATTCAGC AATCAGCCCC CACTAATTTG GGATCTGGTA
Macropus_parma            ACCATACAAG AGCATTCAGC AATCAGCCCC CACTAATTTG GGATCTGGTA
Macropus_robustus         ACCATACAAC AGCATTCCGC AATCAGCCCC CACTAATTTG GGATCTGGTA
Macropus_rufogriseus      ACCATACAAC AGCATTCAGC AATCAGCCCC CACTAATTTG GGATCTGGTA
Macropus_rufus            ACCATACAAC AGCATTCCGC AATCAGCCCC CACTAATTTG GGATCTGGTA
Macropus_giganteus        ACCATACAAC AGCATTCAGC AATCAGCCCC CACTAATTTG GGATCTGGTA
Onychogalea_unguifera     ACCATACAAC ACCATTCAGC AATCAGCCCT CACTAATGTG GGATCTGATA
Petrogale_lateralis       AGTATACACC AGCAGTCAGC AATCAGCCCC CACTAATATG GGATCTGGTA
Potorous_tridactylus      .......... .......... .......... .......... ..........
Setonix_brachyurus        ACCATACAAC AGCATTCAGC AATCAGCCCC ACTTAATTTG GGATCTGGTA
Thylogale_stigmatica      AGCATACACC AGCAGTCAGC AATCAGCCCC CACTAATATG GGATCTGGTA
Didelphis_marsupialis     .......... .......... .......... .......... ..........
Lasiorhinus_latifrons     .......... .......... .......... .......... ..........
Petaurus_breviceps        .......... .......... .......... .......... ..........
Phascolarctos_cinereus    .......... .......... .......... .......... ..........
Pseudocheirus_peregrinus  .......... .......... .......... .......... ..........
Spilocuscus_maculatus     .......... .......... .......... .......... ..........
Trichosurus_vulpecula     .......... .......... .......... .......... ..........
Monodelphis_domestica     .......... .......... .......... .......... ..........

                          2601                                              2650
Aepyprymnus_rufescens     .......... .......... .......... .......... ..........
Dendrolagus_goodfellowi   TCATGGCAGA AGGAGGTGGC ACATGAATRG GCAATAATCA ACAGGGTACT
Dendrolagus_matschiei     TCATGGCAGA AGGAGGTGGC ACATGAATGG GCAATAATCA ACAGGGTACT
Hypsiprymnodon_moschatus  .......... .......... ..ACCAGTGG GCGATAATGA ACAGGGTACC
Lagostrophus_fasciatus    TCATTGCAGA AGGAGGTGGC GCACCAATGG GCAATAACCA ACAGGGTGCT
Macropus_eugenii          TCATGGCAGA AGGAGGTGGA GTACCAATGG GCAATAATCA ACAGGGTGCT
Macropus_parma            TCATGGCAGA AGGAGGTGGA GTACCAATGG GCAATAATCA ACAGGGTGCT
Macropus_robustus         TCATGGCAGA AGGAGGTGGA GTACCAATGG GCAATAATCA ACAGGGTGCT
Macropus_rufogriseus      TCATGGCAGA AGGAGGTGGA GTACCAATGG GCAATAATCA ACAGGGTGCT
Macropus_rufus            TCATGGCAGA AGGAGGTGGA GTACCAATGG GCAATAATCA ACAGGGTGCT
Macropus_giganteus        TCATGGCAGA AGGAGGTGGA GTACCAATGG GCAATAATCA ACAGGGTGCT
Onychogalea_unguifera     TCATGGCAGA AGGAGGTGGA GTACCAATGG GCAATAATCA ACAGGATGCT
Petrogale_lateralis       TCATGGCAGA AGGAGGTGGC ACATGAATGG GCAATAATCA ACAGAGTACT
Potorous_tridactylus      .......... .......... .......... .......... ..........
Setonix_brachyurus        TCATGGCAGA AGGAGGTGGA GTACCGATGG GCAATAATCA ACAGGGTGCT
Thylogale_stigmatica      TCATGGCAGA AGGAGGTGGT GCATGAATAG GCAATAATCA ACAGGGTACT
Didelphis_marsupialis     .......... .......... .......... .......... ..........
Lasiorhinus_latifrons     .......... .......... .......... .......... ..........
Petaurus_breviceps        .......... .......... .......... .......... ..........
Phascolarctos_cinereus    .......... .......... .......... .......... ..........
Pseudocheirus_peregrinus  .......... .......... .......... .......... ..........
Spilocuscus_maculatus     .......... .......... .......... .......... ..........
Trichosurus_vulpecula     .......... .......... .......... .......... ..........
Monodelphis_domestica     .......... .......... .......... .......... ..........

                          2651                                              2700
```

```
Aepyprymnus_rufescens     .......... .......... .......... .......... ..........
Dendrolagus_goodfellowi   GATGGAGTAG GTAATTCCTC AGGAAATTAA CATTGTGATT CCCAATGGAT
Dendrolagus_matschiei     GATGGAGTAG GTAATTCCTC AGGAAATTAA CATTGTGATT CCCAATGGAT
Hypsiprymnodon_moschatus  GATGGAGTGG GTAACTCGTC GGGAAATTGG CATTGCCATT CCCAATGGA.
Lagostrophus_fasciatus    GATGGAGTAG GTAATTCCTC AGGAAATTAG CATTGTGATT CCCAATGGAT
Macropus_eugenii          GATAGAGTAG GTAATTCTTT GGGAAATTAG CATTGTGGTT CTCAATGGAT
Macropus_parma            GATAGAGTAG GTAATTCTTT GGGAAATTAG CATTGTGATT CTCAATGGAT
Macropus_robustus         GATAGAG... .......... .GGAAATTAG CATTGTGATT CTCAATGGAT
Macropus_rufogriseus      GATAGAGTAG GAAATTCTTC GGGAAATTAG CATTGTGATT CTCAATAGAT
Macropus_rufus            GATAGAG... .......... .GGAAATTAG CATTGTGATT CTCAATGGAT
Macropus_giganteus        GATAGAGTAG GTAATTCTTC GGGAAATTAG CATTGTGATT CTCAATGGAT
Onychogalea_unguifera     GATAGAGTAG GTAATTCTTC GGGAAATTAG CATTGTGATT CTCAATGGAT
Petrogale_lateralis       GATGGAGTAG GTAATTCCTC AGGAAATTAA CATTGTGATT CCCAATGGAT
Potorous_tridactylus      .......... .......... .......... .......... ..........
Setonix_brachyurus        GATAGAGTAG GTAATTCTTC TGGAAATTAG CATTGTGATT CTCAATGGAT
Thylogale_stigmatica      GATGGAGTAG GTAATTCCTC AGGAAATTAA CATTGTGATT CCCAATGGAT
Didelphis_marsupialis     .......... .......... .......... .......... ..........
Lasiorhinus_latifrons     .......... .......... .......... .......... ..........
Petaurus_breviceps        .......... .......... .......... .......... ..........
Phascolarctos_cinereus    .......... .......... .......... .......... ..........
Pseudocheirus_peregrinus  .......... .......... .......... .......... ..........
Spilocuscus_maculatus     .......... .......... .......... .......... ..........
Trichosurus_vulpecula     .......... .......... .......... .......... ..........
Monodelphis_domestica     .......... .......... .......... .......... ..........

                          2701                                              2750
Aepyprymnus_rufescens     .......... .....CACCC TAAAAACTC. ..TCTGGGTC TTACCCACCT
Dendrolagus_goodfellowi   GGGCCACAGA GTCGTCACCT GAAAAACTC. ..TCTGGGTC TTGCCCACCT
Dendrolagus_matschiei     GGGCCACAGA GTCGTCACCT GAAAAACTC. ..TCTGGGTC TTGCCCACCT
Hypsiprymnodon_moschatus  .......... .CCGTCACCC GAATAATTCA CACCTGGGTC TTGCTCACCT
Lagostrophus_fasciatus    GGGCAACATA GTCGCCACCT GAAAAACTC. ..TCTGGGTC TTGCCCACTT
Macropus_eugenii          GGGCCACAGA GTCACCACCC GAAAAGCTC. ..TCTGGGTC TCGCCTACCT
Macropus_parma            GGGCCACAGA GTCACCACCC GAAAAGCTC. ..TCTGGGTC TTGCCTACCT
Macropus_robustus         GGGCCACAGA GTCACCACCC GAAAAGCTC. ..TCTGGGTC TTGCCTACCT
Macropus_rufogriseus      GGGCCACAGA ATCACCACCC GAAAAGCTC. ..TCTGGATC TAGCCTACCT
Macropus_rufus            GGGCCACAGA GTCACCACCC GAAAAGCTC. ..TCTGGGTC TTGCCTACCT
Macropus_giganteus        GGGCCACAGA GTCACCACCC GAAAAGCTC. ..TCTGGGTC TAGCCTACCT
Onychogalea_unguifera     GGGCCACAGA GTCACCMCCC GAAAAGCTC. ..TCTGGGTC TTGCCTACCT
Petrogale_lateralis       GGGCCACAGA GTCATCACCT GAAAAACTC. ..TCTGGGTC TTGCCCACCT
Potorous_tridactylus      .......... .....CACCC TAAAAACTC. ..TCTGGGTC TTACCTACCT
Setonix_brachyurus        GGGCCACAGA GTCACCACCC GAAAAGCTC. ..TCTGGGTC TAGCCTACCT
Thylogale_stigmatica      GGGCCACAGA GTCACCACCT GAAAAACTC. ..TCTGGGTC TTGCCCACCT
Didelphis_marsupialis     .......... .......... .......... .......... ..........
Lasiorhinus_latifrons     .......... .......... .......... .......... ..........
Petaurus_breviceps        .......... .......... .......... .......... ..........
Phascolarctos_cinereus    .......... .......... .......... .......... ..........
Pseudocheirus_peregrinus  .......... .......... .......... .......... ..........
Spilocuscus_maculatus     .......... .......... .......... .......... ..........
Trichosurus_vulpecula     .......... .......... .......... .......... ..........
Monodelphis_domestica     .......... .......... .......... .......... ..........

                          2751                                              2800
Aepyprymnus_rufescens     GCAACAACCA CCTCTACAAG C.AGTTGAAA ACAGTGTCAC CACAGGCAGT
Dendrolagus_goodfellowi   GCAACAATTG CCTCTACAAG C.AGTTCAAA GCAGTGTTAC CACAGGCAGT
Dendrolagus_matschiei     GCAACAATTG CCTCTACAAG C.AGTTCAAA GCAGTGTTAC CACAGGCAGT
Hypsiprymnodon_moschatus  ACAACAACCA CCTCTGCAAA CGAGTTCAAA ACAGTGTCAC CACAGGCAGT
Lagostrophus_fasciatus    GCAACAACCA CCTCTACAAG C.AGTTCAAA ACAATGGCAC CACAGGCAGT
Macropus_eugenii          GCAACAACCA CCTCTACAAG C.AGTTCAAA ATAGTGCCAC CACAGGCAGT
Macropus_parma            GCAACAACCA CCTCTACAAA C.AGTTCAAA ATAGTGTCAC CACAGGCAGT
Macropus_robustus         GCAACAACCA CCTCTACAAG C.AGTTCAAA ATAGTGTCAC CACAGGCAGT
Macropus_rufogriseus      GCAACAACCA CCTCTACAAG C.AGTTCAAA ATAGTGTCAC CACAGGCAGT
Macropus_rufus            GCAACAACCA CCTCTACAAG C.AGTTCAAA ATAGTGTCAC CACAGGCAGT
Macropus_giganteus        GCAACAACCA CCTCTACAAG C.AGTTCAAA ATAGTGTCAC CACAGGCAGT
```

```
Onychogalea_unguifera    GCAACAACCA CCTCTACAAG C.AGTTCAAA ATAGTGTCAC CACAGGCAGT
Petrogale_lateralis      GCAACAATTG CCTCTACAAG C.AGTTCAAA GCAGTGTCAC CACAGGCAGT
Potorous_tridactylus     GCAACAATCA CCTGTACAAA C.AGTTGAAA ACAGTGTCAC CACAGGCAGT
Setonix_brachyurus       GCAACAACCA CCTCTACAAG C.AGTTCAAA ATAGTGTCAC CACAGGCAGT
Thylogale_stigmatica     GCAACAATTG CCTCTACAAG C.AGTTCAAA GCAGTGTCAC CACAGGCAGT
Didelphis_marsupialis    .......... .......... .......... .......... ..........
Lasiorhinus_latifrons    .......... .......... .......... .......... ..........
Petaurus_breviceps       .......... .......... .......... .......... ..........
Phascolarctos_cinereus   .......... .......... .......... .......... ..........
Pseudocheirus_peregrinus .......... .......... .......... .......... ..........
Spilocuscus_maculatus    .......... .......... .......... .......... ..........
Trichosurus_vulpecula    .......... .......... .......... .......... ..........
Monodelphis_domestica    .......... .......... .......... .......... ..........

                         2801                                              2850
Aepyprymnus_rufescens    GCCAACAAAT ...ACTTTGG CTTCAGCACC TCCTCGGGGC ATGTTGACTT
Dendrolagus_goodfellowi  GCCAACAACT ...ACTTTGG CTTCAGMACC CCCTGGGGGA ATTTTGACTT
Dendrolagus_matschiei    GCCAACAACT ...ACTTTGG CTTCAGAACC CCCTGGGGGA ATTTTGACTT
Hypsiprymnodon_moschatus GCCAACAACA ...ACTTTGG CTTCAGCATC CCCTGAGGGT ATTTTGACTT
Lagostrophus_fasciatus   GCCAACAACT ...ACTTTGG CTTCAGCACC CCCTGGGGGT ATTTTGACTT
Macropus_eugenii         GCCAACAACT ...ACTTTGG CTTCAGCTCC CCCTGGGGGA ATTTTGACTT
Macropus_parma           GCCAACAACT ...ACTTTGG CTTCAGCACC CCCTGGGGGA ATTTTGACTT
Macropus_robustus        GCCAACAACT ...ACTTTGG CTTCAGCACC CCCTGGGGGA ATTTTGACTT
Macropus_rufogriseus     GCCAACAACT TCAGCTTTGG CTTCAGTACC CTCTGGGGGA ATTTTGACTT
Macropus_rufus           GCCAACAACT ...ACTTTGG CTTCAGCACT CCCTGGGGGA ATTTTGACTT
Macropus_giganteus       GCCAACAACT ...ACTTTGG CTTCAGCTCC ACCTGGGGGA ATTTTGACTT
Onychogalea_unguifera    GCCACCAACT ...ACTTTGG CTTCAGCACC CTGTGGGGGA ATTTTGACTT
Petrogale_lateralis      GCCAACAACT ...ACTTTGG CTTCAGCACC CCCTGGGGGA ATTTTGACTT
Potorous_tridactylus     GCCAACAACT ...ACTTTGG CTTCAGCACC TCCTGGGGGT ATTTTGACTT
Setonix_brachyurus       GCCAACAACT ...ACTTTGG CTTCAGCACC CCCTGGGGGA ATTTTGACTT
Thylogale_stigmatica     GCCAACAACT ...ACTTTGG CTTCAGCACC CCCTGGGGGA ATTTTGACTT
Didelphis_marsupialis    .......... .......... .......... .......... ..........
Lasiorhinus_latifrons    .......... .......... .......... .......... ..........
Petaurus_breviceps       .......... .......... .......... .......... ..........
Phascolarctos_cinereus   .......... .......... .......... .......... ..........
Pseudocheirus_peregrinus .......... .......... .......... .......... ..........
Spilocuscus_maculatus    .......... .......... .......... .......... ..........
Trichosurus_vulpecula    .......... .......... .......... .......... ..........
Monodelphis_domestica    .......... .......... .......... .......... ..........

                         2851                                              2900
Aepyprymnus_rufescens    CAACAGATTC CACTGCCACT TCAGCCCCTG AGACTGGCAA AGACTTATCA
Dendrolagus_goodfellowi  CAACAGATTC CACTGCCACT TCAGCCCCTG AGACTGGCAA AGACTTATCA
Dendrolagus_matschiei    CAACAGATTC CACTGCCACT TCAGCCCCTG AGACTGGCAA AGACTTATCA
Hypsiprymnodon_moschatus CAGCAGATTC CACTGCCACT TTGGCCCCCG AGACTGGCAA AGACTCATCA
Lagostrophus_fasciatus   TAACAGATTC CACTGCCCCT TCAGCCCCTG AGATTGGCAA AGACTTATCA
Macropus_eugenii         CAACAGATTC CACTGCCACT TCAGCCCCTG AGACTGGCAA AGACTTATCA
Macropus_parma           CAACAGATTC CACTGCCACT TCAGCCCCCT GGACTGGCAA AGACTTATCA
Macropus_robustus        CAACAGATTC CACTGCCACT TCAGCCCCCT AGACTGGCAA AGACTCATCA
Macropus_rufogriseus     CAACAGATTC CACTGCCACT TCAGCCCCCG AGACTGGCAA AGACTTATCA
Macropus_rufus           CAACAGATTC CACTGCCACT TCAGCCCCCT AGACTGGCAA AGACTTATCA
Macropus_giganteus       CAACAGATTC CACTGCCACT TCAGCCCCCT AGACTAGCAA AGACTTATCA
Onychogalea_unguifera    CAACAAATTC CACTGCCACT TCAGCCCCCG AGACTGGCAA AGACTTATCA
Petrogale_lateralis      CAACAGATTC CACTGCCACT TCAGCCCCCG AGACTGGCAA AGACTTATCA
Potorous_tridactylus     TAACAGATTC CACTGCCACT TCAGCCCCTG AGACT.GCAA AGACTTATCA
Setonix_brachyurus       CAACAGATTC CACTGCCACT TCAGCCCCTG AGACTGGCAA AGACTTAGCA
Thylogale_stigmatica     CAACAGATTC CACTGCCACT TCAGTCCCCG AGACTGGCAA AGACTTATCA
Didelphis_marsupialis    .......... .......... .......... .......... ..........
Lasiorhinus_latifrons    .......... .......... .......... .......... ..........
Petaurus_breviceps       .......... .......... .......... .......... ..........
Phascolarctos_cinereus   .......... .......... .......... .......... ..........
Pseudocheirus_peregrinus .......... .......... .......... .......... ..........
Spilocuscus_maculatus    .......... .......... .......... .......... ..........
```

```
Trichosurus_vulpecula    .......... .......... .......... .......... ..........
Monodelphis_domestica    .......... .......... .......... .......... ..........

                         2901                                              2950
Aepyprymnus_rufescens    ATAACAACTG GAGCCTGCGA CCTAAAAACC TCCACTTCAA ACT..TCAAC
Dendrolagus_goodfellowi  ATAATAACTR GGGACTGTGA CCTAAAAACC TRCACTTCAA ACTCTTCAAC
Dendrolagus_matschiei    ATAATAACTT GGGACTGTGA CCTAAAAACC TGCACTTCAA ACTCCTCAAC
Hypsiprymnodon_moschatus A.AACAACTA GGAACTGTGA CCTAAAAACC TGCACTTCCA ACTCTTCAAC
Lagostrophus_fasciatus   ATAACAACTG GGAACTGCAA CCTAAAAACC TGCGCTTCAA ACTCTTCAAC
Macropus_eugenii         ATAACAACTG GGGACTGCGA CCTAAAAACC TGCACTTCAA ACTCTTCAAC
Macropus_parma           ATAACAACTG GGGACTGCGA CCTAAAAACC TGCACTTCAA ACTCTTCAAC
Macropus_robustus        ATAACAACTG GGGACTGCGA CCTAAAAACC TGCACTTCAA ACTCTTCAAC
Macropus_rufogriseus     ATAACAACTG GGGACTGTGA CCTAAAAACC TGCACTTCAA ACTCTTTAAC
Macropus_rufus           ATAACAACTG GGGACTGCGA CCTAAAAACC TGCACTTCAA ACTCTTCAAC
Macropus_giganteus       ATAACAACTG GGGACTGAGA CCTAAAAACC TGCACTTCAA ACTCTTCAAC
Onychogalea_unguifera    ATAACAAYTG GGGACTGCGA CCTAAAAACC TGCACTTCAA ACTCTTCAGC
Petrogale_lateralis      ATAATAACTG GGGACTGTGA TCTAAAAACC TGCACTTCAA ATTCTTCAAC
Potorous_tridactylus     ATAACAACTG GGGACTGCGA CCTAAAAACC TGCACTTCAG ACTCTTCAAC
Setonix_brachyurus       ATAACAACTG GGTACTGTGA CCTAAAAACC TGCACTTCAA ACTCTTCAAC
Thylogale_stigmatica     ATAATAACTG GGGACTGTGA TCTAAAAACC TGCACTTCAA ACTCTTCAAC
Didelphis_marsupialis    .......... .......... .......... .......... ..........
Lasiorhinus_latifrons    .......... .......... .......... .......... ..........
Petaurus_breviceps       .......... .......... .......... .......... ..........
Phascolarctos_cinereus   .......... .......... .......... .......... ..........
Pseudocheirus_peregrinus .......... .......... .......... .......... ..........
Spilocuscus_maculatus    .......... .......... .......... .......... ..........
Trichosurus_vulpecula    .......... .......... .......... .......... ..........
Monodelphis_domestica    .......... .......... .......... .......... ..........

                         2951                                              3000
Aepyprymnus_rufescens    ATCCAAGTCA AGGAGGTCAC AAGGAGGAAT GTTGAG.... ..........
Dendrolagus_goodfellowi  ATCCAAGTCA AGGAGGTCAC AAGGAGGAAT GTTGAGACCA CAATTGCTAA
Dendrolagus_matschiei    ATCCAAGTCA AGGAGGTCAC AAGGAGGAAT GTTGAGACCA CAATTGCTAA
Hypsiprymnodon_moschatus ATTCAAGTCA AGGAGGTCAC GATGACGATT GGCAAGACGA AGATTGCTAA
Lagostrophus_fasciatus   ATCCAAGTCA AGGAGGTCAC AAGGAGGAAT GTTGAGACCA CATTTGCTAA
Macropus_eugenii         ATCCAAGTCA AGGAAGTCAC AAGGAGGAAT GTTGAAACCA CAATTGCTAA
Macropus_parma           ATCCAAGTCA AGGAGGTCAC AAGGAGGAAT GTTGAAACTA CAATTGCTAA
Macropus_robustus        ATCCAAGTCA AGGAGGTCAC AAGGAGGAAT GTTGAAACCA CAATTGCTAA
Macropus_rufogriseus     ATTCAAGTCA AGGAGGTCAC AAGGAGGAAT GTTGAAACCA CAATTGCTAA
Macropus_rufus           ATCCAAGTCA AGGAGGTCAC AAGGAGGAAT GTTGAAACCA CAATTGCTAA
Macropus_giganteus       ATCCAAGTCA AGGAGGTCAC AAGGAGGAAT GTTGAAACCA CAATTGCTAA
Onychogalea_unguifera    ATCCAAGTCA AGGAGGTCAC AAGGAGGAAT GTTGAAACCA CAATTGCTAA
Petrogale_lateralis      ATCCAAGTCA AGGAGGTCAC AAAGAGGAAT GTTGAGACCA CAATTGCTAA
Potorous_tridactylus     ATCCAAGTCA AGGAGATCAC AAGGAGGAAT GTAGAGACCA CAATTGCTAA
Setonix_brachyurus       ATCCAAGTCA AGGAGGTCAC AAGGAAGAAT GTTGAAACCA CAATTGCTAA
Thylogale_stigmatica     ATCCAAGTCA AGGAGGTCAC AAGGAGGAAT GTTGAGACCA CAATTGCTAA
Didelphis_marsupialis    .......... .......... .......... .......... ..........
Lasiorhinus_latifrons    .......... .......... .......... .......... ..........
Petaurus_breviceps       .......... .......... .......... .......... ..........
Phascolarctos_cinereus   .......... .......... .......... .......... ..........
Pseudocheirus_peregrinus .......... .......... .......... .......... ..........
Spilocuscus_maculatus    .......... .......... .......... .......... ..........
Trichosurus_vulpecula    .......... .......... .......... .......... ..........
Monodelphis_domestica    .......... .......... .......... .......... ..........

                         3001                                              3050
Aepyprymnus_rufescens    .......ACC AGCATGATTC AAGTCTTTGT GGACTC...A ATGTCAACTC
Dendrolagus_goodfellowi  TAACCTTACC AACATGATTC AAGTCTTTGY GGACTCAAGA GTGTTAACTC
Dendrolagus_matschiei    TAACCTTACC AACATGATTC AAGTCTTTGC GGACTCAAGA GTGTTAACTC
Hypsiprymnodon_moschatus TAACTTTACC AGCACGAATC AATTCTCAGC GGACTC.AGA CTATCAACTC
Lagostrophus_fasciatus   TAACCTTACC AGCATGATTC AAGTCTTTGT GGACTC...A GTGTCAACTC
Macropus_eugenii         TAACCTTACC AGCATGATTC AAGTCTTTGC GGACTC.AGA GTGTCAACTC
Macropus_parma           TAACCTTACC AGCATGATTC AAGTCTTTGC GGACTC.AGA GTGTCAACTC
```

```
Macropus_robustus         TAACCTTACT AGCATGATTC AAGTCTTTGC GGACTC.AGA GTGTCAACTC
Macropus_rufogriseus      TAACCTTACC AGCATGATTC AAGTCTTTGC GGACTC.AGA GTGTCAACTC
Macropus_rufus            TAACCTTACT AGCATGATTC AAGTCTTTGC GGACTC.AGA GTGTCAACTC
Macropus_giganteus        TAACCTTACC AGCATGATTC AAGTCTTTGC GGACTC.AGA GTGTCAACTC
Onychogalea_unguifera     TAACCTTACC AGCATGACTC AAGTCTTTGC GGACTC.AGA GTGTCAACTC
Petrogale_lateralis       TAACCTTACC AACATGATTC AAGTCTTTGC GGACTC.AGA GTGTTAACTC
Potorous_tridactylus      TAACCTTACC AGCATGATTC AAGTCTTTGC GGACTC...A GTGTCAACTC
Setonix_brachyurus        TAACCTTACC AGCATGATTC AAGTCTTTGC GGACTC.GGA GTGTCAACTC
Thylogale_stigmatica      TAACCTTACC GACATGATTC AAGTCTTTGC AGACTCAAGA ATGTTAACTC
Didelphis_marsupialis     .......... .......... .......... .......... ..........
Lasiorhinus_latifrons     .......... .......... .......... .......... ..........
Petaurus_breviceps        .......... .......... .......... .......... ..........
Phascolarctos_cinereus    .......... .......... .......... .......... ..........
Pseudocheirus_peregrinus  .......... .......... .......... .......... ..........
Spilocuscus_maculatus     .......... .......... .......... .......... ..........
Trichosurus_vulpecula     .......... .......... .......... .......... ..........
Monodelphis_domestica     .......... .......... .......... .......... ..........

                          3051                                             3100
Aepyprymnus_rufescens     ACTTATATGA TCAGGAGTGC TCAAGAGGGG TGTCTA.CTC CCCTTCCCTC
Dendrolagus_goodfellowi   CCATATGTGA CCAGGAGTGC TCAAGAGAGG TGTCTA.CTC CCCTTCCCTC
Dendrolagus_matschiei     CCATATGTGA CCAGGAGTGC TCAAGAGAGG TGTCTA.CTC CCCTTCCCTC
Hypsiprymnodon_moschatus  CCATACGTGA TCAGAAGTGC GCATGAG.GG TGTCTACCTC CCCTTCCCTC
Lagostrophus_fasciatus    CCATACATGA TCAGGTATGC TCAAGAGGGG TGTCTA.CTT CCCTTCCCTC
Macropus_eugenii          CCATATGTGA TCAGGAGTGC TCAAGAGAGG TGCCTA.CTC CCCTTCCCTC
Macropus_parma            CCATACGTGA TCAGGAGTGC TCAAGAGAAG TGCCTA.CTC CCTTTCCCTC
Macropus_robustus         CCATACGTGA TCAGGAGTGC TCAAGAGAGG TGCCTA.CTC CCCTTCCCTC
Macropus_rufogriseus      CCATACGTGA TGAGGAGTGC TCAAGAGAGG TGCCTA.CTC CCCTTCCCTC
Macropus_rufus            CCATACGTGA TCAGGAGTGC TCAAGAGAGG TGCCTA.CTC CCCTTCCCTC
Macropus_giganteus        CCATATGTGA TCAGGAGTGT TCAAGAGAGG TGCCTA.CTC CCCTTCCCTC
Onychogalea_unguifera     CCATACGTGA TCAGGAGTGC TCAAGAGAGG TGTCTA.CTC CCCTTCCCTC
Petrogale_lateralis       CCATATGTGA CCAGGAGTGC TCAAGAGAGG TGTCTA.CTC CCCTTCCCTC
Potorous_tridactylus      CCATACATGA CCAGGAGTGC CCAAGAGGGG TGTCTG.CTT CCCTTCCCTC
Setonix_brachyurus        CCATACATGA TCAGGAGTGC TCAAGAGCGG TGCCTA.CTC TTCTTCCCTC
Thylogale_stigmatica      CCATATGTGA CCAGGAGTGC TCAAGAGAGG TATCTA.CTC CCCTTCCCTC
Didelphis_marsupialis     .......... .......... .......... .......... ..........
Lasiorhinus_latifrons     .......... .......... .......... .......... ..........
Petaurus_breviceps        .......... .......... .......... .......... ..........
Phascolarctos_cinereus    .......... .......... .......... .......... ..........
Pseudocheirus_peregrinus  .......... .......... .......... .......... ..........
Spilocuscus_maculatus     .......... .......... .......... .......... ..........
Trichosurus_vulpecula     .......... .......... .......... .......... ..........
Monodelphis_domestica     .......... .......... .......... .......... ..........

                          3101                                             3150
Aepyprymnus_rufescens     CTGATGTGTT TATGTTGCCT CAGTATGGGT ATTGTACTTT GGACAATGAT
Dendrolagus_goodfellowi   CTGATGTGTT TATGTTGCCT CAATATGGGT ATTGTACTTT GGACAATGAT
Dendrolagus_matschiei     CTGATGTGTT TATGTTGCCT CAATATGGGT ATTGTACTTT GGACAATGAT
Hypsiprymnodon_moschatus  CTGATGCGTT TATGTTGCCT CAGTATGGGT ATTGTACTTT GGACAATGAT
Lagostrophus_fasciatus    CTG.TGTGTT TATGTTGCCT CAGTATGGGT ATTGTACCTT GGACAATGAT
Macropus_eugenii          CTGATGTGTT TATGTTGCCT CAGTATGGAT ATTGTACTTT GGACAATGAT
Macropus_parma            CTGATGTGTT TATATTGCCT CAGTATGGAT ATTGTACTTT GGACAGTGAT
Macropus_robustus         CTGATGTGTT TATGTTGCCT CAGTATGGAT ATTGTACTTT GGACAGTGAT
Macropus_rufogriseus      CTGATGTGTT TGTGTTGCCT CAGTATGGAT ATTGTACTTT GGACAATGAT
Macropus_rufus            CTGATGTGTT TATGTTGCCT CAGTATGGAT ATTGTACTTT GGACAGTGAT
Macropus_giganteus        CTGATGTGTT TATGTTGCCT CAGTATGGAT ATTGTACTTT GGACAATGAT
Onychogalea_unguifera     CTGATGTGTT TATGTTGCCT CAGTATGGAT GTTGTACTTT GGACAATGAT
Petrogale_lateralis       CTGATGTGTT TATGTTGCCT CAATATGGGT ATTGTACTTT GGACAATGAT
Potorous_tridactylus      CTGATGTGTT TATGTTGCCT CAGTATGGGT ATTGTACTTT GGACAATGAT
Setonix_brachyurus        CTGATGTGTT TATGTTGCCT CAGTATGGAT ATTGTACTTT GGACAATGAT
Thylogale_stigmatica      CTGATGTGTT TATGTTGCCT CAATATGGGT ATTGTACTTT GGACAATGAT
Didelphis_marsupialis     .......... .......... .......... .......... ..........
Lasiorhinus_latifrons     .......... .......... .......... .......... ..........
```

```
Petaurus_breviceps        .......... .......... .......... .......... ..........
Phascolarctos_cinereus    .......... .......... .......... .......... ..........
Pseudocheirus_peregrinus  .......... .......... .......... .......... ..........
Spilocuscus_maculatus     .......... .......... .......... .......... ..........
Trichosurus_vulpecula     .......... .......... .......... .......... ..........
Monodelphis_domestica     .......... .......... .......... .......... ..........

                          3151                                              3200
Aepyprymnus_rufescens     GGGGAAAGTT TAGACAGGAG TGCATTCTAC TGTCTAGAAT ATTTTCC...
Dendrolagus_goodfellowi   GGGAAAAGTT TAGAGAGGAG TACATTCTAC TGTCTAGTAT ATTTTCC...
Dendrolagus_matschiei     GGGAAAAGTT TAGAGAGGAG TACATTCTAC TGTCTAGTAT ATTTTCC...
Hypsiprymnodon_moschatus  GGGAAAAGCT CAGAGAAGAG TGAGTTCTAC TGTCTAGGGT ATTTTCC...
Lagostrophus_fasciatus    GGGAAAAGTT TAGAGAGGAG TGCATTCTAC TGTCTAGAAT ATTTTCC...
Macropus_eugenii          GGGAAAAGTT TAGAGAGGAG CGCATTCTAC TTTCTAGAAC ATTTTCC...
Macropus_parma            GGGGAAAGTT TAGAGAGGAG TGCATTCTAC TTTCTAGAAT ATTTTCC...
Macropus_robustus         GGGAAAAGTT TAGAGAGGAG CACATTCTAC TTTCTAGAAT ATTTTCC...
Macropus_rufogriseus      GGGAAAAGTT TAGAGAGGAG CGCATTCTAC TTTCTAGAAT ATTTTCCTCA
Macropus_rufus            GGGAAAAGTT TAGAGAAGAG CGCATTCTAC TTTCTAGAAT ATTTTCC...
Macropus_giganteus        GGGAAAAGTT TAGAGAGGAG CGCATTCTAC TTTCTAGAAT ATTTTTC...
Onychogalea_unguifera     GGGAAAAGTT TAGAGAGGAG CGCATTCTAC TTTCTAGAAT ATTTTCC...
Petrogale_lateralis       GGGAAAAGTT TAGAGAGGAG TGCATTCTAC TGTCYAGTAT ATTTTCC...
Potorous_tridactylus      GGGAAAAGTT TAAAGAGGAG TGCATTCTAC TGTCTAGAAT ATTTTCC...
Setonix_brachyurus        GGGAAAAGTT CAGAGAGGAG TGCATTCTAC TTTCTAGAAT ATTTTCC...
Thylogale_stigmatica      GGGAAAAGTT TAGAGAGGAG TGCATTCTAC TGTCTAGTAT ATTTTCC...
Didelphis_marsupialis     .......... .......... .......... .......... ..........
Lasiorhinus_latifrons     .......... .......... .......... .......... ..........
Petaurus_breviceps        .......... .......... .......... .......... ..........
Phascolarctos_cinereus    .......... .......... .......... .......... ..........
Pseudocheirus_peregrinus  .......... .......... .......... .......... ..........
Spilocuscus_maculatus     .......... .......... .......... .......... ..........
Trichosurus_vulpecula     .......... .......... .......... .......... ..........
Monodelphis_domestica     .......... .......... .......... .......... ..........

                          3201                                              3250
Aepyprymnus_rufescens     TAGCCAAATG TTGAGAATGG GTAACAACTT TGAATTTTCC TATGCTTTTG
Dendrolagus_goodfellowi   TAGCTAAATA TTGAGAATGG GTAACAACTT TGAATTTTCC TATGCTTTTA
Dendrolagus_matschiei     TAGCTAAATA TTGAGAATGG GTAACAACTT TGAATTTTCC TATGCTTTTA
Hypsiprymnodon_moschatus  TAGTCAAATG TTGAGAACGG GTAACAACTT TGAATTTTCC TATGCTTTTG
Lagostrophus_fasciatus    TAGCCAAATA TTGAGAATGG GCAACAGCTT TGAATTTTCC TATGCTTTTG
Macropus_eugenii          TAACCAAATA TTGAGAATGG GTAACAACTT TGCATTTTCC TATGCTTTTG
Macropus_parma            TAACCAAATA TTGAGAACGG GTAACAACTT TGCATTTTCC TATGCTTTTG
Macropus_robustus         TAACCAAATA TTGAGAACGG GTAACAACTT TGCATTTTCC TATGCTTTTG
Macropus_rufogriseus      TCACCAAATA TTGAGAATGG GTAACAACTT TGCATTTTCC TATGCTTTTG
Macropus_rufus            TAACCAAATA TTGAGAATGG GTAACAACTT TGCATTTTCC TATGCTTTTG
Macropus_giganteus        TAACCAAATA TTGAGAACGG GTAACAACTT TGCATTTTCC TATGCTTTTG
Onychogalea_unguifera     TAACCAAATA TTGAGAATGG GTAACAACTT TGCATTTTCC TATGCTTTTG
Petrogale_lateralis       TAGCCAAATA TTGAGAACRG GTAACAACTT TGAATTTTCC TATGCTTTTA
Potorous_tridactylus      TAGCCAAATG TTGAGAACAG GTAACAACTT TGAATTTTCC TATGCTTTTG
Setonix_brachyurus        TAACCAAATA TTGAGAACGG GTAACAACTT TGCATTTTCC TATGCTTTTG
Thylogale_stigmatica      TAGCCAAATA TTGAGAACGG GTAACAACTT TGAATTTTCC TATGCTTTTA
Didelphis_marsupialis     .......... .......... .......... .......... ..........
Lasiorhinus_latifrons     .......... .......... .......... .......... ..........
Petaurus_breviceps        .......... .......... .......... .......... ..........
Phascolarctos_cinereus    .......... .......... .......... .......... ..........
Pseudocheirus_peregrinus  .......... .......... .......... .......... ..........
Spilocuscus_maculatus     .......... .......... .......... .......... ..........
Trichosurus_vulpecula     .......... .......... .......... .......... ..........
Monodelphis_domestica     .......... .......... .......... .......... ..........

                          3251                                              3300
Aepyprymnus_rufescens     AATCTGTCCC CTTTCATAGC ATGCGGTTGC ATAATCAGAG CTTGGATAGA
Dendrolagus_goodfellowi   AATCTGTCCC CTTTCATAGC ATGTGGATGC ATAATCAGAG CTTGGATAGA
Dendrolagus_matschiei     AATCTGTCCC CTTTCATAGC ATGTGGATGC ATAATCAGAG CTTGGATAGA
```

```
Hypsiprymnodon_moschatus AATCTGTCCC CTTTCATAGA ATGTGAATGC ATAATCAGAG CTTGG.TAGA
Lagostrophus_fasciatus   AATCTGTCCA CTTTCATAGC ATGTGGATGC ATAATCAGAG CATGGATAGA
Macropus_eugenii         AATCTGTCCC CTTTCATAGC ATGTGGATAC ATAATCAGAG CTTGGATAGA
Macropus_parma           AATCTGTCCC CTTTTATAGC ATGTGGATAC ATAATCAGAC CTTGGATAGA
Macropus_robustus        AATCTGTCCT CTTTTATAGC ATGTGGATAC ATAATCAGAC CTTGGATAGA
Macropus_rufogriseus     AATCTGTCCC CTTTCATAGC ATGTGGATAC ATAATCAGAG CTTGGATAGA
Macropus_rufus           AATCTGTCCT CTTTTATAGC ATGTGGATAC ATAATCAGAC CTCGGATGGA
Macropus_giganteus       AATCTGTCCC CTCTCATAGC ATGTGGATAC ATAATCAGAG CTTGGATAGA
Onychogalea_unguifera    AATCTGTCCC CTTTCATAGC ATGTGGATAC ATAATCAGAG CTTGGATAGA
Petrogale_lateralis      AATCTGTCCC CTTTCATAGC ATGTGGATGC ATAATCAGAG CTTGGATAGA
Potorous_tridactylus     AATCTGTCCC CTTTCATAGC ATGTGAATGC ATAATCAGAG CTTGGATAGA
Setonix_brachyurus       AATCTGTCCC CTTTCATAGC ATATGGATAC ATAATCAGAG CTTGCATAGA
Thylogale_stigmatica     AATCTGTCCC CTTTCATAGA ATGTGGATGC ATAATCAGAG CTTGGATAGA
Didelphis_marsupialis    .......... .......... .......... .......... ..........
Lasiorhinus_latifrons    .......... .......... .......... .......... ..........
Petaurus_breviceps       .......... .......... .......... .......... ..........
Phascolarctos_cinereus   .......... .......... .......... .......... ..........
Pseudocheirus_peregrinus .......... .......... .......... .......... ..........
Spilocuscus_maculatus    .......... .......... .......... .......... ..........
Trichosurus_vulpecula    .......... .......... .......... .......... ..........
Monodelphis_domestica    .......... .......... .......... .......... ..........

                         3301                                             3350
Aepyprymnus_rufescens    TTGATGAATC CGTTGATTGA TCAATATCTG TACAGATTTG ATAATCTAA.
Dendrolagus_goodfellowi  TTGATGAATC CATTGATTGA TCAATATCTA TATAGATTTG ATAATCTACT
Dendrolagus_matschiei    TTGATGAATC CATTGATTGA TCAATATCTA TATAGATTTG ATAATCTACT
Hypsiprymnodon_moschatus TTGATGAATC CATTGATTGA TCAATATCTA TATAGATTTG ATAATCTAA.
Lagostrophus_fasciatus   TTGATAAATC CATTGATTGA GCAATATCTG TATAGATTCG ATAATCTAA.
Macropus_eugenii         TTGATGAATC CATTGATTGA TCAATATCTG TATAGATTTG ATAATCCAA.
Macropus_parma           CTGATGAATC CATTGATTGA TCAATATCTG TATAGATTTG ATAATCCAA.
Macropus_robustus        TTGATGAATC CATTGATTGA TCAATATCTG TATAGATTTG ATAATCCAA.
Macropus_rufogriseus     TTGATGAATC CATTGATTGA TCAATATCTG TACAGATTTG ATAATCCAA.
Macropus_rufus           TTGATGAATC CATTGATTGA TCAATATCTG TATAGATTTG ATAATCCAA.
Macropus_giganteus       TTGATGAATC CATTGATTGA TCAATATCCG TATAGATTTG ATAATCCAA.
Onychogalea_unguifera    TTGATGAATC CATTGATTGA TCAGTATCTG TATAGATTTG TTAATCCAA.
Petrogale_lateralis      CTGATGAATC CATTGATTGA TCAATATCTG TATAGATTTG ATAATCTAA.
Potorous_tridactylus     TTGATGAATC CATTGATTGA TCAATATCTG TATAAATTTA ATAATCTAA.
Setonix_brachyurus       TTGATGAATC CATTGATTGA TCAATATCTG TATAGATTTG ACAATCCAA.
Thylogale_stigmatica     TTGATGAATC CATTGATTGA TCAATATCTA TATAGATTTG ATAATCTAA.
Didelphis_marsupialis    .......... .......... .......... .......... ..........
Lasiorhinus_latifrons    .......... .......... .......... .......... ..........
Petaurus_breviceps       .......... .......... .......... .......... ..........
Phascolarctos_cinereus   .......... .......... .......... .......... ..........
Pseudocheirus_peregrinus .......... .......... .......... .......... ..........
Spilocuscus_maculatus    .......... .......... .......... .......... ..........
Trichosurus_vulpecula    .......... .......... .......... .......... ..........
Monodelphis_domestica    .......... .......... .......... .......... ..........

                         3351                                             3400
Aepyprymnus_rufescens    CCAGGGGAAA CACTGTTAAT CCCACCTTCA CTTACAAAAA GGGATCAGCA
Dendrolagus_goodfellowi  CCAGTGGAAA CACTGTTAAT CCCACCTTCA CTTACAAAAA GGTATCAGCA
Dendrolagus_matschiei    CCAGTGGAAA CACTGTTAAT CCCACCTTCA CTTACAAAAA GGTATCAGCA
Hypsiprymnodon_moschatus TCAATGGAAA CATTGCTAAT CCCACCTTCA CTTACAAAAA GGAATCAGCA
Lagostrophus_fasciatus   CCAGTGGAAA CACTGTTAAC CCCACCTTCA CTTACAAAAA GGGATCAGCA
Macropus_eugenii         CCTGTGGAAA CACTGTTAAT CCCACCTTCA CTTACAAAAA GGGATCAGCA
Macropus_parma           CCTGTGGAAA CACTGTTAAT CCCACCTTCA CTTTCAAAAA GGGATCAGTA
Macropus_robustus        CTTGTGGAAA CACTGTTAAT CCCACCTTCA CTTACAAAAA GGGATCAGCA
Macropus_rufogriseus     CCTGTGAAAA CACTGTTAAT CCTACCTTCA CTTACAAAAA GGGATCAGCA
Macropus_rufus           CCTGTGGAAA CACTGTTAAT CCCACCTTCA CTTACAAAAA GGGATCAGCA
Macropus_giganteus       CCTGTGGAAA CACTGTTAAT CCCACCTTCA CTTACAAAAA GGGATCAGCA
Onychogalea_unguifera    CCTGTGGAAA CACTGTTAAT CCCACCTTCA CTTACAAAAA GGGATCAGCA
Petrogale_lateralis      CCAGTGGAAA CACTGTTAAT CCCACCTTTA CTTACAAAAA GGTATCAGCA
Potorous_tridactylus     CAAGTGGAAA CACTGATAAT CCCACCTTCA CTT....... ...ATCAGCA
```

```
Setonix_brachyurus       CCTGTGGAAA CACTGTTAAT CCCACCTTCA CTTACAAAAA GGGATCAGCA
Thylogale_stigmatica     CCAGTGGAAA CACTGTTAAT CCCACCTTCA CTTACAAAAA GGTATCAGCA
Didelphis_marsupialis    .......... .......... .......... .......... ..........
Lasiorhinus_latifrons    .......... .......... .......... .......... ..........
Petaurus_breviceps       .......... .......... .......... .......... ..........
Phascolarctos_cinereus   .......... .......... .......... .......... ..........
Pseudocheirus_peregrinus .......... .......... .......... .......... ..........
Spilocuscus_maculatus    .......... .......... .......... .......... ..........
Trichosurus_vulpecula    .......... .......... .......... .......... ..........
Monodelphis_domestica    .......... .......... .......... .......... ..........

                         3401                                              3450
Aepyprymnus_rufescens    GGTGATATGG CTTTTCAGGC TAGGAATTGG TTACCTGGTC CTATGCTTAG
Dendrolagus_goodfellowi  GGTGGTATGG CTTCTCAGGC TAGGAATTGG TTACCTGGTC CTATGCTTAG
Dendrolagus_matschiei    GGTGGTATGG CTTCTCAGGC TAGGAATTGG TTACCTGGTC CTATGCTTAG
Hypsiprymnodon_moschatus GGAGATATGG TTTCTCAGGC TAGGAATTGG TTACC..... .......TAG
Lagostrophus_fasciatus   GGTGATATGG CTTCTCAGGC TAGGAATTGG TTACCTGGTC TTGTGCTTAG
Macropus_eugenii         GGTGATATGG CTTCTCAGGC TAGGAACTGG TTGCCTGGTC CTATGCTTAG
Macropus_parma           GGTGATATGG CTTCTCAGGT TAGGAACTGG TTGCCTGGTC CTATGCTTAG
Macropus_robustus        GGTGATATGT CTTCTCAGGC TAGGAACTGG TTGCCTGGTC CTATGCTTAG
Macropus_rufogriseus     GGTGATATGG CTTCTCAGGC TAGGAACTGG TTGCCTGGTC CTATGCTTAG
Macropus_rufus           GGTGATATGG CTTCTCAGGC TAGGAACTGG TTGCCTGGTC CTATGCTTAG
Macropus_giganteus       GGTGATATGG CTTTTCA... ....AACTGG TTGCCTGGTC CTATGCTTAG
Onychogalea_unguifera    GGTGATATGA CTGCTCAGGC TAGGAACTGG TTGCCTGGTC CTATGCTTAG
Petrogale_lateralis      GGTGATATGG CTTCTCAGGC TAGGAATTGG TTACCTGGTC CTATGCTTAG
Potorous_tridactylus     GGTGATATGG CTTCTCAGGC TAGGAATTGG TTACTTGATC CTATGCTTAG
Setonix_brachyurus       GGTGATATGG CTTCTCAGGC TAGGAACTGG TTGCCTGGTC CTATGCTGAG
Thylogale_stigmatica     GGTGATATGG CTTCTCAGGC TAGGAATTGG TTACCTGGTC CTATGCTTAG
Didelphis_marsupialis    .......... .......... .......... .......... ..........
Lasiorhinus_latifrons    .......... .......... .......... .......... ..........
Petaurus_breviceps       .......... .......... .......... .......... ..........
Phascolarctos_cinereus   .......... .......... .......... .......... ..........
Pseudocheirus_peregrinus .......... .......... .......... .......... ..........
Spilocuscus_maculatus    .......... .......... .......... .......... ..........
Trichosurus_vulpecula    .......... .......... .......... .......... ..........
Monodelphis_domestica    .......... .......... .......... .......... ..........

                         3451                                              3500
Aepyprymnus_rufescens    CAATCAGGGA CTGAAGGATG GTCCTAACAA TCAGGCCAAT CTAGATGGTT
Dendrolagus_goodfellowi  GAATCAGGGA CTAAAGGGTG GTCCTAATAA TCAGGCCAAT CTAGATGGTT
Dendrolagus_matschiei    GAATCAGGGA CTAAAGGGTG GTCCTAATAA TCAGGCCAAT CTAGATGGTT
Hypsiprymnodon_moschatus GGACCAGGGA CTAATGGATG GTCCTAACAA TCAAGTCAGT CTAGATGGTT
Lagostrophus_fasciatus   GAATCAGGGA CTAAAGGATG GTCTTAATAA TCAGACCAAT CTAGATGGTT
Macropus_eugenii         GAATCAGGGA CTAAAGGATG GTCCTAACAA TCAGGCCAAT TTAGATGGTT
Macropus_parma           GAATCAGGGA CTAAAGGATG GTCCTAACAA TCAGGCCAAT TTAGATGGTT
Macropus_robustus        GAATCAGGGA CTAAAGGATG GTCCTAACAA TCAGGCCAAT TTAGATGGTT
Macropus_rufogriseus     GAATCAGGGA CTAAAGGATG GTCCTAACAA TCAGGCCAAT TTAGATGGTT
Macropus_rufus           GAATCAGGGA CTAAAGGATG GTCCTAACAA TCAGGCCAAT TTAGATGGTT
Macropus_giganteus       GAATCAGGGA CTAAAGGATG GTCCTAACAA TCAGGCCAAT TTAGATGGTT
Onychogalea_unguifera    GAATCAGGGA CTAAAGGATG GTCCTAACAA TCAGGCCAAT TTAGATGGTT
Petrogale_lateralis      GAATCAGGGA CTAAAGGGTG GTCCTAATAA TCAGGCCAAT CTAGATGGTT
Potorous_tridactylus     GAATCAGGGA CTAAAGGATG GTCCTAACAA TCAGGCCAAT CTAGATGGTT
Setonix_brachyurus       GAATCAGGGA CTAAAGGATG GTCCTAACAA TCAGGCCAAT TTACATGGTT
Thylogale_stigmatica     GAATCAGGGA CTAAAGGGTG GTCCTAACAA TCAGGCTAGT CTAGATAGTT
Didelphis_marsupialis    .......... .......... .......... .......... ..........
Lasiorhinus_latifrons    .......... .......... .......... .......... ..........
Petaurus_breviceps       .......... .......... .......... .......... ..........
Phascolarctos_cinereus   .......... .......... .......... .......... ..........
Pseudocheirus_peregrinus .......... .......... .......... .......... ..........
Spilocuscus_maculatus    .......... .......... .......... .......... ..........
Trichosurus_vulpecula    .......... .......... .......... .......... ..........
Monodelphis_domestica    .......... .......... .......... .......... ..........
```

```
                           3501                                               3550
Aepyprymnus_rufescens     AGAGGATCGG TCCTTCAACG GTGATCAATG G.AAAATCTT CTATTATATT
Dendrolagus_goodfellowi   GGAGGATCAG TCCTCCAATG GTGATCAATG G.AAAATCTT CTAYTATTTT
Dendrolagus_matschiei     GGAGGATCAG TCCTCCAATG GTGATCAATG G.AAAATCTT CTATTATTTT
Hypsiprymnodon_moschatus  AGAGGATCAG TCCTCTAATG GTGTTCAATG G.AAAATCTT TTACTGTATT
Lagostrophus_fasciatus    GGAGGATCAG TCCTCCAGTG GTGATGAGTG G.AAAATCTT CTATTATATT
Macropus_eugenii          GGAGGATCAG TCCTCCAATG GTGATCAATG G.AAAATCTT CTATTATATT
Macropus_parma            GGAGGATCAG TCCTCCAATG GTGATCAATG G.AAAATCTT CTATTATATT
Macropus_robustus         GGAGGATCAG TCCTCCAATG GTGATCAATG G.AAAATCTT CTATTATATT
Macropus_rufogriseus      GGAGGATCAG TCTTCCAATG GTGATCAATG G.AAAATCTT CTATTATATT
Macropus_rufus            GGAGGATCAG TCCTCCAATG GTGATCAATG G.AAAATCTT CTATTATATT
Macropus_giganteus        GGAGGATCAG TCCTCCAATG GTGATCAATG G.AAAATCTT CTATTATATT
Onychogalea_unguifera     GGAGGATCAT TCCTCCAATG GTGATCAGTG G.AAAATCTT CTATTGCATT
Petrogale_lateralis       GGAGGATCAG TCCTCCAATG GTGATCAATG G.AAAATCTT CTATTATTTT
Potorous_tridactylus      GGAGGATCAG TCCTCCAATG GTGATCAATG GAAAAATCTT CTATTATATT
Setonix_brachyurus        GGAGGATCAG TCCTCCAATG GTGATCAATG G.ATAATCTT CTATTATATT
Thylogale_stigmatica      GAAGGATCAG TCCTCCAATG GTGATCAATG G.AAAATCTT CTATTATTTT
Didelphis_marsupialis     .......... .......... .......... .......... ..........
Lasiorhinus_latifrons     .......... .......... .......... .......... ..........
Petaurus_breviceps        .......... .......... .......... .......... ..........
Phascolarctos_cinereus    .......... .......... .......... .......... ..........
Pseudocheirus_peregrinus  .......... .......... .......... .......... ..........
Spilocuscus_maculatus     .......... .......... .......... .......... ..........
Trichosurus_vulpecula     .......... .......... .......... .......... ..........
Monodelphis_domestica     .......... .......... .......... .......... ..........

                           3551                                               3600
Aepyprymnus_rufescens     TCCTGGGTCA TCCATGTATA CCTCACACAA TGCTGCAGAT GAACTGGAGG
Dendrolagus_goodfellowi   TCCTGGGTCA TCCATGTATA CCGCACAAAA TGTTGCAGAT GAACTGGAGG
Dendrolagus_matschiei     TCCTGGGTCA TCCATGTATA CCGCACAAAA TGTTGCAGAT GAACTGGAGG
Hypsiprymnodon_moschatus  TCCTGGACCA TTCATGTATA CTGCA..... ..TTGCGGAT GAACTGGAGG
Lagostrophus_fasciatus    CCCTGGGCCA TCCATGTATA CTGCCCACAA TGCTGCAAAT GAACTGG...
Macropus_eugenii          TCCTGGGACA TCCATGTGTA CCGCACACAA TGCTGCAGAT GAACTGGAGG
Macropus_parma            TCCTGGGCCA TCCATGTATA CCGCATACAA TGCTGCAGAT GAACTGGCGG
Macropus_robustus         TCCTGGGCCA TCCATGTATA CCGCATACAA TGCTGCAGAT GAACTGGAGG
Macropus_rufogriseus      TCCTGGGCCA TCCATGTATA CCGCACACAA TGCTGCAGAT GAACTGGAGG
Macropus_rufus            TCCTGGGCCA TCCATGTATA CCGCATACAA TGCTGCAGAT GAACTGGAGG
Macropus_giganteus        TCCTGGGCCA TCCATGTATA CCGCACACAG TGCTGCAGAT GAACTGGAGG
Onychogalea_unguifera     TCCTGGGCCA TCCATGTATA CCGCACACAA TGCTGCAGAT GAACTGGAGG
Petrogale_lateralis       TCCTGGGTCA TCCATGTATA CTGCATAAAA TGTTGCAGAT GAACTGGAGG
Potorous_tridactylus      TCCTGGGCCA TCCATGTATA CCTCACACAA TGCTGTAGAT GAACTGGAGG
Setonix_brachyurus        TCCTGGACCA TCCATGTATA CCGCATACAA TGCTGCAGAT GAACTGGAGG
Thylogale_stigmatica      TCCTGGGCCA TCCATGTATA CCACACAAAA TGTTGCAGAT GAACTGGAGG
Didelphis_marsupialis     .......... .......... .......... .......... ..........
Lasiorhinus_latifrons     .......... .......... .......... .......... ..........
Petaurus_breviceps        .......... .......... .......... .......... ..........
Phascolarctos_cinereus    .......... .......... .......... .......... ..........
Pseudocheirus_peregrinus  .......... .......... .......... .......... ..........
Spilocuscus_maculatus     .......... .......... .......... .......... ..........
Trichosurus_vulpecula     .......... .......... .......... .......... ..........
Monodelphis_domestica     .......... .......... .......... .......... ..........

                           3601                                               3650
Aepyprymnus_rufescens     TACAACCTAG CATTAATCTC CCTATCTTTG CTAAAGATGC CTCTATACCT
Dendrolagus_goodfellowi   TTCAACCTAG CATTAATCTC CCTATCTTTG CTAAAGATGC CTCTGTACCT
Dendrolagus_matschiei     TTCAACCTAG CATTAATCTC CCTATCTTTG CTAAAGATGC CTCTGTACCT
Hypsiprymnodon_moschatus  TTCAACCTAG CAGCAATCTC CTCACTTTCG CTAAAGATGC CTCTGTACCT
Lagostrophus_fasciatus    ........AG CATTAATCTC CTTATCTTTG CTAAAGATGC CTCTGTACCT
Macropus_eugenii          TTCAACCTAG CATTAATCTC TCCATCTTTG CTAAAGATGC CTCTGTACCA
Macropus_parma            TTCAACCTAG CATTAATCTC CCCATCTTTG CTAAAGATGC CTCTGTACCT
Macropus_robustus         TTCAACCTAG CATTAATCTC CCCATCTTTG CTAAAGATGC CTCTGTACCT
Macropus_rufogriseus      TTCAACCTAG CATTAATCTC CCCATCTTTG CTAAAGATGC CTCTGTACCG
Macropus_rufus            TTCAACCTAG CATTAATCTC CCCATCTTTG CTAAAGATGC CTCTGTACCT
```

```
Macropus_giganteus       TTCAACCTAG CATTAATCTC CCCATCTTTG CTAAAGATGC CTCTGTACCT
Onychogalea_unguifera    TTCAACCTAG CACTAATCTC CCCACCTTTG CTAAAGATGC CTCTGTACCT
Petrogale_lateralis      TTCAACCTAG CATTAATCTC CCTATCTTTG CTAAAGATGC CTCTGTACCT
Potorous_tridactylus     TACAACCTAG CATTAATCTC CCTATCTTTG CTAAAGATGC CTCTGTACCT
Setonix_brachyurus       TTCAACCTAG CATTAATCTC CCCATCTTTG CTAAAGATGC CTCTGTACCT
Thylogale_stigmatica     TTCAACCTAG CATTAATCTC CCTATCTTTG CTAAAGATGA CTCTGTACCT
Didelphis_marsupialis    .......... .......... .......... .......... ..........
Lasiorhinus_latifrons    .......... .......... .......... .......... ..........
Petaurus_breviceps       .......... .......... .......... .......... ..........
Phascolarctos_cinereus   .......... .......... .......... .......... ..........
Pseudocheirus_peregrinus .......... .......... .......... .......... ..........
Spilocuscus_maculatus    .......... .......... .......... .......... ..........
Trichosurus_vulpecula    .......... .......... .......... .......... ..........
Monodelphis_domestica    .......... .......... .......... .......... ..........

                         3651                                               3700
Aepyprymnus_rufescens    GAATTCTCTA TAATTAGTAG TATTGGTAGT CAAGATCCTA ATAGTAAATT
Dendrolagus_goodfellowi  GAATCCACCA TAATTGGTAG TATTGGTAAT CAAGATCCTA ATAGTAAATT
Dendrolagus_matschiei    GAATCCACCA TAATTGGTAG TATTGGTAAT CAAGATCCTA ATAGTAAATT
Hypsiprymnodon_moschatus GAATCCACCA CAACTAATAG TATTGGTGAT CAAGATCCGA ATAGCAAATT
Lagostrophus_fasciatus   GAGTCCACCA TAATTAGTAG TTTTGGTAAT CAAGATCCTA ATAGTAAATT
Macropus_eugenii         GAATCCACCA TAATTAGTAG TATTGGTAAT CAAGATCCTA ATAGCAAATT
Macropus_parma           GAATCCACCA TAATTAGTAG TATTGGTAAT CAAGATCCTC ATAGTAAATT
Macropus_robustus        GAATCCACCA TAATTAGTAG TATTGGTAAT CAAGATCTTA ATAGTAAATT
Macropus_rufogriseus     GAATCCACCA TAATTAGTAG TATTGGTAAT CAGAATCCTA ATAGTAAATT
Macropus_rufus           GAATCCACCA TAATTAGTAG TATTGGTAAT CAAGATCCTA ATAGTAAATT
Macropus_giganteus       GAATCCACCA TAATTAGTAG TATTGGTAAT CAAGATCCTA ATAGTAAATT
Onychogalea_unguifera    GAATCCACCT TAATTAGTAG TATTGATAAT CAAGATCCTA ATAGTAAA.T
Petrogale_lateralis      GAATCCACCA TAATTGGTAG TATTGGTAAT CAAGATCCTA ATAGTAAATT
Potorous_tridactylus     GAATCCACCA TAATTAGTAG CATTGGTAAT CAAGATTCTA ATAGTAAATT
Setonix_brachyurus       GAATCCACCA TAATTAGTAG TATTGCTAAT CAAGATCCTA ATAGTAAATT
Thylogale_stigmatica     GAATCCACCA TAATTGGTAG TATTGGTAAT CAAGATCCTA ATAGTAAATT
Didelphis_marsupialis    .......... .......... .......... .......... ..........
Lasiorhinus_latifrons    .......... .......... .......... .......... ..........
Petaurus_breviceps       .......... .......... .......... .......... ..........
Phascolarctos_cinereus   .......... .......... .......... .......... ..........
Pseudocheirus_peregrinus .......... .......... .......... .......... ..........
Spilocuscus_maculatus    .......... .......... .......... .......... ..........
Trichosurus_vulpecula    .......... .......... .......... .......... ..........
Monodelphis_domestica    .......... .......... .......... .......... ..........

                         3701                                               3750
Aepyprymnus_rufescens    GCTAGTCACT GATG.AGAAT TAGGTCGGGA CA.TGAATGC TGTTGCTGCT
Dendrolagus_goodfellowi  GTTAGTCACT GATG.AGAAT GAGGTCRGGA CAGTGAATGC TACTGCTGCT
Dendrolagus_matschiei    GTTAGTCGCT GATG.AGAAT GAGGTCGGGA CAGTGAATGC TACTGCTGCT
Hypsiprymnodon_moschatus GTTAGTCACT GATG.AGAAG AAGGTCAGGA CAGTGAATGC TACTGCTGCT
Lagostrophus_fasciatus   GTTAGTCACT GATG.AGAAC GAGGTCAGGA CAGTGAATGC TACTGCTGCT
Macropus_eugenii         GTTAGTCACT GATG.AGAAC GAGGTCGGGA CAGTGAATGC TACTGCTGCT
Macropus_parma           GTTAGTCACT GATGAAGAAC GAGGTCGGGA AAGTAAATGC TACTGCTGCT
Macropus_robustus        GTTAGTCACT AATG.AGAAC GAGGTCGGGA AAGTGAATGC TACTGCTGCT
Macropus_rufogriseus     GTCAGTCACT GATG.AGAAC GAGGTCGGGA CAGTGAATGC TACTGCTGCC
Macropus_rufus           GTTAGTCACT AATG.AGAAC AAGGTCGGGA AAGTGAATGC TACTGCTGCT
Macropus_giganteus       GTTAGTCACT GATG.AGAAC GAGGTCGGGA CAGTGAATGC TACTGCTGCT
Onychogalea_unguifera    GTTAGTCACT GATG.AGAAC GAGGTCAGGA CAGTGAATGC TACTGCTGTT
Petrogale_lateralis      GTTAGTCACT GATG.AGAAT GAGGTCGGGA CAGTGAATGC TACTGCTGCT
Potorous_tridactylus     GTTAGTCACT GATG.GCAAC GAGGTTGGGA CAGTGAATGC TACTGCTGCT
Setonix_brachyurus       GTTAGTCACT GATG.AGAAT GAGGTCGGGA CAGTGAATGC TACTGCTGCT
Thylogale_stigmatica     GTTAGTCACT GATG.AGAAT GAGGTCGGGA CAGTGAATGC TACTGCTGCT
Didelphis_marsupialis    .......... .......... .......... .......... ..........
Lasiorhinus_latifrons    .......... .......... .......... .......... ..........
Petaurus_breviceps       .......... .......... .......... .......... ..........
Phascolarctos_cinereus   .......... .......... .......... .......... ..........
Pseudocheirus_peregrinus .......... .......... .......... .......... ..........
```

```
Spilocuscus_maculatus     .......... .......... .......... .......... ..........
Trichosurus_vulpecula     .......... .......... .......... .......... ..........
Monodelphis_domestica     .......... .......... .......... .......... ..........

                          3751                                              3800
Aepyprymnus_rufescens     AATACCTGGG GGCTTATGGC AGTCCATCAG CAGACTCCCA CCCCTACTAG
Dendrolagus_goodfellowi   ATTACCTGGG GGTTTATGGT AGTCAACCAG CAGACTCCCA CCCCCACTAC
Dendrolagus_matschiei     ATTACCTGGG GGTTTATGGT AGTCAACCAG CAGACTCCCA CCCCCACTAC
Hypsiprymnodon_moschatus AATACCCGGG GATCTGTGAC AGTGAACCAG AAGA.TCTCA CCTCCACTAC
Lagostrophus_fasciatus    AACACCTAGG GGTCTATGGC AGTCAATCAG CAGACTCCCA CCCCCACTAG
Macropus_eugenii          AATACCTGGG GGTCTATGGC AGTCAACCAG CAGACTCCCA CCCCCACTAG
Macropus_parma            AATACCTGGG GGTCTATAGC AGTCAACCAG CAGACTCCCA CCCCCACTAG
Macropus_robustus         AATACCTGGG GGTCTATGGC AGTCAACCAG CAGTCTCCCA CCCCCACTAG
Macropus_rufogriseus      AATACTTGGG GGTCTATGGC AGTCAACCAG CAGACTCCCA CCCCGACTAG
Macropus_rufus            AATACCTGGG GGTCTATGGC AGTCAACCAG CAGTCTCCCA CCCCCACTAG
Macropus_giganteus        AATACCTGGG GGTCTATGGC AGTCAACCAG CAGACTCCCA CCCCCACTAG
Onychogalea_unguifera     AATACCTGGG GGTCTATGGC AGTCAACCAG CAGACTCCCA CCCCCACTAG
Petrogale_lateralis       ATTACCTGGG RATTTATGGC AGTCAACCAG CAGACTCCCA CCCCCACTAC
Potorous_tridactylus      AATACCTGAG GGTTTATGGC AGTCAACCAG CAGACTCCCA CTCCCACTAG
Setonix_brachyurus        AATACCTGGG GGTCTATGTC AGTCAACCAG CAGACTCCCA CCCCCACTGG
Thylogale_stigmatica      ATTACCTGGG GGTTTATGGC AGTCAACCAG CAGACTCCCA CCCCCACTAC
Didelphis_marsupialis     .......... .......... .......... .......... ..........
Lasiorhinus_latifrons     .......... .......... .......... .......... ..........
Petaurus_breviceps        .......... .......... .......... .......... ..........
Phascolarctos_cinereus    .......... .......... .......... .......... ..........
Pseudocheirus_peregrinus .......... .......... .......... .......... ..........
Spilocuscus_maculatus     .......... .......... .......... .......... ..........
Trichosurus_vulpecula     .......... .......... .......... .......... ..........
Monodelphis_domestica     .......... .......... .......... .......... ..........

                          3801                                              3850
Aepyprymnus_rufescens     TGCAGGATAG GTTCTAAATC AAATGAGTGT CATACCTGGA ATAGTCTGGC
Dendrolagus_goodfellowi   TGCAGGATAG GTTCTAAATC AAATGAGTGT CATGCCTGGA ATGATCTGGC
Dendrolagus_matschiei     TGCAGGATAG GTTCTAAATC AAATGAGTGT CATGCCTGGA ATGATCTGGC
Hypsiprymnodon_moschatus TGCTGGACAG GTTCTAAATC AAATGAGTGC CATGCCTGGA ATGGTCTGCC
Lagostrophus_fasciatus    TGCAGGACAA GTTCTAAATC AAATGAGTGT CATGCCTGGA ATGGTCTGGC
Macropus_eugenii          TGTAGGACAG GTTCTAAATC AAATGAGTGT CATGCCTGGA ATGGTCTGGC
Macropus_parma            TGCAGGACAG GTTCTAAATC AAATGAGTGT CATGCCTGGA ATGGTCTGGC
Macropus_robustus         TGCA...... ...CTAAATC AAATGAGTGT CATGCCTGGA ATGGTCTGGC
Macropus_rufogriseus      TGCAGGACAG GTTCTAAATC AAATGAGTGT CATGCCTGGA ATGGTCTGGC
Macropus_rufus            TGCA...... ...CTAAATC AAATGAGTGT CATGCCTGGA ATGGTCTGGC
Macropus_giganteus        TGCAGGACAG GTTCTAAATC AAATGAGTGT CATGCCTGGA ATGGTCTGGC
Onychogalea_unguifera     TGCAGGACAG GTTCTAAATC AAATGAGTGT CATGCCTGGA ATGGTCTGGC
Petrogale_lateralis       TGCAGGATAG TTTCTAAATC AAATGAGTGT CATGCCTGGA ATGATCTGGC
Potorous_tridactylus      TGGAGGATAG GTTCTAAATC AAATGAGTGT CATGCCTGGA ATGGTCTGGC
Setonix_brachyurus        TGCAGGACAG GTTCTAAATC AAATGAGTGT CATGCCTGGA ATGGTCTGGC
Thylogale_stigmatica      TGCAGGATAG GTTTTAAATC AAATGAGTGT CATGCCTGGG ATGATCTGGC
Didelphis_marsupialis     .......... .......... .......... .......... ..........
Lasiorhinus_latifrons     .......... .......... .......... .......... ..........
Petaurus_breviceps        .......... .......... .......... .......... ..........
Phascolarctos_cinereus    .......... .......... .......... .......... ..........
Pseudocheirus_peregrinus .......... .......... .......... .......... ..........
Spilocuscus_maculatus     .......... .......... .......... .......... ..........
Trichosurus_vulpecula     .......... .......... .......... .......... ..........
Monodelphis_domestica     .......... .......... .......... .......... ..........

                          3851                                              3900
Aepyprymnus_rufescens     AGAATAGAGA CATCGATCTC CATGGTCCTA TTTGGGTTAA GATTTCTCAC
Dendrolagus_goodfellowi   AGAATAGAGA CATTGATCTC CATAGTCCCA TTTGGGCTAA GATTCCTCAC
Dendrolagus_matschiei     AGAATAGAGA CATTGATCTC CATAGTCCCA TTTGGGCTAA GATTCCTCAC
Hypsiprymnodon_moschatus AAAATAGAGA C....ACCTC CATGGTCCTA TTTGGGTGAA GATTCCTCAC
Lagostrophus_fasciatus    AGAATAGAGA CATTGATCTC CATGGTTCCG TTTGGGCTAA GATTCCTCAC
Macropus_eugenii          AGAAAAGAGA CATCAATCTC CATGGTCCCA TTTGGGCTAA GATTCCTCAC
```

```
Macropus_parma           AGAACAGAGA CATCAATCTC CATGGTCCCA TTTGGGCTAA GATTCCTCAC
Macropus_robustus        AGAATAGAAA CATCAATCTC CATGGTCCCA TTTGGGCTAA GATTCCTCAC
Macropus_rufogriseus     AGAATAGAGA CATCAATCTC CATGGTCCCA TTTAGGCTAA GATTCCTCAC
Macropus_rufus           AGAATAGAAA CATCAATCTC CATGGTCCCA TTTGGGCTAA GATTCCTCAC
Macropus_giganteus       AGAATAGAGA CATCAATCTC CATGGTCCCA TTTGGGCTAA GATTCCTCAC
Onychogalea_unguifera    AGAATAGAGA CATCAATCTC TATGGTCCCA TTTGGGCTAA GATTCCTCAC
Petrogale_lateralis      AGAATAGAGA CATTGATCTC CATAGTCCCA TTTGGGCTAA GATTCCTCAC
Potorous_tridactylus     AGAACAGAGA CATCGATCTC CATGATCCCA TTTTGGCTAA GATTCCTCAC
Setonix_brachyurus       AGAATAGAGA CATCAATCTC CATGGTCCCA TTTGGGCTAA GATTCCTCAC
Thylogale_stigmatica     AGAATAGAGA CATTGATCTC CATAGTCCCA TTTGGGCTAA GATTCCTCAC
Didelphis_marsupialis    .......... .......... .......... .......... ..........
Lasiorhinus_latifrons    .......... .......... .......... .......... ..........
Petaurus_breviceps       .......... .......... .......... .......... ..........
Phascolarctos_cinereus   .......... .......... .......... .......... ..........
Pseudocheirus_peregrinus .......... .......... .......... .......... ..........
Spilocuscus_maculatus    .......... .......... .......... .......... ..........
Trichosurus_vulpecula    .......... .......... .......... .......... ..........
Monodelphis_domestica    .......... .......... .......... .......... ..........

                         3901                                               3950
Aepyprymnus_rufescens    ACAGATGGTT ACTTCCATCC CTCTCCTCCC ATGGGTGTCT TTGGTCTCAA
Dendrolagus_goodfellowi  ACAGATGGTT ACTTCCATCC CTCTCCTCTC ATGGGTGGCT TTGGTCTCAA
Dendrolagus_matschiei    ACAGATGGTT ACTTCCATCC CTCTCCTCTC ATGGGTGGCT TTGGTCTCAA
Hypsiprymnodon_moschatus ACAGATCGTC ACTTCCATCC CTCTCCTCTA ATGGGTGGCT TTGGTCTCAA
Lagostrophus_fasciatus   ACAGATGGTT ACTTCCATCC CTCTCCTCTC ATGGGTGCCT TTGGTCTCAA
Macropus_eugenii         ACAGATGGTT ACTTCCATCC CTCTCCTCTT ATGGGTGGCT TTGGTCTCAA
Macropus_parma           ACAGATGGTT ACTTCCATCC CTCTCCTCTT ATGGATGGCT TTGGTCTCAA
Macropus_robustus        ACAGATGGTT ACTTCCATCC CTCTCCTCTT ATGGGTGGCT TTGGTCTCAA
Macropus_rufogriseus     ACAGATGGTT ACTTCCATCC CTCTCCTCTT ATGGGTGGCT TTGGTCTCAA
Macropus_rufus           ACAGATGGTT ACTTCCATCC CTCTCCTCTT ATGGGTGGCT TTGGTCTCAA
Macropus_giganteus       ACAGATGGTT ACTTCCATCC CTCTCCTTTT ATAGGTGGCT TTGGTCTCAA
Onychogalea_unguifera    ACAGATGGTT ACTTCCATCC CTCTCCTCTT ATGGGTGGCT TTGGTCTCAA
Petrogale_lateralis      ACAGATGGTT ACTTCCATCC CTCTCCTCTC ATGGGTGGCT TTGGTCTCAA
Potorous_tridactylus     ACAGATGGTT ACTTCCATCC CTCTCCTCTC ATGGGTGGCT TTGGTCTCAA
Setonix_brachyurus       ACAGATGGTT ACTTCCATCC CTCTCCTCTT ATGGATGGCT TTGGTCTCAA
Thylogale_stigmatica     ACAGATGGTT ACTTCCATCC CTCTCCTCTC ATGGGTGGCT TTGGTCTCAA
Didelphis_marsupialis    .......... .......... .......... .......... ..........
Lasiorhinus_latifrons    .......... .......... .......... .......... ..........
Petaurus_breviceps       .......... .......... .......... .......... ..........
Phascolarctos_cinereus   .......... .......... .......... .......... ..........
Pseudocheirus_peregrinus .......... .......... .......... .......... ..........
Spilocuscus_maculatus    .......... .......... .......... .......... ..........
Trichosurus_vulpecula    .......... .......... .......... .......... ..........
Monodelphis_domestica    .......... .......... .......... .......... ..........

                         3951                                               4000
Aepyprymnus_rufescens    ACATCCTCCT CCTCAGAATA TGATTAAAAA CACTCCTGTC TCTGC.TAAC
Dendrolagus_goodfellowi  ACATCCTCTT CCTCAGAATA TGATTAAAAA CTCTCCTGTC CCTGC.TAAC
Dendrolagus_matschiei    ACATCCTCTT CCTCAGAATA TGATTAAAAA CTCTCCTGTC CCTGC.TAAC
Hypsiprymnodon_moschatus ACATCTTCCT CCTCAGATTA TGATTAAAAA CACTCCTGTC CCTCC.TAAC
Lagostrophus_fasciatus   ACGTCCTCCT CCTCAGAATA TGATTAAAAA TACTCCTGTC CCTGC.TAAC
Macropus_eugenii         ACAATCTCCT CCTCAGAATA TGATTAAAAA CTCTCCTGTC CCTGC.TAAC
Macropus_parma           ACATCCTCCT CCTCAGAATA TGATTAAAAA CTCTCCTGTC CCTGC.TAAC
Macropus_robustus        ACATCCTCCT CCTCAGAATA TGATTAAAAA CTCTCCTGTC CCTGC.TAAC
Macropus_rufogriseus     ACATCCTTTT CCTCAGAATA TGATTAAAAA CTCTCCTGTC CCTGC.TAAC
Macropus_rufus           ACATCCTCCT CCTCAGAATA TGATTAAAAA CTCTCCTGTC CCTGC.TAAC
Macropus_giganteus       ACATCCTCCT CCTCAGAATA TGATTGAAAA CTCTCCTGTC CCTGC.TAAC
Onychogalea_unguifera    ACATCCTCCT CCTCAGAATA TGATTAAAAA CTCTCCTGTC CCTGC.TAAC
Petrogale_lateralis      ATATCCTCTT CCTCAGAATA TGATTAAAAA CTCTCCTGTT CCTGC.TAAC
Potorous_tridactylus     GCATCCTCCT CCTCAGAATA TGATTAAAAA CACTCCTGTC CCTACTTAAC
Setonix_brachyurus       ACATCCTCCT CCTCAGAATA TGATTAAAAA CTCTCCTGTC CCTGC.TAAC
Thylogale_stigmatica     ACATCCTCTT TCTCAGAATA TGATTAAAAA CTCTCCTGTC CCTGC.TAAC
Didelphis_marsupialis    .......... .......... .......... .......... ..........
```

```
Lasiorhinus_latifrons     .......... .......... .......... .......... ..........
Petaurus_breviceps        .......... .......... .......... .......... ..........
Phascolarctos_cinereus    .......... .......... .......... .......... ..........
Pseudocheirus_peregrinus  .......... .......... .......... .......... ..........
Spilocuscus_maculatus     .......... .......... .......... .......... ..........
Trichosurus_vulpecula     .......... .......... .......... .......... ..........
Monodelphis_domestica     .......... .......... .......... .......... ..........

                          4001                                              4050
Aepyprymnus_rufescens     CCTGCCACCA TCTTCACTCC TGTCAAACAA AATTCTTTCA TCATTCAATA
Dendrolagus_goodfellowi   CCTGCCACCA TCTTCACTYC TGTCAAACAA AACTCTTTCA TCACTCAATA
Dendrolagus_matschiei     CCTGCCACCA TCTTCACTCC TGTCAAACAA AACTCTTTCA TCACTCAATA
Hypsiprymnodon_moschatus  CCTGCCACCA CCTTCACTCC TGTCAAACAA AACTCTTTCA TCACTTAATA
Lagostrophus_fasciatus    CCTGCCACCA TCTTCACTCC TGTCAAACAA AATTCTTTCA TCACTCAATA
Macropus_eugenii          CCCACCACCA TCTTCACTCT TGTCAAACAA AACTCTTTTA TCACTCAATA
Macropus_parma            CCCACCACCA TCTTCACTCC TGTCAAACAA AACTCTTTCA TTACTCAATA
Macropus_robustus         CCCACCACCA TCTTCACTCC TGTCAAACAA AACTCTTTCA TCACTCAATA
Macropus_rufogriseus      CCCACCACCA TCTTCACTCC TGCCAAACAA CACTCTTTCA TCACTCAATA
Macropus_rufus            CCCACCACCA TCTTCACTCC TGTCAAACAA AACTCTTTCA TCACTCAATA
Macropus_giganteus        CCCACCACCA TCTTCACTCC TGTCAAACAA AACTCTTTTA TCACTCAATA
Onychogalea_unguifera     CCCACCACCA TCTTCACTCC TCTCAAACAA AACTCTTTCA TCACTCAATA
Petrogale_lateralis       CCTGCCACCA TCTTCACTCC TGTCAAACAA AACTCTTTCA TCACTCAATA
Potorous_tridactylus      CCTGCCACTG TCTTCACTCC TGTCAAACAA AATTCTTTCA TCACTCAATA
Setonix_brachyurus        CCCACCATCA TCTTCACTCC TGTCAAACAA AACTCTTTCA TCACTCAATA
Thylogale_stigmatica      CCTGCCACCA TCTTCACTCC TGTCAAACAA AACTCTTTCA TCACTCAATA
Didelphis_marsupialis     .......... .......... .......... .......... ..........
Lasiorhinus_latifrons     .......... .......... .......... .......... ..........
Petaurus_breviceps        .......... .......... .......... .......... ..........
Phascolarctos_cinereus    .......... .......... .......... .......... ..........
Pseudocheirus_peregrinus  .......... .......... .......... .......... ..........
Spilocuscus_maculatus     .......... .......... .......... .......... ..........
Trichosurus_vulpecula     .......... .......... .......... .......... ..........
Monodelphis_domestica     .......... .......... .......... .......... ..........

                          4051                                              4100
Aepyprymnus_rufescens     CTCTACTGGT CAAGTGACTG TAGAAATTG. .......... ....AGGAAA
Dendrolagus_goodfellowi   CTCTATTGGT CAAGTGACTG TAGAAATTGA ATGGGAACTC CATAAGSAAA
Dendrolagus_matschiei     CTCTATTGGT CAAGTGACTG TAGAAATTGA ATGGGAACTC CATAAGGAAA
Hypsiprymnodon_moschatus  CTCTACTGGT CAAGTGACTG TAGAAATTGA ATGGGAACTC CAGAAAGAAA
Lagostrophus_fasciatus    GTCTACTGGT CAAGTGACTG TAGAAATTGA ATGGGAACTC CATAAGGAAA
Macropus_eugenii          CTCTATTGGT CAAGTGACTG TAGAAATTGA ATGGGAACTC CATAAGGAAA
Macropus_parma            CTCTATTGGT CAAGTGACTG TAGAAATTGA ATGGAAACTC CATAAGGAAA
Macropus_robustus         CTCTATTGGT CAAGTGACTG TAGAAATTGA ATGGGAACTC CATAAGGAAA
Macropus_rufogriseus      CTCTATTGGT CAAGTGACTG TAGAAATTGA ATGGGAACTC CATAAGGAAA
Macropus_rufus            CTCTATTGGT CAAGTGACTG TAGAAATTGA ATGGGAACTC CATAAGGAAA
Macropus_giganteus        CTCTATTGGT CTAGTGACTG TAGAAATTGA ATGGGAACTC CATAAGAAAA
Onychogalea_unguifera     CTCTATTGGT CAAGTGACTG TAGAAATTGA ATGGGAACTC CATAAGGAAA
Petrogale_lateralis       CTCTATTGGT CAAGTGACTG TAGAAATTGA ATGGGAACTC CATAAGGAAA
Potorous_tridactylus      CTCTACTGGT CAAGTGACTG TAGAAATTGA ATGGGAACTT CATAAGGAAA
Setonix_brachyurus        CTCTATTGGT CAAGTGACTG TAGAAATTGA ATGGGAACTC CATAAGGACA
Thylogale_stigmatica      CTGTATTGGT CAAGTGACTG TAGAAATTGA ATGGGAACTC CATAAGGAAA
Didelphis_marsupialis     .......... .......... .......... .......... ..........
Lasiorhinus_latifrons     .......... .......... .......... .......... ..........
Petaurus_breviceps        .......... .......... .......... .......... ..........
Phascolarctos_cinereus    .......... .......... .......... .......... ..........
Pseudocheirus_peregrinus  .......... .......... .......... .......... ..........
Spilocuscus_maculatus     .......... .......... .......... .......... ..........
Trichosurus_vulpecula     .......... .......... .......... .......... ..........
Monodelphis_domestica     .......... .......... .......... .......... ..........

                          4101                                              4150
Aepyprymnus_rufescens     GTTCCAAGAA ATGGAATCCT GAAATCCAGT TCACTTCCAA TTTCAGAAAC
Dendrolagus_goodfellowi   GTTCCAAGAA ATGGAATCCT GAAATCCAGT TTACTTCCAA TTTCAGAAAC
```

```
Dendrolagus_matschiei    GTTCCAAGAA ATGGAATCCT GAAATCCAGT TTACTTCCAA TTTCAGAAAC
Hypsiprymnodon_moschatus CCTCCTAGAA ATGGAATACT GAAATCCACT TTATTTCCAC TTTCAGAAAC
Lagostrophus_fasciatus   GTTCCAAGAA ATGAACTCCT GAAATCCAGC TTACTTCCAG TTTCAGAAAC
Macropus_eugenii         GTTCCAAGAA ATGGACTCCT GAAATCCATT TTACTTCCAA TTTCAGAAAC
Macropus_parma           GTTCCAAGAA ATGGACTCCT AAAATCCAGT TTACTTCCAA TTTCAGAAAC
Macropus_robustus        GTTCCAAGAA ATGGACTCCT AAAATCCAGT TTACTTCCAA TTTCAGAAAC
Macropus_rufogriseus     GTTCCAAGAA ATGGACTCCT GAAATCCAGT TTACTTCCAA TTTCAGAAAC
Macropus_rufus           GTTCCAAGAA ATGGACTCCT AAAATCCAGT TTACTTCCAA TTTCAGAAAC
Macropus_giganteus       GTTCCAAGAA ATGGACTCCT GAAATCCAGT TTACTTCCAA TTTCAGAAAC
Onychogalea_unguifera    GTTCCAGGAA ATGGACTCCT GAAATCCAGT TTACTTCCAA TTTCAGAAAC
Petrogale_lateralis      GTTCCAAGAA ATGGAATCTT GAAATCCAGT TTACTTCCAA TTTCAGAAAC
Potorous_tridactylus     GTTCCAAGAA ATGAACTCCT GAAATCCAGT TTACTTCCAG TTTCAGAAAT
Setonix_brachyurus       GTTCCAAGAA ATGGACTCCT AAAATCCAGT TTAC...... TTTCAGAAAC
Thylogale_stigmatica     GTTCCAAGAA ATGGAATCTT GAAATCCAGT TTACTTCCAA TTTCAGAAAC
Didelphis_marsupialis    .......... .......... .......... .......... ..........
Lasiorhinus_latifrons    .......... .......... .......... .......... ..........
Petaurus_breviceps       .......... .......... .......... .......... ..........
Phascolarctos_cinereus   .......... .......... .......... .......... ..........
Pseudocheirus_peregrinus .......... .......... .......... .......... ..........
Spilocuscus_maculatus    .......... .......... .......... .......... ..........
Trichosurus_vulpecula    .......... .......... .......... .......... ..........
Monodelphis_domestica    .......... .......... .......... .......... ..........

                         4151                                              4200
Aepyprymnus_rufescens    ACCACTGACT TACCTTTTGC TCCCAACAAT GAAGGTGTAT ACTCTGAACC
Dendrolagus_goodfellowi  ACTATTGACT TACCTTTTGC TCCTAACAAT GAAGGTGCAT ACTCTGAACC
Dendrolagus_matschiei    ACTATTGACT TACCTTTTGC TCCTAACAAT GAAGGTGCAT ACTCTGAACC
Hypsiprymnodon_moschatus ACTATTGACT TACCTTTTGC TCCCAACAAT GAAGATGTGT ACTCTGAACC
Lagostrophus_fasciatus   ATTATTGACT TACCCTTTGC TCCCAACAAT GAAGGTGTAT ACTCTGAACC
Macropus_eugenii         ACTATTGACT TATCTTTTGC TCCCAACAAT GAAGGTGTAT ACTGTAAACC
Macropus_parma           ACTATTGACT TACCTTTTGC TCCCAACAAT GAAGGTGTAT ACTATGAACC
Macropus_robustus        ACTATTGACT TACCTTTTGC TCCCAACAAT GAAGGTGTAT ACTATGAACC
Macropus_rufogriseus     ACTATTGACT TACCTTTTGC TCCCAACAAT GAAGGTGTAT ACTATGAACC
Macropus_rufus           ACTATTGACT TACCTTTTGC TCCCAACAAT GAAGGTGTAT ACTATGAACC
Macropus_giganteus       ACTATTGACT TACCTTTTGC TCCCAACAAT GAAGGTGTAT ACTGTGAACC
Onychogalea_unguifera    ACTATTGACT TACCTTTCGC TCCCAACAAT GAAGGTGTAT ACTGTGAACC
Petrogale_lateralis      ACTATTGACT TACCTTTTGC TCCCAACAAT GAAGGTGTAT ACTCTGAACC
Potorous_tridactylus     ACTATTAACT .....TTTGT TCCCAACAAT GAAGGTGTAT ACTCTGAACC
Setonix_brachyurus       ACTATTGACT TACCTTTTGC TCCCAACAAT GAAGGTGTAT ACTATGAACC
Thylogale_stigmatica     ACTATTGACT TACCTTTTGC TCCCAACAAT GAAGGTGTAT ACTCTGAACC
Didelphis_marsupialis    .......... .......... .......... .......... ..........
Lasiorhinus_latifrons    .......... .......... .......... .......... ..........
Petaurus_breviceps       .......... .......... .......... .......... ..........
Phascolarctos_cinereus   .......... .......... .......... .......... ..........
Pseudocheirus_peregrinus .......... .......... .......... .......... ..........
Spilocuscus_maculatus    .......... .......... .......... .......... ..........
Trichosurus_vulpecula    .......... .......... .......... .......... ..........
Monodelphis_domestica    .......... .......... .......... .......... ..........

                         4201                                              4250
Aepyprymnus_rufescens    TCATCCCATT GGTACCTGAT ACCTTACCCT TCCCATGTAA CTGTATTGTA
Dendrolagus_goodfellowi  TCGTCCCATT GGTACTYGAT ACCTTACCCT TCCCATCTAA CTGTATTGCA
Dendrolagus_matschiei    TCGTCCCATT GGTACTCGAT ACCTTACCCT TCCCATCTAA CTGTATTGCA
Hypsiprymnodon_moschatus TTGTCTCATT GGTACCGGAT GCCTTACCTG TCCCATCTAT CTGTGTTGTA
Lagostrophus_fasciatus   TCATTCCATT GGTACCCAAT ACCTTACCC. TCCCATCTAA CTGTATTGTA
Macropus_eugenii         TCGTCCCATT GGTACCTGAT ACCTTACCCT TCCCATCTAA CTGTATTGCA
Macropus_parma           TCGTCCCATT GGTACCTGAT ACCTTACCCT TCCCATCTAA CTGTATTGCA
Macropus_robustus        TCGTCCCATT GCTACCTGAT ACCTTACCCT TCCCATCGAA CTGTATTGCA
Macropus_rufogriseus     TTGTCCCATT GGTACCTGAT ACCTTACCCT TCCCATCTAA CTGTATTGTA
Macropus_rufus           TCGTCCCATT GGTACCTGAT ACCTTACCCT TCCCATCGAA CTGTATTGCA
Macropus_giganteus       TCATCCCATT GGTACCTGAT ACCTTACCCT TCCCATCTAA CTGTATTGCA
Onychogalea_unguifera    TCATCCCATT GGTACCTGAT ATCTTACCCT TCCCATCTAA CTGTATCGCA
Petrogale_lateralis      TCATCCCAYT GGTACTCGAT ACCTTACCCT TCCCATCTAA CTGTATTGCA
```

```
Potorous_tridactylus      TCGTCTTATT GGTACCCGAT ACTTTACTC. TCCCATCTAA CTGTATTGTA
Setonix_brachyurus        TCGTCCCATT GGTACCTGAT ACCTTACCCT TCCCATCTAA CTGTATTGCA
Thylogale_stigmatica      TCGTCCCATT GGTACTCGAT ACGTTACCCT TCCCATCTAA CTGTATTGCA
Didelphis_marsupialis     .......... .......... .......... .......... ..........
Lasiorhinus_latifrons     .......... .......... .......... .......... ..........
Petaurus_breviceps        .......... .......... .......... .......... ..........
Phascolarctos_cinereus    .......... .......... .......... .......... ..........
Pseudocheirus_peregrinus  .......... .......... .......... .......... ..........
Spilocuscus_maculatus     .......... .......... .......... .......... ..........
Trichosurus_vulpecula     .......... .......... .......... .......... ..........
Monodelphis_domestica     .......... .......... .......... .......... ..........

                          4251                                               4300
Aepyprymnus_rufescens     CATATTTCAT ATTTGTA.TT TTTTATTCAA TAAACTGATT TATTCATTTC
Dendrolagus_goodfellowi   CATATTTCAT ATTTGTA.TT TTTTATTCAA TAAACTGATT TATTCATTTC
Dendrolagus_matschiei     CATATTTCAT ATTTGTG.TT TTTTATTCGA TAAACTGATT TATTCATTTC
Hypsiprymnodon_moschatus  CTTATTTTAT ATTTGTACTT TTTTATTCAA TAAACAGGTT TATTCGTTTC
Lagostrophus_fasciatus    TATACTTCAT GTTTGTATTT TTTTATTCAA TAAACTAATT TATTCATTTC
Macropus_eugenii          CATATTTCAT GTTTGTATTT TTTTATTCAA TAAACTGATG TATTCATTTC
Macropus_parma            CATATTTCAT ATTTGTATTT TTTTATTCAA TAAACTGATA TATTCATTTC
Macropus_robustus         CATATTTCAT ATTTGTATTT TTTTATTCAA TAAACTGATG TATTCATTTC
Macropus_rufogriseus      CATATTTCAT ATTTGTATTT TTTTATTCAA TAAACTGATG TATTCATTTC
Macropus_rufus            CATATTTCAT ATTTGTATTT TTTTATTCAA TAAACTGATG TATTCATTTC
Macropus_giganteus        CATATTTCAT ATTTGTATTT TTTTATTCAA TAAACTGATG TATTCATTTC
Onychogalea_unguifera     CATATTTCAT ATCTGTATTT TTTTATTCAA CAAACTGATG TATTAATTTC
Petrogale_lateralis       CATATTTCAT ATTTGTA.TT TTTTATTCAA TAAACTGATT TATTCATTTC
Potorous_tridactylus      TATATTTCAT ATTTGTATTT TTTTATTCAA TAAACTGATT TATTCATTTC
Setonix_brachyurus        CATATTTCAT ATTTGTATTT TTTTATTCAA TAAACTGATG TATTCATTTC
Thylogale_stigmatica      CATATTTCAT ATTTGTA.TT TTTTATTCAA TAAACTGATT TATTCATTTC
Didelphis_marsupialis     .......... .......... .......... .......... ..........
Lasiorhinus_latifrons     .......... .......... .......... .......... ..........
Petaurus_breviceps        .......... .......... .......... .......... ..........
Phascolarctos_cinereus    .......... .......... .......... .......... ..........
Pseudocheirus_peregrinus  .......... .......... .......... .......... ..........
Spilocuscus_maculatus     .......... .......... .......... .......... ..........
Trichosurus_vulpecula     .......... .......... .......... .......... ..........
Monodelphis_domestica     .......... .......... .......... .......... ..........

                          4301                                               4350
Aepyprymnus_rufescens     ATTGTACTTC TCTTGGGATG TGGCTCTTAT AAGCAGATGA TGAGCTGCTG
Dendrolagus_goodfellowi   ATTGTATTTC TCTTATCACT TGGCTCTTAT AAGCAGASGA TGAGCTGCTG
Dendrolagus_matschiei     ATTGTATTTC TCTTATCACT TGGCTCTTAT AAGCAGACAA TGAGCTGCTG
Hypsiprymnodon_moschatus  ATTGTACTTC TCTTGTCACG TGGTGCTTAT AAGCAGACAA TGAGCTGTGG
Lagostrophus_fasciatus    ATTGTACTTC TCTTGTCACA TGGCTCTTAT AAGCAGATGA TGAGCTGCCC
Macropus_eugenii          ATTGTATTTC TCTTATCACT TGGCTCTTAT AAGCAGACGA TGAGCTGCCG
Macropus_parma            ATTGTATTTC TCTTATCACT TGGCTCTTAT AAGCAGAGGA TGAGCTGCCA
Macropus_robustus         ATTGTATTCC TCTTATCACT TGGCTCTTAT AAGCAGACGA TGAGCTGCCG
Macropus_rufogriseus      ATTGTATTTC TCTTATCACT TGGCTCTTAT AAGCAGATGA TGAGCTGCCA
Macropus_rufus            ATTGTATTTC TCTTATCACT TGTCTCTTAT AAGCAGACGA TGAGCTGCCG
Macropus_giganteus        ATTGTATTTC TCTTATCACT TGGCTCTTAT AAGCAGACAA TGAGCTGCCG
Onychogalea_unguifera     ATTGTATTTC TCTTATCACT TGGCTCTTAT AAGCAGACAA TGAGCTGCYG
Petrogale_lateralis       ATTGTATTTC TCTTATCACT TGGCTCTTAT AAGCAGACAA TGAGCTGCTG
Potorous_tridactylus      ATTGTACTTC TTTTGTCACG TGACTCTTAT AAACAGATGA TGAGCTGCCG
Setonix_brachyurus        ATTGTATTTC TCTTATCACT TGGCTCTTAT AAGCAGACGA TGAGCTGCCT
Thylogale_stigmatica      ATTGTATTTC TCTTATCACT TGGCTCTTAT ATGCAGATGA TGAGCTGCTG
Didelphis_marsupialis     .......... .......... .......... .......... ..........
Lasiorhinus_latifrons     .......... .......... .......... .......... ..........
Petaurus_breviceps        .......... .......... .......... .......... ..........
Phascolarctos_cinereus    .......... .......... .......... .......... ..........
Pseudocheirus_peregrinus  .......... .......... .......... .......... ..........
Spilocuscus_maculatus     .......... .......... .......... .......... ..........
Trichosurus_vulpecula     .......... .......... .......... .......... ..........
Monodelphis_domestica     .......... .......... .......... .......... ..........
```

```
                         4351                                               4400
Aepyprymnus_rufescens    TGGGTTGGCT TTCATGGGAG GTGTGGTCTC GTTAAAATCT TATGGAGAAT
Dendrolagus_goodfellowi  TGGGTTGGCT TTCACAGGAG GCGTGGTCTC ATTAAAATCT TATGGAGACT
Dendrolagus_matschiei    TGGGTTGGCT TTCACAGGAG GCGTGGTCTC ATTAAAATCT TATGGAGACT
Hypsiprymnodon_moschatus TGGGTTGGCT TTCACAGGAG GCGTGGTCTC ATTAAAATCT TATGGAGAGT
Lagostrophus_fasciatus   TGGGTTGGCT TTCACGGGAG GCATGGTCTC ATTAAAGTCT TTTGGAGAAT
Macropus_eugenii         TGGGTTGGCT TTCACAGGAG GAGTGGTCTC ATTAAAATCT TATGGAGAAT
Macropus_parma           TGTGTTGGCT TTCACAGGAG GTGTGGTCTC ATTAAAATCT TATGGAGAAT
Macropus_robustus        TGGGTTGGCT TTCACAGGAG GCGTGGTCTC ATTAAAATCT TATGGAGAAT
Macropus_rufogriseus     TGGGTTGGCT TTCATGGGAG GCGTGGTCTC ATTAAAATCT TATGGAGAAT
Macropus_rufus           TGAATTGGCT TTCACAGGAG GCGTGGTCTC ATTAAAATCT TATGGAGAAT
Macropus_giganteus       TGAGTTGGCT TTCCCAGGAG GAGTGGTCTC ATTAAAATCT TATGGAGAAT
Onychogalea_unguifera    TGGGTTGGCT TTCACAGGAG GCGTGGTCTC ATTAAAATCT TATGGAGAAT
Petrogale_lateralis      TAGGTTGGCT TTCACAGGAG GCATGGTTTC ATTAAAATCT TATGGAGAAT
Potorous_tridactylus     TGGGTTGGCT TTCATGGGAG GTGTGGTCTC GTTAAAATCT TATGGAGAAT
Setonix_brachyurus       TGGGTTGGCT TTCACAGGAG GCGTGGTCTC ATTAAAATCT TATGGAGAAT
Thylogale_stigmatica     TGCGTTGGCT TTCGTAGGAG GTGTGGTCTC ATTAAAATCT TATGGAGAAT
Didelphis_marsupialis    .......... .......... .......... .......... ..........
Lasiorhinus_latifrons    .......... .......... .......... .......... ..........
Petaurus_breviceps       .......... .......... .......... .......... ..........
Phascolarctos_cinereus   .......... .......... .......... .......... ..........
Pseudocheirus_peregrinus .......... .......... .......... .......... ..........
Spilocuscus_maculatus    .......... .......... .......... .......... ..........
Trichosurus_vulpecula    .......... .......... .......... .......... ..........
Monodelphis_domestica    .......... .......... .......... .......... ..........

                         4401                                               4450
Aepyprymnus_rufescens    GTGGTCACTC TTTTGCTTGT TCTGCTTGCT CACTCTTCCT CTCCTCCAAT
Dendrolagus_goodfellowi  GTGGTCACTC CTTTGCTCCT TCTGCTTGCT CACTCTTCCT CTCCTCCAAT
Dendrolagus_matschiei    GTGGTCACTC CTTTGCTCCT TCTGCTTGCT CACTCTTCCT CTCCTCCAAT
Hypsiprymnodon_moschatus GTGGCTACTC CTTTGCT... ..TGCTCACT CACTCTTCTT CTCCTCCAAT
Lagostrophus_fasciatus   GTGGTCACTC CTTGGCTCAT TCTGCTTGTT CACTCTTCCT CTCCTCCAAA
Macropus_eugenii         GTGGTCACTC CTTTGCTCCT TCTGCTTGCT CACTCTTCCT CTCCTCCAAT
Macropus_parma           GTGGTCACTC CTTTGCTCCT TCTGCTTGCT CACTCTTCCT CTCCTCCAAT
Macropus_robustus        GTGGTCACTC CTTTGATCCT TCTGCTTACT CACTCTTCCT CTCCTCCAAT
Macropus_rufogriseus     GTGGTCACTC CTTTGCTCCT TCTGCTTGCT CACTCTTCCT CTCCTCCAAT
Macropus_rufus           GTGGTCACTC CTTTGCTCCT TCTGCTTGCT CACTCTTCCT CTCCTCCAAT
Macropus_giganteus       GTGGTCACTC CTTTGCTCCT TCTGCTTGCT CACTCTTCCT CTCCTCCAAT
Onychogalea_unguifera    GTGGTCCCTC CTTTGCTCCT TCTGCTTGCT CACTCTTCCT CTCCTCCAGT
Petrogale_lateralis      GTGGTCACTC CTTTGCTCCT TCTRTTTGCT CACTCTTCCT CTCCTCCAAT
Potorous_tridactylus     GTGGTCACTC CTTTGTTCAT TCTGCTTGCT CACTCTTCCT CTCCTCCAAT
Setonix_brachyurus       GTGGTCACTC CTTTGCTCCT TCTGCTTGCT CACTCTTCCT CTCCTCCAAT
Thylogale_stigmatica     GTGGTTACTC CTTTGCTCCT TCTGCTTGCT CACTCTTCTT CTCYTCCAAT
Didelphis_marsupialis    .......... .......... .......... ..CCCCTTCT CCCCTCCAAT
Lasiorhinus_latifrons    .......... .......... .......... ..CTTCTCCT CACCTCCAAT
Petaurus_breviceps       .......... .......... .......... ..CTCCTCCT CTCCTCTAAT
Phascolarctos_cinereus   .......... .......... .......... .....CTCCT CACCTTCAAT
Pseudocheirus_peregrinus .......... .......... .......... ..CTCCTACT CTCCTCCAAT
Spilocuscus_maculatus    .......... .......... .......... ..TTCCTCCT CTCCTCCAAT
Trichosurus_vulpecula    .......... .......... .......... ..CTCCTGCT CTCCTCCAAT
Monodelphis_domestica    .......... .......... .......... ..CCCTTCCT CACCTCCAAT

                         4451                                               4500
Aepyprymnus_rufescens    CTCCTGCCCT CTCTTTTCTC CTTCCCTTCA CTATA..... CGAG.TCTCA
Dendrolagus_goodfellowi  CTCCTCCCCT CTCTTTTCTC CTTCCCTTCA CTATA..... TGAG.TCTCA
Dendrolagus_matschiei    CTCCTCCCCT CTCTTTTCTC CTTCCCTTCA CTATA..... TGAG.TCTCA
Hypsiprymnodon_moschatus CCCTTCCCCT CTCCTTTCTC CTTCCCTTCT CTATA..... TGAGCTCTCA
Lagostrophus_fasciatus   CTCCTCCCCT CTCTTTTCTC CTTCCCTTCA CTATA..... TGAG.TCTCC
Macropus_eugenii         CTCCTCCCCT CTCTTTTCTC CTTCCCTTCA CTATA..... TGAG.TCTCA
Macropus_parma           CTCCTCCCCT CTCTTTTCTC CTTCCCTTCA CTATA..... TGAG.TCTCA
Macropus_robustus        CTCCTCCCCT CTCTTTTCTC CTTCCCTTCA CTATA..... TGAG.TCTCA
Macropus_rufogriseus     CTCCTCCCCT CTCTTTTCTC CTTCCCTTCA CTATA..... TGAG.TCTCA
```

```
Macropus_rufus              CTCCTCCCCT CTCTTTTCTC CTTCCCTTCA CTATA..... TGAG.TCTCA
Macropus_giganteus          CTCCTCCCCT CTCTTTTTTC CTTCCCTTCA CTATA..... TGAG.TCTCA
Onychogalea_unguifera       CTCCTCCCCT CTCTTTTCTC CTTCTCTTCA CTATA..... TGAG.TCTCA
Petrogale_lateralis         CTCCTCCCCT CTCTTTTCTC CTTCCCTTCA CTATA..... TGAG.TCTCA
Potorous_tridactylus        CTCCTCCCCT CTCTTTTCTC CTTCCCTTCA CTATA..... TGAG.TCTCA
Setonix_brachyurus          CTTCTCCCCT CTCTTTTCTC CTTCCCTTCA CTATA..... TGAG.TCTCA
Thylogale_stigmatica        CTCCTCCCCT CTCTTTTCTC CTTCCCTTCA CTATA..... TGAG.TCTCA
Didelphis_marsupialis       C.CCTTCCCA CTCCTTTCTC CTTCCCTTCC CTGTA..... GGAACTCTCA
Lasiorhinus_latifrons       CCCCTCCCCT TTCCTTTCTC CTTCCCTTCA CGGTACGAGC CGAGCTCCCA
Petaurus_breviceps          CCCCTCCTCT CTCCTTTCTC CTTCCCTTTA CTGTA..... TGAGCTCTCA
Phascolarctos_cinereus      CCCCTCCCCT CTCCTTTCTC CTTCCCTCCA CTGTA..... TGAGCTCTCA
Pseudocheirus_peregrinus    CCCCTCCTCT CTCCTTTCTC CTTCCCTTCA CCGTA..... TGAGCTCTCA
Spilocuscus_maculatus       TCCCTCCCCT CTCCTTTCTC CTTCCCTTCA CTGTA..... TGAGCTCTCA
Trichosurus_vulpecula       TCCCTCCCCT CTCCTTTCTC CTTCCCTTCA CTGTA..... TGAGCTCTCA
Monodelphis_domestica       CCCTTCCCAT TCCTC..... .......... CTGTA..... GGAGCTCTCA

                            4501                                               4550
Aepyprymnus_rufescens       GAATCAACCT GTGATACAGA AATTTTAATA AGGTTAGCCT AGTTTAATTT
Dendrolagus_goodfellowi     GAATCAACCT GTGATTCAGA AATTTTAATA AGGTTAGCCT AGTTTAATTT
Dendrolagus_matschiei       GAATCAACCT GTGATTCAGA AATTTTAATA AGGTTAGCCT AGTTTAATTT
Hypsiprymnodon_moschatus    GAATCAACCT GCGATTCAGA AATTTTAATA AGATTAGCCT AGTTTAATTT
Lagostrophus_fasciatus      AAATCAACCT GTGATTCAGA AATTTTAATA AGGTTAGTCT GGTTTAATTT
Macropus_eugenii            GAATCAACCT GTGATTCAGA AATTTTAATA AGGTTAGCCT AGTTTAATTT
Macropus_parma              GAATCAACCT GTAATTCAGA AACTTTAATA AGGTTAGCCT AGTTTAATTT
Macropus_robustus           GAATCAACCT GTAATTCAGA AACTTTAATA AGGTAAGCCT AGTTTAATTT
Macropus_rufogriseus        GAATCAACCT GTGATTCAGA AATTTTAATA AGGTTAGACT AGTTTAATTT
Macropus_rufus              GAATCAACCT ATAATTCAGA AATTTTAATA AGGTTAGCCT AGTTTAATTT
Macropus_giganteus          GAATCAACCT GTGATTCAGA AATTTTAATA AGGTTAGCCT AGTTTAATTT
Onychogalea_unguifera       GAATCAACCT GTGATTCAGA AATTTTAATA AGGTTAGCCT AGTTTAATTT
Petrogale_lateralis         GAATCAACCT GTGATTCAGA AATTTTAATA AGGTTAGCCT AGTTTAATTT
Potorous_tridactylus        GAATCAACCT GTGATTTAGA AATTTTAATA AGGTTAGTCT AGTTTAATTT
Setonix_brachyurus          GAATCAACCT GTGATTCAGA AATTTTAATA AGGTTAGCCT AGTTTAATTT
Thylogale_stigmatica        GAATCAACCT GTGATTCAGA AATTTTAATA AGGTTAGCCT AGTTTAATTT
Didelphis_marsupialis       GGATCAACCT GTGATTCAAA AGTTTTAATA AGGTTAGTCT GGTTTACTTT
Lasiorhinus_latifrons       GAAGCAACCT GTGATTCAGA AGTTTTGATA AGGTTAGCCT AGTTTAATTT
Petaurus_breviceps          GAATCAACCT GTGATTCAGA AATTATAATA AGGTTAGCCT AGTTTAATTT
Phascolarctos_cinereus      GAAGCAACCT GTGATTCAGA AGTTTTAATA AGGTTAGCCT ATTTTAATTT
Pseudocheirus_peregrinus    GAATCAACTT GTGATTCAGA AATTTTAATA AGGTTAGCCT AGTTTAATTT
Spilocuscus_maculatus       GAATCAACCT GTGATTGAGA AATGTTAATA AGGTTGGCCT AGTTTAATTT
Trichosurus_vulpecula       GAATCAACCT GTGATTCAAA AATTTTAATA AGGTTAGCCT AGTTTAATTT
Monodelphis_domestica       GGACCAACGT GCGATTCCAA AGTTTTAATA AGGTTAGCCT AGTTTAATTG

                            4551                                               4600
Aepyprymnus_rufescens       GAGTGGATTT GAAGCCTCTG TTGTCACAAT CAAAGAAGCA TTCACATTAC
Dendrolagus_goodfellowi     GAGTGGATTT GAAAYCTCTG TTGTGACAAT CAAAGAAGCA TTCACATTAC
Dendrolagus_matschiei       GAGTGGATTT GAAACCTCTG TTGTGACAAT CAAAGAAGCA TTCACATTAC
Hypsiprymnodon_moschatus    GAGTGGATTT GAAACCTCTG TTGTCACAAT CAAAGAAGCA TTCACATTAC
Lagostrophus_fasciatus      GAGTGGATTT GACTCCTCTG TTGTCACAAT CAAAGAAGCA TTCACATTAC
Macropus_eugenii            GAGTGGATTT GAAACTTCTG TTGTCACAAT CAAAGAAGCA TTCACATTAC
Macropus_parma              GAGTGGATTT GAAACTTCTG TTGTCACAAT CAAAGAAGCA TTCACATTAC
Macropus_robustus           GAGTGGATTT GAAACTTCTG TTGTCACAAT CAAAGAAGCA TTCACATTAC
Macropus_rufogriseus        GAGTGGATTT GAAACTTCTG TTGTCACAAT CAAAGAAGCA TTCACATTAC
Macropus_rufus              GAGTGGATTT GAAACTTCTG TTGTCACAAT CAAAGAAGCA CTCACATTAC
Macropus_giganteus          GAGTGGATTT GAAACTTCTG TTGTCACAAT CAAAGAAGCA TTCACATTAC
Onychogalea_unguifera       GAGTGGATTT GAAACTTCTG TTGTCACAAT CAAAGAAGCA TTCACATTAC
Petrogale_lateralis         GAGTGGATTT GAAACCTCTG TTGTCACAAT CAAAGAAGCA CTCACATTAC
Potorous_tridactylus        GAGTGGATTT GAAACCTCTG TTGTCACAAT CAAAGAAGCA TTCATATTAC
Setonix_brachyurus          GAGTGGATTT GAAACTTCTG TTGTCACAAT CAAAGAAGCA TTCACATTAC
Thylogale_stigmatica        GAGTGGATTT GAAACCTCTG TTATCACAAT CAAAGAAGCA TTCACATTAC
Didelphis_marsupialis       GAGTGGGTTT GAAACCTCTG TTGTCATAAT CAAAGAAGCA TTCGCGTTAC
Lasiorhinus_latifrons       GAGTGGATTT AAAACCTCTG CTGTCACAAT CAAAGAAGTA TTCACATTAC
Petaurus_breviceps          GAGTGTATTT GAAACCTCTG TTGTCAAAAT CAAAGAAGCA TTCACATTAC
Phascolarctos_cinereus      GAGTGGATTT GAAACCTCTG TTGTCACAAT CAAAGAGGCA TTCACATTAC
```

```
Pseudocheirus_peregrinus GAGTGGATTT GAAACCTCTG TTGTCACAAT CAAAGAAGCA TTCACATTAC
Spilocuscus_maculatus    GAGTGGATTT GAAACCTCTG TTGTCACAAT CAAAGAAGCA TTCATATTAC
Trichosurus_vulpecula    GAGTGGATTT GAAACCTCTG TTGTCACAAT CAAAGAAGCA TTCACATTAC
Monodelphis_domestica    GAGTGGGCTT GAAACCTCTG TTGTCATAAT CAAAGAAGCA TTCATGTTAC

                         4601                                             4650
Aepyprymnus_rufescens    CAAATTAACA AGGCTTATTA GGAGAGTGGC CTACTACTGA AATCCTTTGA
Dendrolagus_goodfellowi  CAAATTAACA GGGCTTATTA GGAGAGTGGC CTACTACTGA AGTCCTTTGA
Dendrolagus_matschiei    CAAATTAACA GGGCTTATTA GGAGAGTGGC CTACTACTGA AGTCCTTTGA
Hypsiprymnodon_moschatus CAAATTAACA GGGCTTATTA GGAGAGTGGC GAACTACTGA AGTCCTTTGA
Lagostrophus_fasciatus   CAAATTAACA GGGCTTATTA GGAGAGTGGC CTACTACTGA AGTCCTTTGA
Macropus_eugenii         CAAATTAACA GGGCTTATTA GGAGAGTGGC CTATTACTGA AGTCCTTTGA
Macropus_parma           CAAATTAACA GGGCTTATTA GGAGAGTGGC TTACTACTGA AGTCCTTTGA
Macropus_robustus        CAAATTAACA GGGCTTATTA GGAGAGTGGC TTACTACTGA AGTCCTTTGA
Macropus_rufogriseus     CAAATTAACA GGGCTTATTA GGAGAGTGGC CTACTACTGA AGTCCTTTGA
Macropus_rufus           CAAATTAACA GGGCTTATTA GGAGAGTGGC TTACTACTGA AGTCCTTTGA
Macropus_giganteus       CAAATTAACA GGGCTTATTA GGAGAGTGGC CTACTACTGA AGTCCTTTGA
Onychogalea_unguifera    CAAATTAACA GGGCTTATTA GGAGAGTGGC TTACTAGTGA AGTCCTTTGA
Petrogale_lateralis      CAAATTAACA GGGCTTATTA AGAGAGTGGT CTACTACTGA AGTCCTTTGA
Potorous_tridactylus     CAAATTAACA GGGCTTATTA GGAGAGTGGC CTACTACTGA AGTCCTTTGA
Setonix_brachyurus       CAAATTAACA GGGCTTATTA GGAGAGTGGC CTACTACTGA AGTCCTTTGA
Thylogale_stigmatica     CAAATTAACA GGGCTTATTA GGAGAGTGGC CTACTACTGA AGTCCTTTGA
Didelphis_marsupialis    CAAATTAACA GGGCTTATTA GGAGAGTGGC CGACTACTGA AGTCCTTTGA
Lasiorhinus_latifrons    CAAATTAACA GGGCTTATTA GGAGAGTGGC CGACTACCGA AATCTTTTGA
Petaurus_breviceps       CAAATTAACA AGGCTTATTA GGAGAGTGGC CGACTACTGA AGTCCTTTGA
Phascolarctos_cinereus   CAAATTAACA GGGCTTATTA GGAGAGTGGC CGACTACTGA AGTCCTTTGA
Pseudocheirus_peregrinus CAAATTAACA GGGCTTATTA GGAGAGTGGC CTACTACTGA AGTCCTTTGA
Spilocuscus_maculatus    CAAATTAACA GGGCTTATTA GAAGAGTGGC CGACTACTGA AGTCCTTTGA
Trichosurus_vulpecula    CAAATTAACA GGGCTTATTA GGAGAGTGGC CGACTACGGA AGTCCTTTGA
Monodelphis_domestica    CAAATTAACG GGGCTTATTA GGAGAGTGGC CGAGTGCTGA AGTCCTTTGA

                         4651                                 4689
Aepyprymnus_rufescens    ACACTCAGCC ATATGGGTAT ATCAAGACCA GATCAAACA
Dendrolagus_goodfellowi  ACACTCAGCC ATGTGGGTAT ATCAAGACCA GATCAAACA
Dendrolagus_matschiei    ACACTCAGCC ATGTGGGTAT ATCAAGACCA GATCAAACA
Hypsiprymnodon_moschatus ACACTCAGCC ATATGGGTAT ATCAAGACCA GATCAAACA
Lagostrophus_fasciatus   ACACTAAGTC ATATGGGTAT ATCAAGACCA GATCAAATA
Macropus_eugenii         ACACTCAGCC ATGTGGGTAT ATCAAGACCA GATCAAACA
Macropus_parma           ACACTCAGCC ATGTAGGTAT ATCAAGACCA GATCAAACA
Macropus_robustus        ACACTCAGCC ATGTGGGTAT ATCAAGACCA GATCAAACA
Macropus_rufogriseus     ACACTCAGCC ATGTGGGTAT ATCAAGACCA GATCAAACA
Macropus_rufus           ACACTCAGCC ATGTGGGTAT ATCAAGACCA GATCAAACA
Macropus_giganteus       ACACTCAGCC ATGTGGGTAT ATCAAGACCA GATCAAACA
Onychogalea_unguifera    ACACTCAGCC ATGTGGGTAT ATCAAGACCA GATCAAACA
Petrogale_lateralis      ACACTCAGCA ATGTGGGTAT ATCAAGATCA GATCAAACA
Potorous_tridactylus     ACACTCAGCC ATATGGGTAT ATCAAGACCA GATCAAACA
Setonix_brachyurus       ACACTCAGCC ATGTGGGTAT ATCAAGACCA GATCAAACA
Thylogale_stigmatica     ACACTCAGCC ATGTGGGTAT ATCAAGACCA GATCAAACA
Didelphis_marsupialis    ACACCCAGAC ATATGGATAT CTCAAGAACA GATCAAACA
Lasiorhinus_latifrons    ACACTCAGCC ATGTGGGTAT ATCAAGACCA GATCAAACA
Petaurus_breviceps       ACACTCAGCC ATATGGGTTT ATCAAGACCA GATCAAACA
Phascolarctos_cinereus   ACACTCAGCC ATATGGGTAT ATCAAGACCA GATCAAACA
Pseudocheirus_peregrinus ACACTCAGCC ATATGGGTAT ATCAAGACCA GATCAAACA
Spilocuscus_maculatus    ACACTGAGCC ATATGGGTAT ACCAAGACCA GATCAAACA
Trichosurus_vulpecula    ACACCGAGCC ATATGGGTAT ATCAAGACCA GATCAAGCA
Monodelphis_domestica    ACACCCAGCC ATATGGGTAT ATCAAGAAGA GATCAAACA
```

Supplementary Figure S1. Multiple sequence alignment of sixteen macropodoid mAAV-EVE1 loci along with "empty" loci from eight closely-related non-macropodoid taxa. Sequences were aligned using the MUSCLE multiple sequence alignment tool as implemented in MEGA6.06. Alignment gaps are indicated by a dot ("."). See the Methods section for alignment settings.

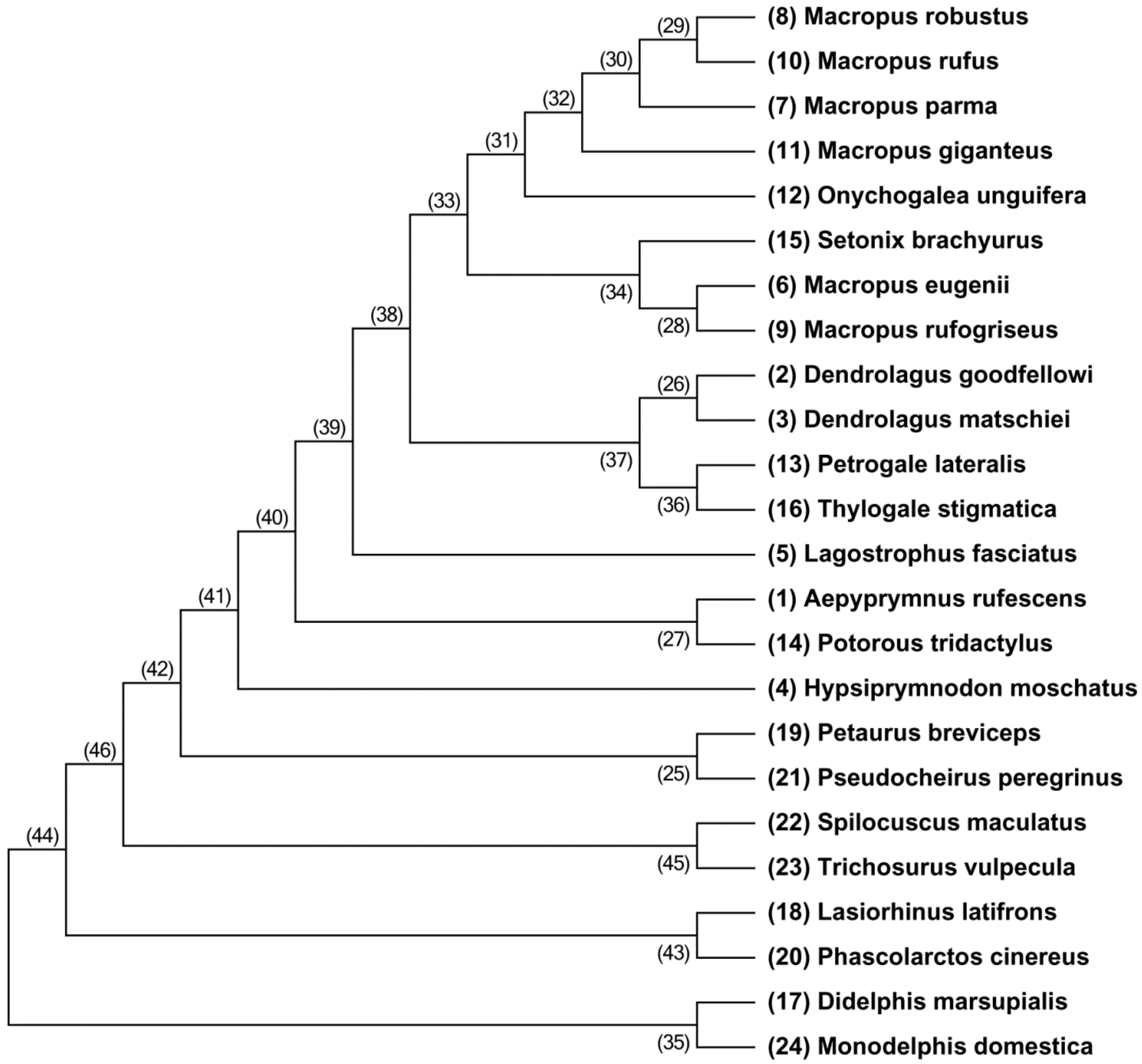

Supplementary Figure S2. Ancestral sequence inference - node identification. Cladogram depicting phylogenetic relationships among mAAV-EVE1 loci used for ancestral sequence inference. Both AAV-EVE-bearing and “empty” loci (outgroup) are indicated. Due to central deletions within *H. moschatus* and potoroid (*A. rufescens*, and *P. tridactylus*) EVE sequences relative to the remainder of the dataset (see Fig. 3), the node corresponding to the full-length mAAV sequence inference is Node 39. This node dates to approximately 14 MYA.

```
   1 aaatcctaga ttatgagagc cagcgatcaa agaagtggcc acactctcca taagatttga aaagcccgcc aaagcagatg atgtaattac
  91 ccataatgca attggaatca gtcccagact gcattgcaag aggctataaa aagaagggtg tatgcctaga aattcattaa tcatctaccg
 181 actgagcgag tgtgcatcaa aagaagaaga ataaatagaa gagatgaaga catgctggag ctggaacacg tgaagtttta tgaggcaatt
 271 ttccttgtgc ccagagactt ggagtctgac atccctggct atcctaagag actggtcact cagatagaag agaccaagtg gacactttca
 361 gaaaaggacg acctggactt ggaagcggtg gagagtggac aggtaacatt tgcccatcta ttctcccgca aattccttaa gcactgggag
 451 tacctgacaa gaaatcgaga attcaaatac tatgtccagc tgaaaaaggg tgagatctat taccatttac atatgctttt tgagaccagt
 541 ggaattcagt ccatggtgct cagccgttac atcagccaga tcaagacctc gctgcaagct gaagtctcta acaatgctga ggttaatatc
 631 gaaaactgac tggccattac gacgactaag gccaccgggg ggggtcaacc aaacaggtgg actataattg catcaactgg tatttaatgg
 721 cataaaaaca accagaattt cagtgggtgt ggacaaatat tgaggaatat aaggacttga tcctcaatat ccccgccaga ctgcagctcg
 811 caggctagtt cttcacatcg acctacttgg ctcctggggt gagtgactcc caatcctctt aaatctctaa tacttctggt gctcccttct
 901 attgtgatag aaataccgag agatacatgg agcttgtaaa ttggcttgtg gagcagcacc tctgaaaagc agtggattat tgaaaatcag
 991 gaaagttatc tctctcacca atccactagc aatggagcca ggcagattaa agtcgctctg gacaatgctt ctaaaattat gaatctgacc
1081 aaaaatgcag atgattacct tatcccgaaa gaatttgtca gttttgacaa cattaaacag aaccatatct attggatctt taagaatgga
1171 tacgaccctc tttatgctgg atctatcctg gtgggctggg ccagaaaaga atttggcaaa agaaacacca tctggttcta tggaaaggcc
1261 actactggga acactaacat tgcggaagcc gttgcacaca cggtgccctt atacgggtat gtgaattgga ctaatgagaa ctttccattc
1351 aatgactgtg tggataaaat gctcatctgg tgggaggaag gcgagattac ctctaaggtg gttgagacag ctaaagccat ccttggagga
1441 gctaaagttc aggtggacca ggaatgtaag tcctctgttc aaattgattg tactccagtc atcatcacct ccaacaccaa catgtgctac
1531 atggtggacc ggaacactat gatctttgaa cacaagcagt tgttacaaga ccacatgttt caattcatgc tcatggagag acttcctgat
1621 gactttggca aggtgacaaa ggaggaggtg cgtcagttct tttaaatggg cagctgttaa tcaaattccc cctaagcagg aattcactgt
1711 caagaagatt atgtcatcca ttgactgtta ctatgaccac aagcagaaat gggaggagta tccctcgact ttctgtcaag gggctataa
1801 aaaggccgag cctccctcga aaaagttctt tccattccgg gatttgaaga aaattgaggt catcgagcag agagccccca cagtggaatc
1891 ggactttgag aatctcaaaa ggtttggtct caatgtaatt cccgcttctg atcctattgc tttggatgac tgtcaggatg agcaataaat
1981 ccgtagatgt ctttttttgga gaaatttgag gactggtacg aaaagtcagc tgctacttgg agacaccttg aagctggccc acctcatcct
2071 aaagctaatc aacaacatca agatgactct catggactgg ttctgccagg ctataagtat ctcattccct ttaatggtct ctataagggg
2161 gagccagtta atcaagcaga caaagccaca ctggaacaag agaaagccta cgatcaattc ctcaaagaag ggaaaatcc ttacctcacc
2251 tacaaccaca cagaccaaga gttccaggaa aaactttcgg aggacacttc gtttggtggt aaccttggca aggcagtgtt tcaaggaaag
2341 aaatgactgc ttaagccatt aggagtagta gaaccagacc tggagcctgt gaaaggagaa actcctgaga agctgtgcat ccctcagcaa
2431 ctccaaatcc ctcctcctcc atctaagcga caaaagacga gaggactccc tttcaaccca aacagcgaca atggagcata caccagcagt
2521 cagcaatcag cccccactaa tttgggatct ggtatcatgg cagaaggagg tggcgcacca atgggcaata atcaacaggg tgctgatgga
2611 gtaggtaatt cctcaggaaa ttagcattgt gattcccaat ggatgggcca cagagtcgcc acccgaaaaa ctctctgggt cttgcccacc
2701 tgcaacaacc acctctacaa gcagttcaaa acagtgtcac cacaggcagt gccaacaact actttggctt cagcaccccc tgggggtatt
2791 ttgacttcaa cagattccac tgccacttca gcccctgaga ctggcaaaga cttatcaata acaactgggg actgcgacct aaaaacctgc
2881 acttcaaact cttcaacatc caagtcaagg aggtcacaag gaggaatgtt gagaccacaa ttgctaataa ccttaccagc atgattcaag
2971 tctttgcgga ctcagtgtca actcccatac atgatcagga gtgctcaaga ggggtgtcta ctccccttcc ctcctgatgt gtttatgttg
3061 cctcagtatg ggtattgtac tttggacaat gatgggaaaa gtttagagag gagtgcattc tactgtctag aatattttcc tagccaaata
3151 ttgagaacgg gtaacaactt tgaattttcc tatgcttttg aatctgtccc ctttcatagc atgtggatgc ataatcagag cttggataga
3241 ttgatgaatc cattgattga tcaatatctg tatagatttg ataatctaac cagtggaaac actgttaatc ccaccttcac ttacaaaaag
3331 ggatcagcag gtgatatggc ttctcaggct aggaattggt tacctggtcc tatgcttagg aatcagggac taaaggatgg tcctaacaat
3421 caggccaatc tagatggttg gaggatcagt cctccaatgg tgatcaatgg aaaatcttct attatatttc ctgggccatc catgtatacc
3511 gcacacaatg ctgcagatga actggaggtt caacctagca ttaatctccc tatctttgct aaagatgcct ctgtacctga atccaccata
3601 attagtagta ttggtaatca agatcctaat agtaaattgt tagtcactga tgagaacgag gtcgggacag tgaatgctac tgctgctaat
3691 acctgggggt ctatggcagt caaccagcag actcccaccc ccactagtgc aggacaggtt ctaaatcaaa tgagtgtcat gcctggaatg
3781 gtctggcaga atagagacat cgatctccat ggtcccattt gggctaagat tcctcacaca gatggttact tccatccctc tcctctcatg
3871 ggtggctttg gtctcaaaca tcctcctcct cagaatatga ttaaaaacac tcctgtccct gctaaccctg ccaccatctt cactcctgtc
3961 aaacaaaatt ctttcatcac tcaatactct actggtcaag tgactgtaga aattgaatgg gaactccata aggaaagttc caagaaatgg
4051 actcctgaaa tccagtttac ttccaatttc agaaacacta ttgacttacc ttttgctccc aacaatgaag gtgtatactc tgaacctcgt
4141 cccattggta cccgatacct taccctttccc atctaactgt attgtacata tttcatattt gtattttttt attcaataaa ctgatttatt
4231 catttcattg tacttctctt gtcacgtggc tcttataagc agatgatgag ctgccgtggg ttggctttca cgggaggcgt ggtctcatta
4321 aaatcttatg gagaatgtgg tcactccttt gctcattctg cttgctcact cttcctctcc tccaatctcc tcccctctct tttctccttc
4411 ccttcactat atgagtctca gaatcaacct gtgattcaga aattttaata aggttagcct agtttaattt gagtggattt gaaacctctg
4501 ttgtcacaat caaagaagca ttcacattac caaattaaca gggcttatta ggagagtggc ctactactga agtcctttga acactcagcc
4591 atatgggtat atcaagacca gatcaaaca
```

Supplementary Figure S3. Inferred ancestral sequence. Unedited mAAV-EVE1 ancestral sequence from Node 39 of the mAAV-EVE1 maximum likelihood evolutionary tree.

Supplementary Table S1. Manual edits to maximum likelihood ancestral sequence (Node 39)

mAAV-EVE gene region	Node 39 nucleotide position and manual substitution	Macropodoid taxa already bearing the indicated substitution
rep	493 (A -> G)	*H. moschatus*
rep	639 (A -> G)	*D. goodfellowi, D. matschiei, P. lateralis, T. stigmatica*
rep	653 (C -> A))	*H. moschatus*
rep	667, 668 (delete GG)	*N/A*
rep	700 (G ->A)	*H. moschatus, S. brachyurus*
rep	717 (A -> G)	*H. moschatus*
rep	723 (T -> A)	*H. moschatus*
rep	748 (T -> C)	*H. moschatus*
rep	816 (T -> C)	*H. moschatus*
rep	870 (T -> C)	*H. moschatus*
rep	957 (insert TT)<br>arbitrary frameshift correction	*N/A*
rep	1540 (C -> G)	*L. fasciatus*
rep	1659 (delete C)	*D. goodfellowi, D. matschiei, P. lateralis, T. stigmatica*
cap	2344 (T -> C)	*L. fasciatus*
cap	2416 (T -> C)	*L. fasciatus, P. lateralis*
cap	2613 (A -> G)	*H. moschatus*
cap	2633 (A -> G)	*H. moschatus*
cap	2684 (replace T with ACAC)	*H. moschatus*
cap	2702 (G -> A)	*H. moschatus*
cap	2722 (insert G)	*H. moschatus*
cap	2826 (T -> C)	*H. moschatus, M. parma, M. robutus, M. rufogriseus, M. rufus, M. giganteus, O. unguifera, P. lateralis, T. stigmatica*
cap	2962 (T -> C)	*H. moschatus*
cap	2985 (insert AG)	*All dataset members have an AV dinucleotide at this position*
cap	2987 (G -> A)	*H. moschatus*
cap	3001 (A -> G)	*H. moschatus,*<br>*D. goodfellowi, D. matschiei, P. lateralis, T. stigmatica, M. eugenii, M. parma, M. robutus, M. rufogriseus, M. rufus, M. giganteus, O. unguifera, P. lateralis, T. stigmatica*
cap	3150 (A -> G)	*H. moschatus, P. tridactylus*
cap	3404 (A -> T)	*H. moschatus*
cap	3905 (A -> T)	*H. moschatus*
cap	4029 (T -> G)	*H. moschatus*
cap	4037 (G -> C)	*H. moschatus*
cap	4038 (T -> C)	*H. moschatus*
cap	4166 (T -> G)	*H. moschatus*
